# Synthesis of a donor–acceptor heterodimer via trifunctional completive self-sorting

**DOI:** 10.1038/s41467-022-30859-7

**Published:** 2022-06-09

**Authors:** Sunit Kumar, Yogesh Kumar Maurya, Tadeusz Lis, Marcin Stępień

**Affiliations:** Wydział Chemii, Uniwersytet Wrocławski, ul. F. Joliot-Curie 14, 50-383 Wrocław, Poland

**Keywords:** Self-assembly, Structure elucidation

## Abstract

Selective self-assembly of heterodimers consisting of two non-identical subunits plays important roles in Nature but is rarely encountered in synthetic supramolecular systems. Here we show that photocleavage of a donor–acceptor porphyrin complex produces an heterodimeric structure with surprising selectivity. The system forms via a multi-step sequence that starts with an oxidative ring opening, which produces an equimolar mixture of two isomeric degradation products (zinc(II) bilatrien-*abc*-ones, BTOs). These two isomers are susceptible to water addition, yielding the corresponding zinc(II) 15-hydroxybiladien-*ab*-ones (HBDOs). However, in the photocleavage experiment only one HBDO isomer is formed, and it quantitatively combines with the remaining BTO isomer. The resulting heterodimer is stabilized by a Zn–O coordination bond and extended dispersion interactions between the overlapping π-surfaces of the monomers. The observed selectivity can be seen as a case of completive self-sorting, simultaneously controlled by three types of complementary interactions.

## Abstract (web summary)

The preparation of heterodimeric structures via self-assembly processes is challenging. Here, the authors report the photooxidation of a donor–acceptor metalloporphyrin, which enables a self-sorting process that yields an heterodimer quantitatively.

## Introduction

Oligomerization of functional monomers enables rapid synthesis of large structures from smaller components and plays an important role in supramolecular and biomolecular self-organization. Self-assembly of identical subunits can produce rapid increase in molecular size, however, the high symmetry of the resulting constructs limits their potential function. At the same time, multicomponent self-assembly plays important roles in biological systems, e.g. in allosteric regulation^1^. In supramolecular chemistry, interactions between non-equivalent subunits are usually controlled by social self-sorting algorithms^2,3^, which employ libraries of carefully designed components containing multiple orthogonal binding sites, and can be used to create complex structures, such as capsules^4^, polymers^5,6^, or gels^7^. Formation of heterodimers may be seen as the simplest case of multicomponent self-assembly, with important examples among enzymes and protein receptors^8,9^. In supramolecular chemistry, heterodimers may act e.g. as energy transfer systems^10^, however, their formation under equilibrium conditions relies on additional components^10–12^ or specific engineering of interaction sites^13,14^, and is seldom quantitative^10^.

**Fig. 1:**
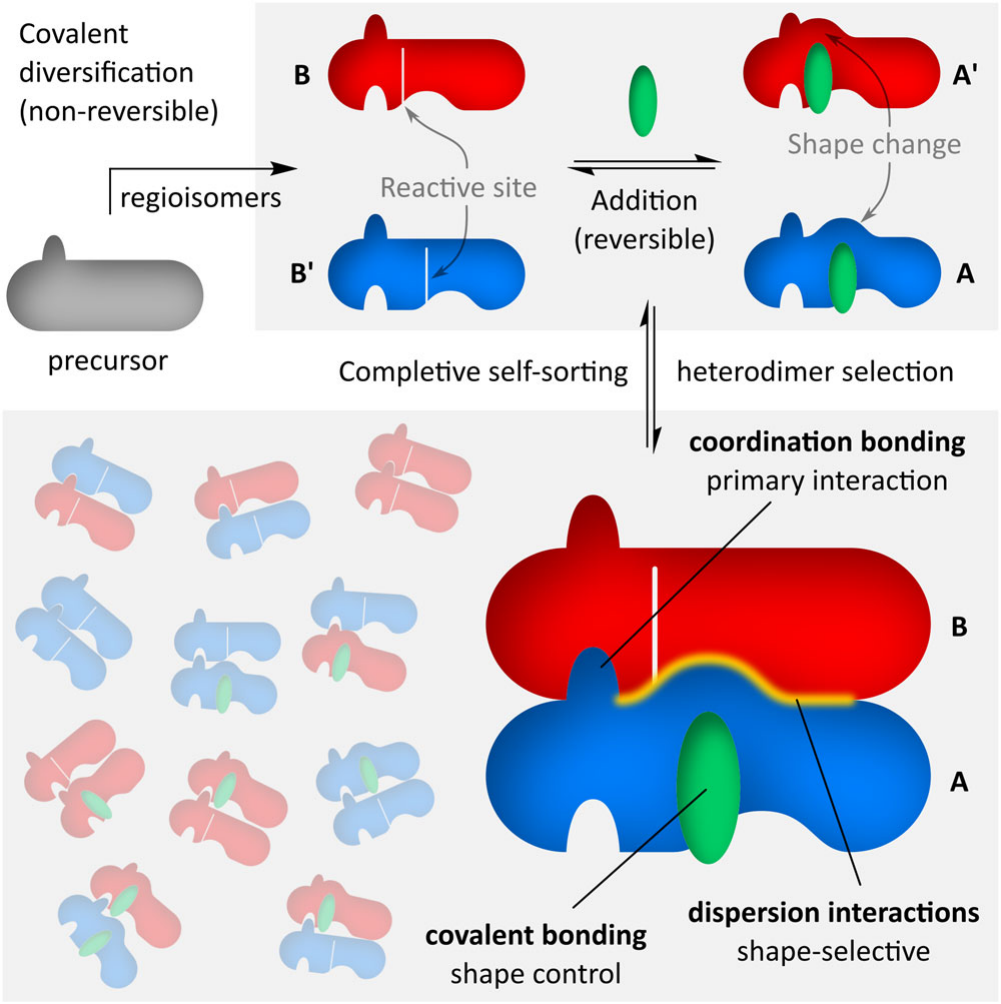
Trifunctional completive self-sorting of a bichromophoric heterodimer. Red and blue objects correspond to regioisomeric products of covalent diversification of the precursor (gray). The green object represents a small molecule which binds the initially formed **B** and **B’** to induce a shape change, which results in exclusive shape complementarity of **A** and **B** and selective formation of the **AB** heterodimer.

Generation of low-symmetry assemblies from a single precursor is even more challenging, because binding specificity must emerge in situ from initially identical components. Here we show an example of such a transformation, which selectively converts a single species into a bichromophoric heterodimer (Fig. 1). The process begins with initial covalent diversification, which consists of a non-reversible cleavage of the precursor followed by a formally reversible addition reaction. The addition is biased by subsequent self-assembly, which quantitatively produces a heterodimeric structure. The overall process can be described as a case of completive social self-sorting^15,16^ that exploits three distinct interaction types: reversible covalent addition, metal–ligand coordination, and dispersive forces.

## Results and discussion

### Formation and structure of 2

**Fig. 2:**
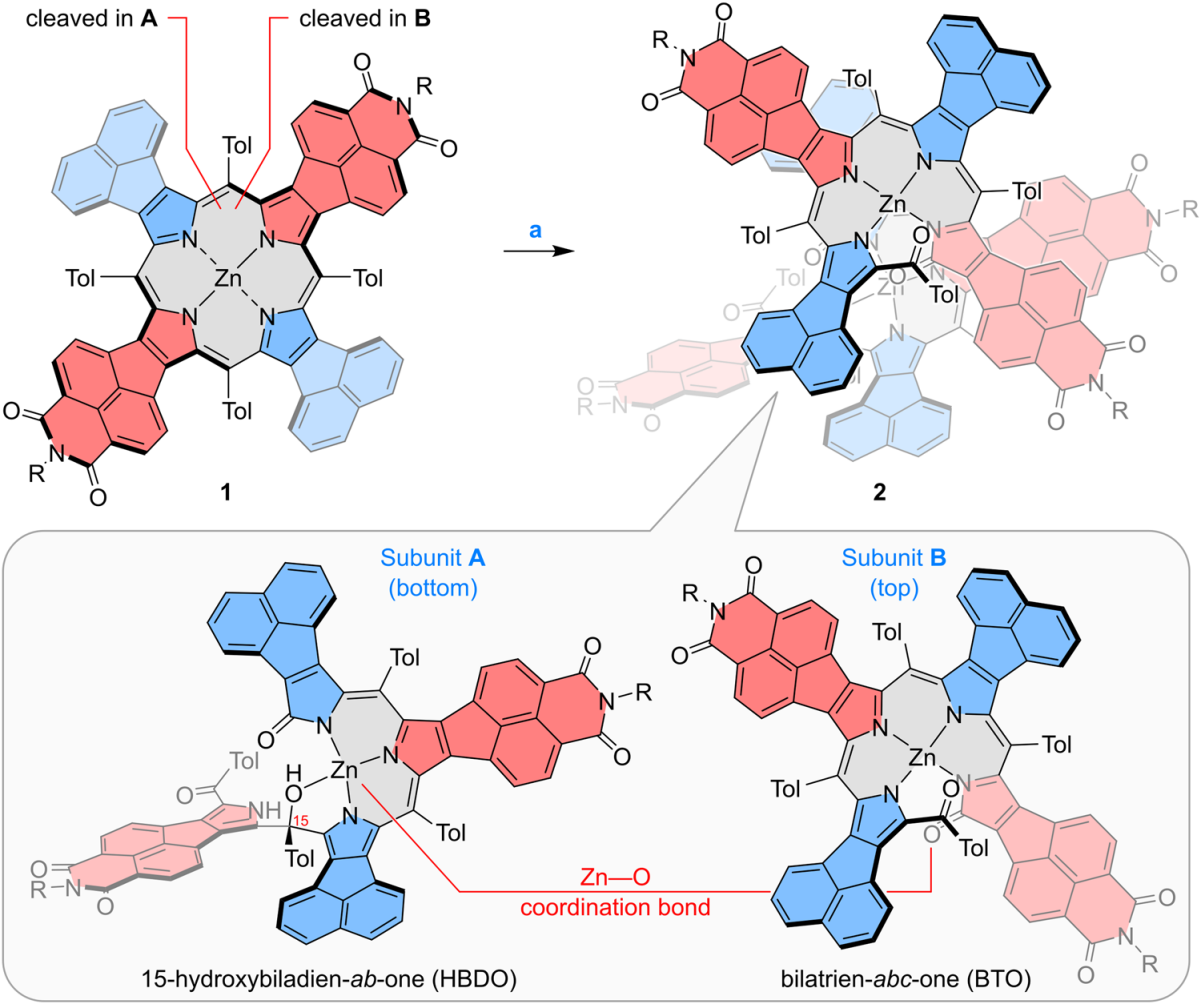
Single-step conversion of **1** into heterodimer **2**. Reagents and conditions: (a) toluene in air, visible light irradiation. Acceptor and donor pyrrole subunits are highlighted in red and blue, respectively. R = 2,6-diisopropylphenyl.

**Fig. 3:**
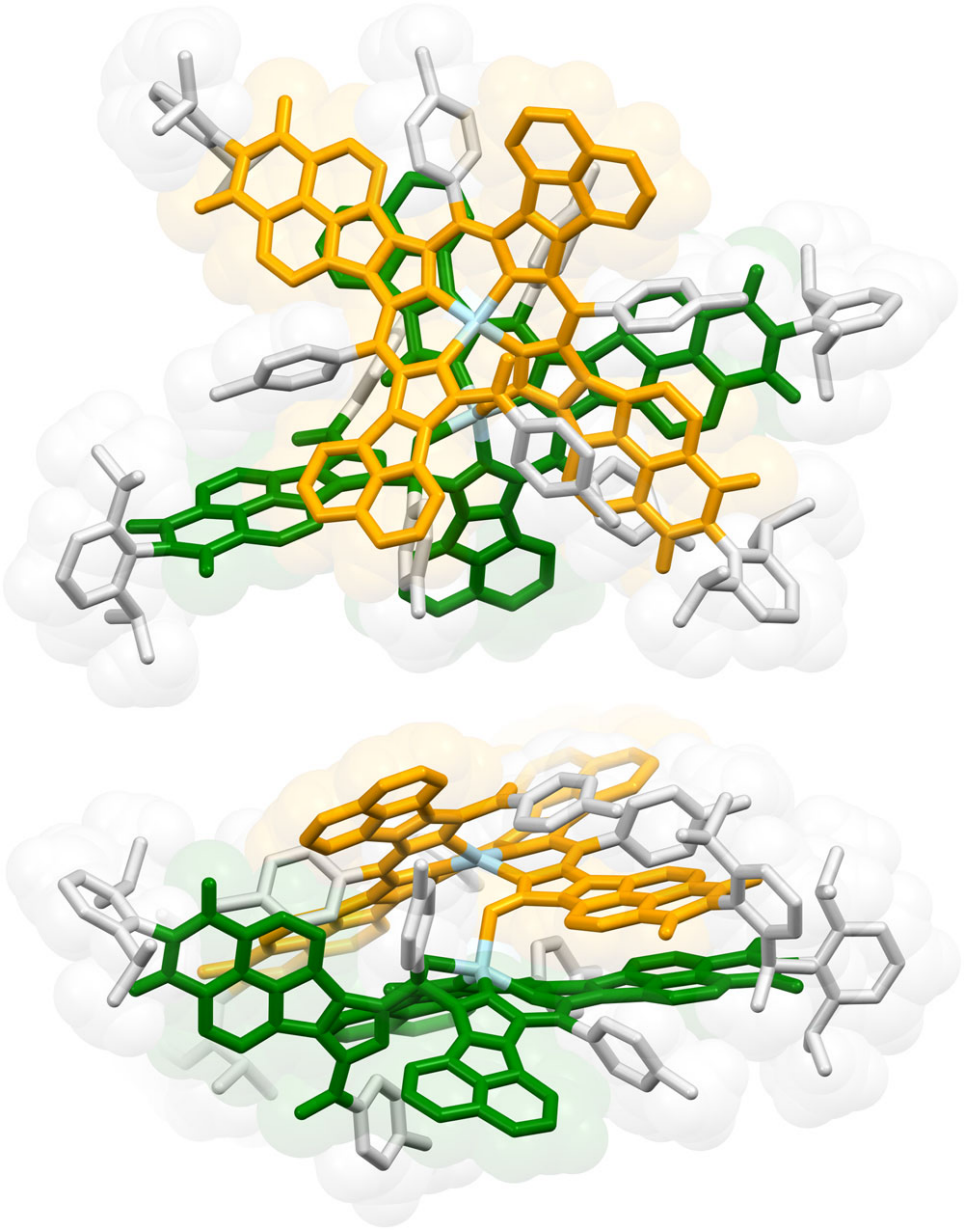
Molecular structure of dimer **2** obtained in an X-ray crystallographic analysis. Hydrogen atoms and solvent molecules are omitted for the clarity. Subunits **A** and **B** and zinc atoms are shown in green, orange, and light blue, respectively. 2,6-Diisopropylphenyl (dipp) and *para*-tolyl (Tol) substituents are shown in light gray. See Supplementary Fig. 3 for an ORTEP representation of this structure.

The above reaction was discovered during our work on the π-extended zinc(II) porphyrin **1**^17^, a hybrid system containing two types of pyrrole subunits: naphthalene-fused donors and naphthaleneimide-fused (NMI) acceptors^18^. We noticed that the compound underwent a gradual chemical transformation when stored in a toluene solution under ambient conditions. Single crystals of the product obtained by evaporation of the solvent were subjected to X-ray diffraction analysis, which revealed an unprecedented heterodimeric structure **2** (Fig. 2, Fig. 3). The structure of **2** consists of two non-equivalent subunits, **A** and **B**, each containing a zinc(II) center and a linear tetrapyrrole ligand formed by oxidative cleavage of a C_meso_–C_α_ bond. Interestingly, the two subunits are very different: in subunit **B**, the macrocycle was cleaved next to the acceptor unit and remained in the bilatrien-*abc*-one (BTO) form, which acts as an N_4_ ligand for zinc(II)^19,20^. The ligand is helical and provides a distorted square-planar environment for Zn^II^, with Zn—N distances of 1.973(8) to 2.008(9) Å. In subunit **A**, cleavage occurred next to the electron-rich pyrrole unit and was followed by addition of a water molecule, to produce a π-extended 15-hydroxybiladien-*ab*-one (HBDO), which provides a N_3_O coordination environment for Zn^21^. In particular, the OH group forms a somewhat elongated Zn—O bond (2.194(6) Å), and a hydrogen bond to the lactam terminus of the **A** unit (O···O, 2.53 Å). 15-Hydroxybiladien-*ab*-ones are formal hydration products of bilatrien-*abc*-ones and interconversion between these two species has been demonstrated for both free-base HBDOs^22,23^ and their Zn^II^ complexes^20^. In the molecule of **2**, the hydration status differs in subunits **A** and **B**, and is apparently correlated with the position of the cleavage. The two subunits are held together by a coordination bond between the lactam oxygen of unit **B** and the Zn^II^ center of unit **A**. This bond, with a length of 2.002(8) Å, completes the distorted trigonal bipyramid environment around Zn^II^ in **A**.

Subsequently, a stirred 3 mM solution of **1** in toluene-*d*_8_ was irradiated with a 4000 K consumer LED source and the progress of the reaction was monitored with ^1^H NMR spectroscopy. Remarkably, the formation of **2** was found to be effectively quantitative after 4 h, with no side-products or intermediates observable by NMR. The ^1^H NMR spectrum of **2** revealed a complex spectral pattern, which was analyzed using 2D correlation methods (Supplementary Fig. 8–14). In particular, **2** has eight non-equivalent diastereotopic isopropyl groups and eight non-equivalent tolyl groups, in line with the *C*_1_ molecular symmetry of the assembly. A narrow resonance at 14.34 ppm was identified as the hydrogen-bonded OH group of subunit **A**, whereas a broader NH peak of the pendant NMI pyrrole was found at 10.13 ppm. Characteristically, several signals of the aromatic naphthalene subunits have strongly upfield shifts in the ca. 3.7–5.4 ppm range, as a consequence of the combined shielding caused by neighboring tolyl substituents and the stacked π systems of the two subunits. A ^1^H DOSY spectrum recorded for a partly converted sample showed a decrease of the diffusion coefficient of **2** relative to that of **1**, consistent with approximate doubling of the molecular weight (Supplementary Fig. 7). Mass spectrometric analyses revealed facile dissociation of **2** in ion sources. Specifically, a peak at *m*/*z* 1719.58 was found in a positive-ion MALDI spectrum, corresponding to [**B** + H]^+^ (Supplementary Fig. 22), whereas a peak at *m*/*z* 1735.53 was observed in a negative-mode ESI spectrum, which was identified as [**A** – H]^−^ (Supplementary Fig. 23). The latter ionization mode is consistent with the presence of dissociable protons on the A subunit.

### Analysis of reactivity

**Fig. 4:**
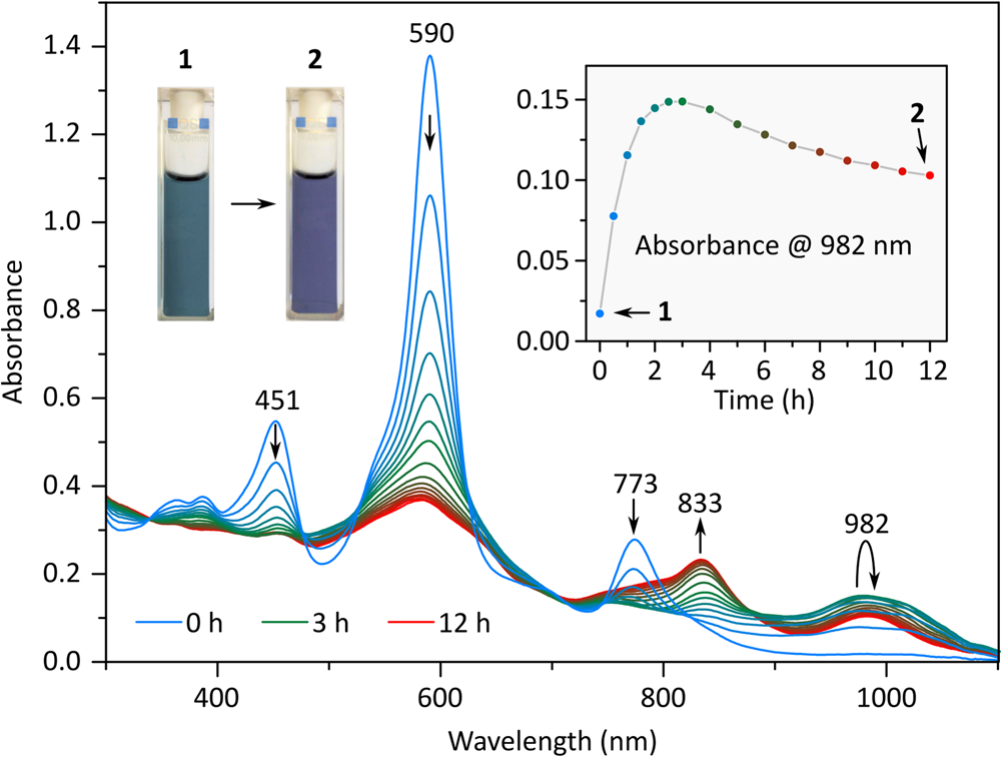
Changes of UV-vis-NIR absorption spectra occurring during photoirradiation of **1**. Blue: initial spectrum of **1** (0.01 mM in toluene, room temperature); green: intermediate photooxidation state with maximum intensity at 982 nm (*t* = 3 h); red: final absorption spectrum of **2** (*t* = 12 h). Spectra were recorded every 1 h.

When a more dilute solution of **1** was irradiated (0.01 mM in toluene), a two-step transformation could be observed using absorption spectroscopy (Fig. 4). In the initial, faster step, occurring during the first 3 h of irradiation, the Soret band of **1** was observed to decrease in intensity, while a new set of absorptions appeared in the near infrared range (700 to 1100 nm). In the second step, which could be driven to completion after 12 h of irradiation, the band at ca. 982 nm decreased in intensity, without completely disappearing, whereas the absorption at ca. 833 nm increased. The vanishing of the Soret band is consistent with the loss of macrocyclic aromaticity caused by oxidative cleavage of **1**. The NIR absorption at 982 nm may thus be linked to the initially formed BTO complexes, which are known to have markedly smaller electronic gaps than their parent metalloporphyrins^24^.

Subsequent evolution of NIR absorptions, leading to enhanced intensity at ca. 833 nm, is presumed to reflect hydration of the initially formed BTOs^20–23^. The resulting HBDO chromophore is characterized by a larger energy gap reflecting interrupted conjugation in the tetrapyrrole backbone. Since the equilibrated mixture displays both the 833 and 982 nm absorption bands, both chromophore types should be present in the final product. The conversion of **1** into **2** during the spectrophotometrically monitored irradiation is slower than in the corresponding NMR experiment, apparently because of the large difference in initial concentrations (0.01 mM vs. 3 mM, respectively). Water addition to BTOs could not be observed as a discrete step in the NMR experiment, indicating that it occurs faster than photocleavage under these conditions. Generally, relative rates of photocleavage and hydration may depend not only on the concentration of **1**, but also on the amount of residual water in the toluene solvent, irradiation conditions, and stirring. The formation of **2** was however reproducible at both concentrations in several repetitions of the cleavage experiments.

Dimeric complexes of linear tetrapyrroles are normally formed by homodimerization of two chemically equivalent units^21,25,26^. Dimer **2** is unique for the dual non-equivalence of its components, which differ not only in the cleavage site but also in the hydration state of the ligand. Additionally, subunit **A** contains a chirality center at the meso carbon 15, whereas subunit **B** is helically chiral. The assembly occurs stereospecifically, with the *R* enantiomer of **A** being combined with the *P* enantiomer of **B**. The interplay between point and helical chirality in **2** resembles the chiral induction observed for some chiral alkoxybilinones^27–29^, except that in the present case, the selection does not involve an external chirality source.

Interestingly, while porphyrin **1** quantitatively produces the heterodimer **2**, photooxidation of the corresponding zinc(II) tetra-NMI porphyrin^18^ under identical conditions produces a complex mixture of products, which could not be resolved using spectroscopic methods (Supplementary Fig. 16). The UV–vis–NIR spectrum of the latter mixture is consistent with the simultaneous presence of BTOs and HBDOs in comparable amounts. However, the low-field region of the ^1^H NMR spectrum contains several signals apparently corresponding to hydrogen-bonded OH groups of Zn^II^ HBDOs. Such diversity is surprising, given that the symmetrical tetra-NMI precursor should give rise to just one BTO and one HBDO product, and may indicate formation of higher-order supramolecular assemblies. The selective formation of **2** is thus specific to the di-NMI porphyrin complex **1** and highlights the importance of an accurate geometric match between the components.

### Computational analysis

**Fig. 5:**
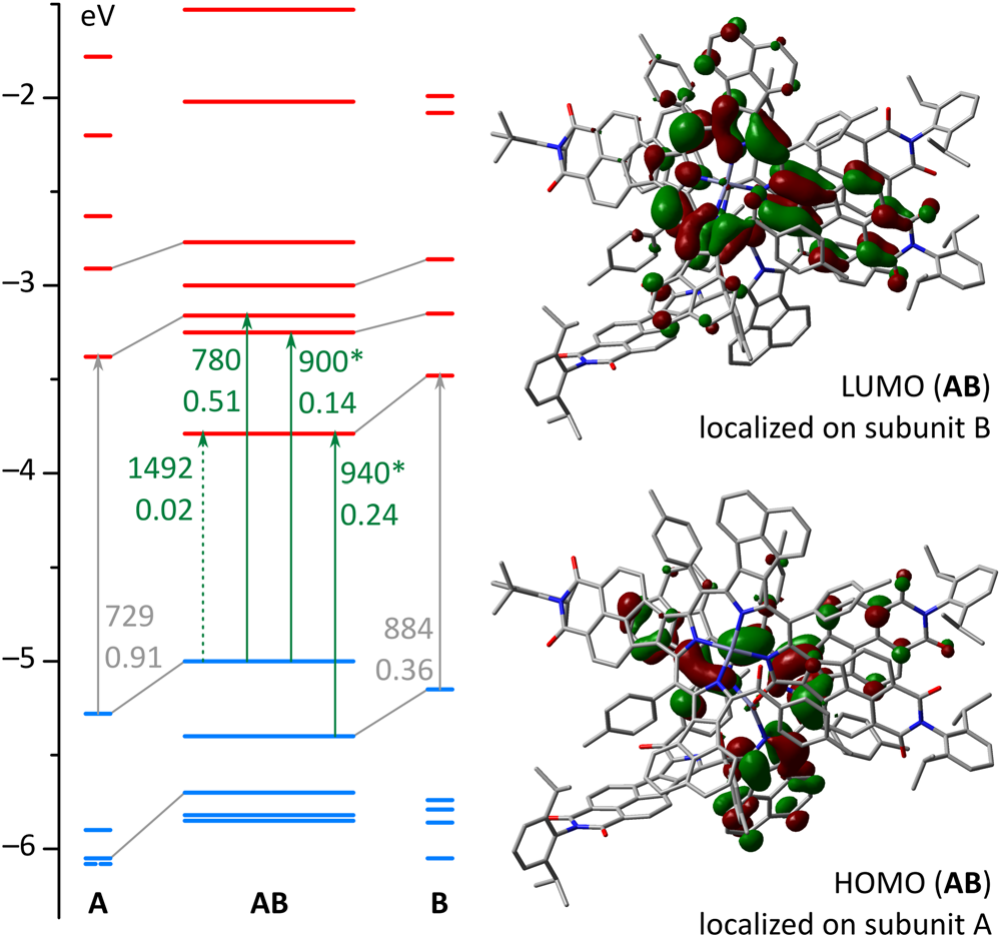
Molecular orbitals of **2** (=**AB**) and its constituent subunits **A** and B. (GD3BJ-B3LYP/6–31G(d,p)). Arrows and adjacent labels correspond to key electronic transitions, their wavelengths (nm), and oscillator strengths. Transitions marked with * contain contributions of other excitations (see text).

Dispersion-corrected density functional theory (DFT) produced a geometry of **2** very similar to that found in the solid state (Supplementary Fig. 18). Frontier Kohn–Sham molecular orbitals (MOs) of **2** show significant localization on individual chromophores and can be correlated with MOs of the isolated subunits **A** and **B** (Fig. 5 and Supplementary Fig. 17). Mixing of frontier MOs is typically insignificant (Supplementary Table 2), with subunit **A** contributing primarily to H–2 (84%), HOMO (96%), and L + 2 (96%), and subunit **B** contributing to H–1 (97%), LUMO (97%), and L + 1 (96%). In fact, HOMO and H–1 levels of the dimer correspond to nearly pure HOMOs of subunits **A** and **B**, respectively, whereas LUMO, L + 1 and L + 2 of **2** are equivalent respectively to LUMO (**B**), L + 1 (**B**), and LUMO (**A**). In the dimer, the frontier MO levels of **A** are systematically shifted to higher energies relative to the free monomer, whereas an opposite effect is observed for **B**. The calculated electronic gap for the dimer is consequently very small (1.25 eV), but the HOMO–LUMO electronic transition is predicted by time-dependent DFT to be near forbidden (1492 nm, *f* = 0.02) and indeed, it was not observed experimentally. Each of the two predicted transitions at 940 and 900 nm consists of two excitations: H–1 to LUMO, corresponding the HOMO–LUMO transition of **B**, and HOMO to L + 1, which has a charge-transfer character. In contrast, the transition at 780 nm corresponds to a pure HOMO to L + 2 excitation, i.e., the HOMO–LUMO transition of free subunit **A** (for complete TD-DFT data, see Supplementary Fig. 19–21 and Supplementary Tables 3–5).

**Fig. 6:**
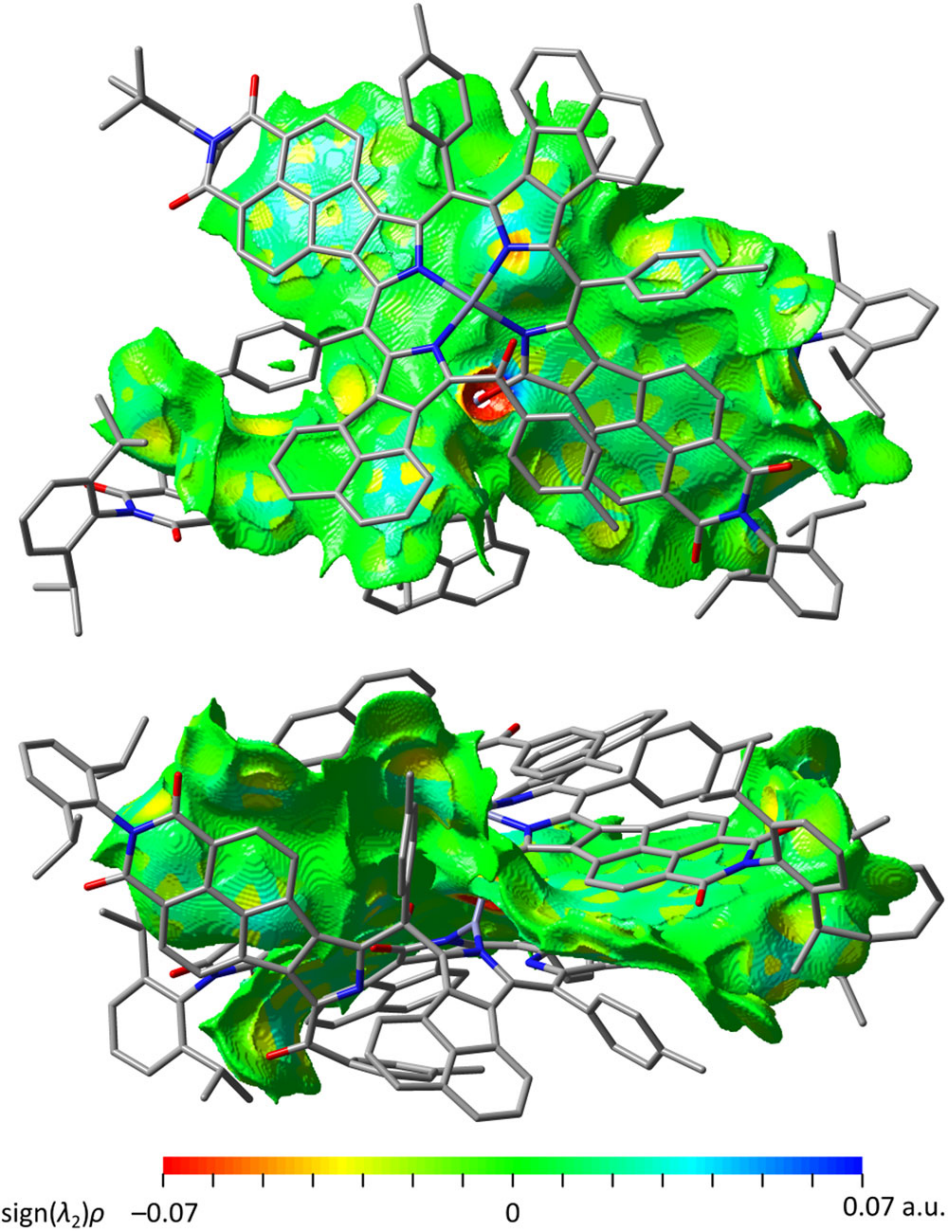
Inter-subunit non-covalent interactions in **2**. The calculation was performed using NCIPLOT4^31^ using a DFT-optimized geometry and promolecular electron density. The *s*(*ρ*) isosurface (1.0 a.u.) is mapped with sign(*λ*_2_)*ρ*, where *ρ*, *s*, and *λ*_2_ are respectively the electron density, its reduced gradient, and the second eigenvalue of the electron-density Hessian. Attractive and repulsive interactions correspond to *λ*_2_ < 0 and *λ*_2_ > 0, respectively^30^. Top and bottom panels show the top and side projections of **2**, respectively.

An analysis of non-covalent interactions performed for **2** using NCIPLOT4^30,31^ (Fig. 6) revealed a large contact surface between subunits **A** and **B**. The interaction area covers the entire tetrapyrrolic cores of both subunits, large sections of the acenaphthylene units, as well as some dipp and Tol substituents. The very significant contortion of the contact surface increases its area, contributing to further enhancement of the dispersive interaction component. A similarly large overlap of π surfaces was recently reported in a structurally cognate NMI-fused azacoronene nanosandwich, which was found to be principally stabilized by dispersion forces^32^.

**Fig. 7:**
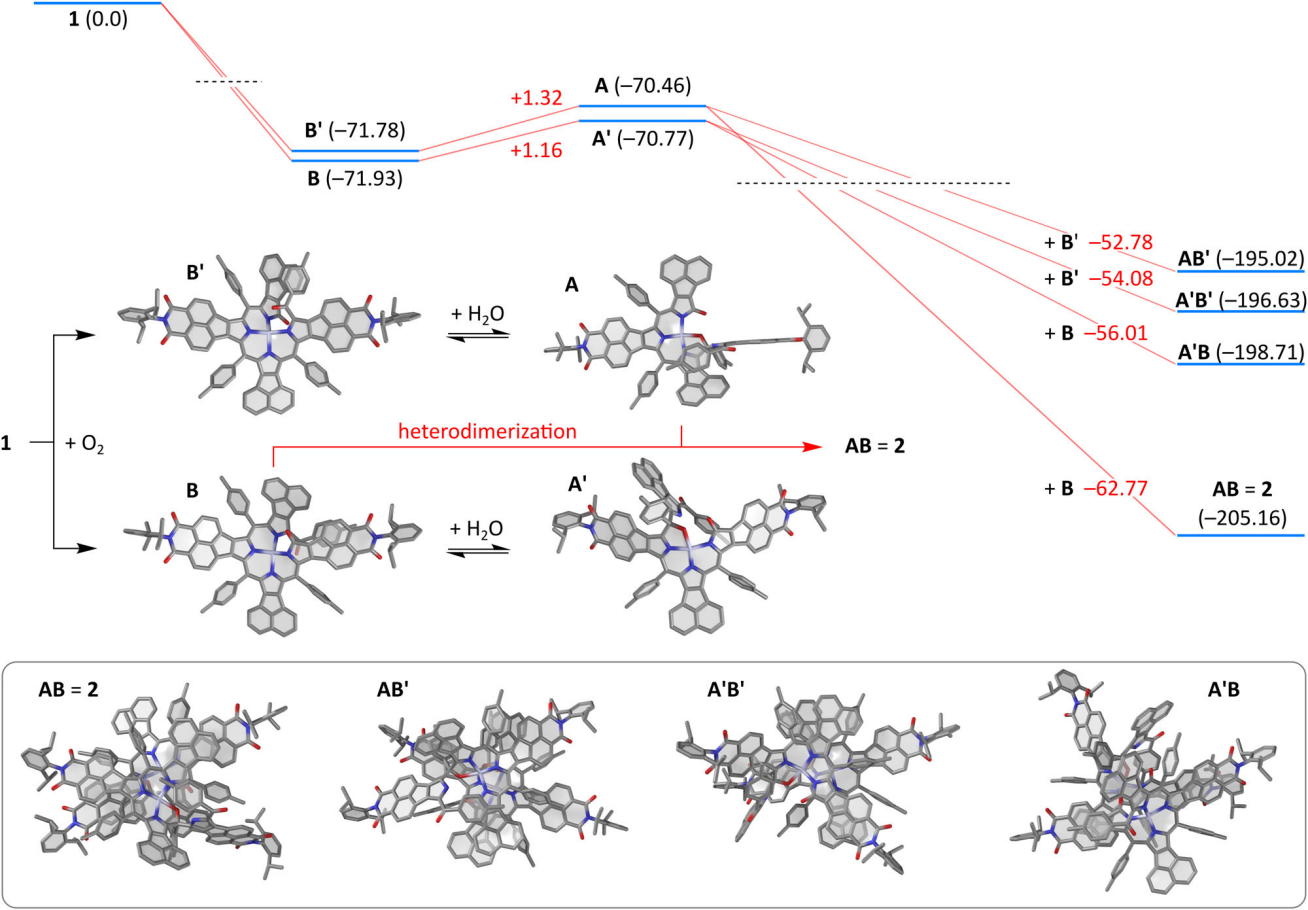
Thermodynamics of heterodimer formation explored using density functional theory. (SMD(toluene)/GD3BJ-B3LYP/6–31 G(d,p)). Δ*G*^298^ (in kcal/mol) are given relative to **1** (black) and the preceding intermediate (red). Vertical positions of energy bars are not to scale.

Density functional theory (DFT) simulations showed that the initial dioxygen-induced cleavage of **1** is highly exergonic, with Δ*G*^298^ of ca. −72 kcal/mol (Fig. 7), with the two regioisomeric cleavage products **B** and **B’** having nearly identical energies. While the relative kinetics of formation of these two intermediates cannot be directly inferred from their relative energies, the small difference of Δ*G* values indicates that the different positioning of the remote imide groups with respect to the cleavage site has an only minor effect on the overall energetics of the BTO system. Oxidative cleavage of porphyrins is known to be controlled by steric accessibility of C–C bonds rather than by electronic effects^33^. The latter characteristic explains why compound **1**, in spite of its mixed donor–acceptor structure, produces equal amounts of **B** and **B’**. Interestingly, water addition to **B** and **B’**, yielding respectively zinc(II) 15-hydroxybiladien-*ab*-ones **A’** and **A**, is predicted to produce a very small change of Δ*G*^298^ of +1.16 and +1.32 kcal/mol respectively. These values imply that the hydration step should indeed be reversible^20^ and that there should be no intrinsic preference for either **A** or **A’**. If addition of water to **B** and **B’** is indeed endergonic, as predicted by DFT, the driving force for hydration must be provided be the subsequent dimerization step, and the selectivity toward **A** should result from the preferred formation of one type of heterodimer.

To test this hypothesis, DFT structures of the four possible heterodimers, **AB** (=**2**), **A’B**, **AB’**, and **A’B’**, were optimized, starting with the XRD geometry of **2**, in which the imide subunits were appropriately transposed (Fig. 7). In this way, the relative orientation of BTO (**B**/**B’**) and HBDO (**A**/**A’**) cores was approximately retained in all dimers. The native dimer **AB** yielded a large negative dimerization Δ*G*^298^ of –62.77 kcal/mol, lower by nearly 7 kcal/mol than the next most stable structure (**A’B**). Dimerization energies of these dimers reflect the combined effect of the Zn–O bond formation and dispersion interactions between the monomer π surfaces. The latter contribution was shown to be very large in a recently reported σ-dimer containing NMI-pyrrole subunits^32^. These data indicate that (a) the energetic effect of heterodimerization is sufficient to induce hydration of bilatrien-*abc*-ones and (b) the selection of the **AB** dimer may indeed be justified by its unique thermodynamic stability.

In the above analysis, the theoretical Δ*G*^298^ of dimerization is predicted to have a very large negative value, and is not expected to reflect the actual association energetics of **2**. The computed Δ*G* estimate is affected by the combination of errors introduced by the DFT functional, basis set, empirical dispersion, PCM solvation, and thermochemistry calculations. In particular dispersion-related deviations may exceed 5 meV per non-H atom^34,35^, which may correspond to a dimerization energy error of over 25 kcal/mol for a system as large as **2**. The cleavage and hydration steps, which do not involve large changes in dispersion interactions, should be affected by these inaccuracies to a much smaller extent. Since the errors discussed above are systematic, relative dimerization energies may be expected to be sufficiently reliable to support the preferred formation of the **AB** dimer relative to its isomers.

### Dissociation and reconstitution of 2

**Fig. 8:**
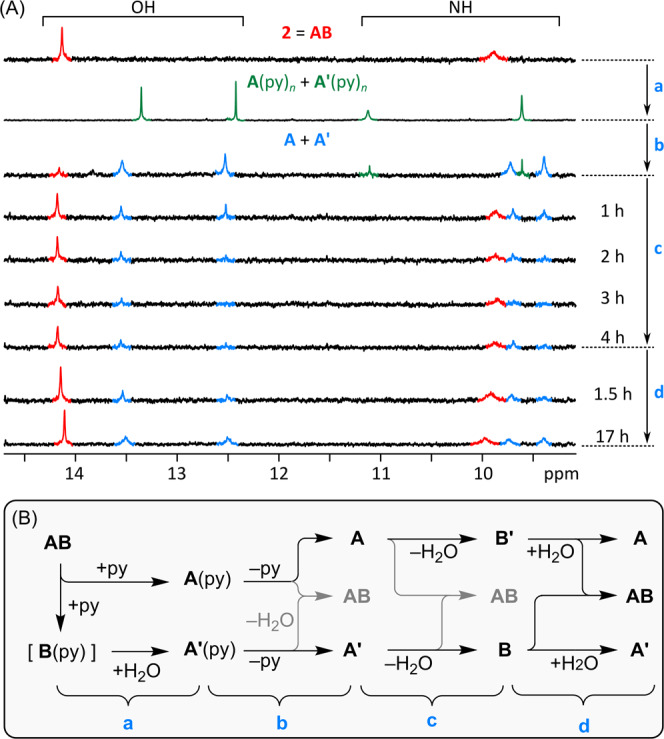
Cleavage and reconstitution of **2** (= **AB**). **A** changes observed using ^1^H NMR spectroscopy (toluene-*d*_8_, room temperature). **B** Proposed reaction sequence. Reagents and conditions (blue labels): (a) pyridine-*d*_5_ (ca. 180 equiv); (b) 1. solvent evaporation (5 cycles, fresh toluene-*d*_8_ added before each cycle), 2. evacuation (15 h), 3. toluene-*d*_8_, 4 Å molecular sieves (powder); (c) heating (90 °C), 4 h; (d) solvent evaporation, fresh toluene-*d*_8_ added.

To test if the dimer might be susceptible to dissociation in the presence of strong ligands, we treated a toluene-*d*_8_ solution of **2** with ca. 180 equiv of pyridine-*d*_5_. An equimolar mixture of two products was formed in a near stoichiometric amount, as observed using in-situ ^1^H NMR spectroscopy (Fig. 8, step **a**). By addition of a smaller amount of pyridine-*d*_5_ (Supplementary Fig. 15), it was possible to achieve partial conversion, with some **2** still present in the mixture. The two products and the remaining **2** showed no observable exchange in the ROESY spectrum, whereas DOSY data indicated that the new species have comparable molecular weights, smaller than the MW of **2**. Each product featured a narrow ^1^H NMR resonance in the 12–14 ppm range, assignable to the hydrogen-bonded OH group of zinc(II) 15-hydroxybiladien-*ab*-one, and a broader signal at 9–11 ppm, corresponding to NH proton of the pendant pyrrole. The products could thus be identified as the isomeric monomers **A**(py)_*n*_ and **A’**(py)_*n*_ (*n* = 1 or 2, cf. Figure 7). The formation of **A’** indicated that hydration of the intermediate **B** occurred immediately after cleavage of the dimer.

To reconstitute the **AB** dimer, it was necessary to remove not only pyridine-*d*_5_ but also the traces of dissolved water (Fig. 8, step **b**). After replacing the solvent with pure toluene-*d*_8_ and addition of powdered 4 Å molecular sieves, the mixture obtained contained predominantly two monomeric HBDO species, most likely **A** and **A’**, which were indeed accompanied by a small amount of reconstituted dimer. The sample was then heated at 90 °C to enhance the kinetics of dehydration (step **c**). This operation resulted in near-complete consumption of **A** and **A’**, which were partly converted back into **AB**. However, additional signals observed in the aromatic region of the ^1^H NMR spectrum indicated formation of other species, tentatively assigned the monomeric dehydration products **B** and **B’**. To validate this assumption, we removed the molecular sieves and replaced the solvent with non-dried toluene-*d*_8_. This step (**d** in Fig. 8) resulted in further reconstitution of the dimer and reappearance of the hydrated products **A** and **A’**, with the final **AB**:**A**:**A’** molar ratio of ca. 1:0.5:0.5.

In the above sequence of experiments, the formation of **2** appears to be selective, i.e., no other heterodimers (**A’B**, **AB’**, or **A’B’**) are formed. However, the assembly process depends not only on thermodynamics of hydration and apical coordination, which are controlled by concentrations of water and pyridine, respectively, but also on relative dehydration and hydration kinetics for the **A**/**B’** and **A’**/**B** pairs. **AB** forms spontaneously if **A** and **B** are simultaneously present in solution, and once formed, it is resistant to dehydration by molecular sieves. However, since dehydration performed in step **c** is apparently unselective, some **A** is converted into **B’**, leaving the corresponding amount of **B** without the dimerization partner. Likewise, during addition of water performed in step **d**, some **A’** is produced in addition to **A**, depleting the amount of **B** available for heterodimerization. Such kinetic effects may explain incomplete reconstitution of the **AB** dimer. However, the selectivities observed in steps **c** and **d** may also be affected by the presence of residual pyridine-*d*_5_ which could potentially remain in the sample after step **b**.

## Discussion

The completive self-sorting process described herein provides a glimpse of structural complexity that can be generated from donor–acceptor oligopyrroles by combining covalent reactivity with supramolecular interactions. The high-yielding formation of the heterodimer under photooxidation conditions is made possible by a favorable combination of thermodynamic and kinetic factors. Dispersion interactions provide the driving force for the self-assembly and are also responsible for the observed selectivity of hydration. The resulting heterodimer, derived from a single metalloporphyrin, contains two chromophores with different conjugation lengths, leading to a structure with partial donor–acceptor character and non-negligible interaction between the subunits. This feature implies prospective use of photochemical chromophore diversification for in-situ synthesis of supramolecular electron- and energy-transfer systems.

## Methods

### Materials

Toluene was dried using a commercial solvent purification system and all other solvents and reagents were used as received. Zinc(II) porphyrin **1**^17^ and its tetra-NMI analogue^18^ were synthesized as shown in the Supplementary Information. NMR spectral assignments were made using two-dimensional ^1^H and ^13^C NMR spectroscopy.

### Irradiation experiments

A 5 mL flask containing zinc(II) porphyrin **1** (3 mg, 1.8 μmol) and toluene-*d*_8_ (0.6 mL) was rotated on a rotary evaporator (200 rpm, room temperature and ambient pressure) and irradiated using a 4000 K consumer LED source. After 4 h, the quantitative formation of heterodimer **2** was confirmed by NMR analyses. Zinc(II) tetra-NMI porphyrin was irradiated similarly.

The progress of the photocleavage was monitored spectrophotometrically using a Varian Cary 60 spectrophotometer. In a typical experiment, the zinc(II) porphyrin (3 mL of a 0.01 mM solution in toluene) was placed in a 10 mm path quartz cell equipped with a stir bar, and the spectra were recorded in air at room temperature. After the first spectrum was recorded, the sample was irradiated with the LED source for 12 h until no further change of the electronic spectrum was observed.

### Heterodimer (2)

^**1**^**H NMR** (500 MHz, toluene-*d*_8_, 300 K): δ 14.34 (1H, s), 10.12 (1H, b), 9.06 (1H, b), 8.99 (1H, d^3^, *J* = 7.1 Hz), 8.57 (1H, b), 8.49 (1H, d^3^, *J* = 7.3 Hz), 8.31 (1H, m), 8.27 (1H, d^3^, *J* = 7.0 Hz), 8.19 (2H, d^3^, *J* = 7.1 Hz), 8.11 (2H, t^3^, *J* = 7.6 Hz), 7.73 (4H, b), 7.66 (2H, d^3^, *J* = 7.6 Hz), 7.60 (4H, d ^3^, *J* = 7.3 Hz), 7.53 (4H, m), 7.47 (8H, m), 7.31 (24H, m), 6.91 (4H, m), 6.85 (3H, m), 6.78 (3H, m), 6.56 (2H, d ^3^, *J* = 7.0 Hz), 6.49 (1H, d ^3^, *J* = 7.1 Hz), 6.44 (2H, b), 6.36 (1H, b), 6.29 (1H, m), 6.24 (1H, m), 5.30 (1H, d^3^, *J* = 7.3 Hz), 5.10 (1H, d^3^, *J* = 7.4 Hz), 5.05 (2H, m), 4.99 (1H, d^3^, *J* = 7.3 Hz), 4.94 (2H, m), 4.27 (1H, d^3^, *J* = 7.3 Hz), 3.87 (1H, d^3^, *J* = 7.3 Hz), 3.80 (1H, d^3^, *J* = 7.3 Hz), 3.35 (2H, m), 3.17 (3H, m), 2.88 (1H, m), 2.72 (2H, m), 2.45 (3H, s), 2.23 (3H, s), 2.19 (3H, s), 2.08 (3H, s), 1.94 (3H, s), 1.71 (3H, s), 1.66 (3H, d^3^, *J* = 6.6 Hz), 1.60 (9H, m), 1.53 (6H, m), 1.47 (6H, m), 1.39 (9H, m), 1.34 (3H, m), 1.21 (3H, m), 1.15 (3H, m), 1.10 (9H, m), 0.98 (3H, d^3^, *J* = 6.8 Hz). **HRMS** (ESI–TOF): *m*/*z*: [A–H]^–^ Calcd for C_116_H_83_N_6_O_7_Zn: 1737.5669; Found 1737.5257, (MALDI–TOF): *m*/*z*: [B + H]^+^ Calcd for C_116_H_82_N_6_O_6_Zn: 1721.5720; Found 1721.5818.

## Supplementary information

Supplementary Information

Description of Additional Supplementary Files

Supplementary Data File 1 cartesian coordinates

## Data Availability

The data supporting the findings of this study are included within the Article and its Supplementary Information files. CCDC 2019726 contains the supplementary crystallographic data for this paper. Cartesian coordinates can be found in Supplementary Data 1.
