# Supplementary material for: Synthesis of a donor–acceptor heterodimer via trifunctional completive self-sorting
Source: Nat Commun. 2022 Jun 9;13:3204. doi: 10.1038/s41467-022-30859-7 (PMC9184498; doi:10.1038/s41467-022-30859-7)
Supplement: Supplementary file 1 — Supplementary Information [file 41467_2022_30859_MOESM1_ESM.pdf]

# Synthesis of a Donor–Acceptor Heterodimer via Trifunctional Completive Self-Sorting

**Supplementary Information**

by Sunit Kumar, Yogesh Kumar Maurya, Tadeusz Lis, and Marcin Stępień *

## Table of Contents

Supplementary Methods ........................................................................................................2

Supplementary Figures ........................................................................................................3

Supplementary Tables ........................................................................................................19

References ........................................................................................................24

# Supplementary Methods

**Spectroscopy.** $^1H$ NMR spectra were recorded on high-field spectrometers ($^1H$ frequency 500.13 or 600.13 MHz), equipped with broadband inverse or conventional gradient probe heads. Spectra were referenced to the residual solvent signals (chloroform-*d* 7.24 ppm; toluene-$d_8$ 2.09 ppm). Two-dimensional NMR spectra were recorded with 2048 data points in the $t_2$ domain and up to 2048 points in the $t_1$ domain, with a 1.5 s recovery delay. All 2D spectra were recorded with gradient selection, with the exception of ROESY and for this spinlock time was 300 ms. High-resolution mass spectra were recorded using MALDI ionization in the positive mode. MALDI-ToFMS was performed with a Bruker ultrafleXtreme in the positive-ion mode, using trans-2-[3-(4-tertbutylphenyl)-2-methyl-2-propenylidene]malonitrile (DCTB) as the matrix. The matrix-to-analyte ratio was 40:1.

**X-ray crystallography.** X-ray quality crystals of heterodimer **2** were grown by slow evaporation of toluene solvent. Diffraction measurements were performed on an Xcalibur Gemini Ultra diffractometer, equipped with a Ruby CCD camera, with graphitemonochromatized Cu Kα radiation. The data were collected at 100 K, corrected for Lorenz and polarization effects. Data collection, cell refinement, data reduction and analysis were carried out with the CrysAlisPro software (Rigaku OD, 2018). An analytical absorption correction was applied. All structures were solved by direct methods with the SHELXS-97 program and refined using SHELXL-2013[1] with anisotropic thermal parameters for non-H atoms. Disordered positions were treated with a combination of anisotropic and isotropic refinement, and restrained using DFIX and FLAT restraints. In the final refinement cycles, all H atoms were treated as riding atoms in geometrically optimized positions. CCDC Mercury and CrystalExplorer[2,3] were used for visualization of solvent-accessible surfaces. CCDC 2019726 contains the supplementary crystallographic data for this paper. These data can be obtained free of charge from the Cambridge Crystallographic Data Centre via www.ccdc.cam.ac.uk/data_request/cif.

**Computational methods.** Density functional theory (DFT) calculations were performed using Gaussian 16. DFT geometry optimizations were carried out in unconstrained $C_1$ symmetry, using molecular mechanics or semi-empirical models as starting geometries. DFT geometries were refined to meet standard convergence criteria, and the existence of a stationary point was verified by a normal mode frequency calculation. Geometry optimizations, frequency calculations, and thermochemistry calculations were performed using the hybrid functional B3LYP[4–6] combined with the 6-31G(d,p) basis set and the and Grimme's GD3BJ dispersion correction.[7] In the calculations of absorption spectra, up to 50 electronic transitions were calculated by means of time-dependent DFT (TD-DFT), using the above level of theory, SMD solvation model[8] (standard toluene parameters), and the Tamm–Dancoff approximation (TDA).[9] Non-covalent interactions in **2** were analyzed using NCIPLOT4, using a DFT-optimized geometry and promolecular electron density.[10,11]

# Supplementary Figures

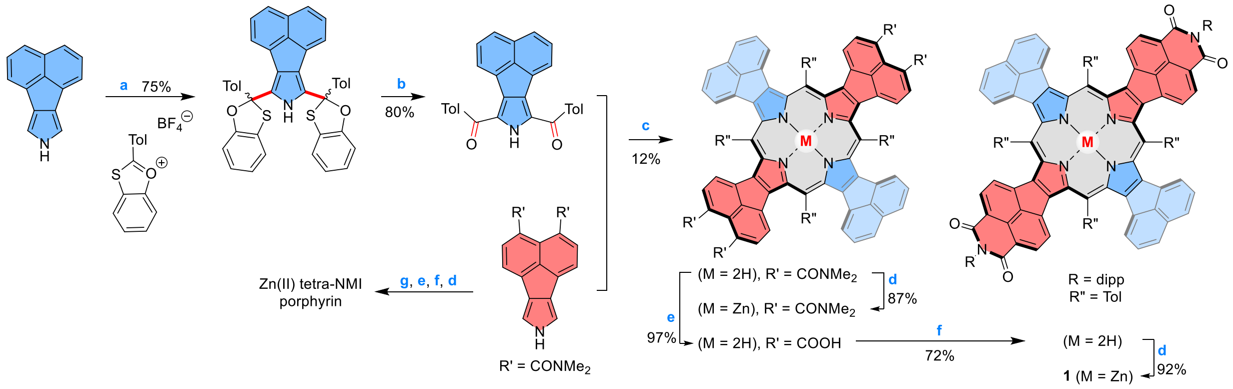


**Supplementary Figure 1. Synthesis of 1 and Zn(II) tetra-NMI porphyrin.**[12,13] Reagents and conditions: (a) pyridine, $CH_3CN/CHCl_3$ (1:1 v/v), 1 h; (b) HgO, $HBF_4$, THF, 5 h; (c) (i) **6**, $NaBH_4$, THF/MeOH (3:1 v/v), 2 h, (ii) **7** (**4**, for **3a**-$H_2$), *p*-TSA, $CHCl_3$/MeOH (100:1 v/v), 1 h, (iii) DDQ, 2 h; (d) $Zn(OAc)_2{\cdot}2H_2O$, $CHCl_3$/MeOH (3:1 v/v); (e) HCl, reflux, 24 h; (f) 2,6-diisopropylaniline, acetic acid, 20 h, reflux. (g) *p*-tolylaldehyde, $CHCl_3$, *p*-toluenesulfonic acid, 1 h, then DDQ. Tol = *p*-tolyl, dipp = 2,6-diisopropylphenyl.

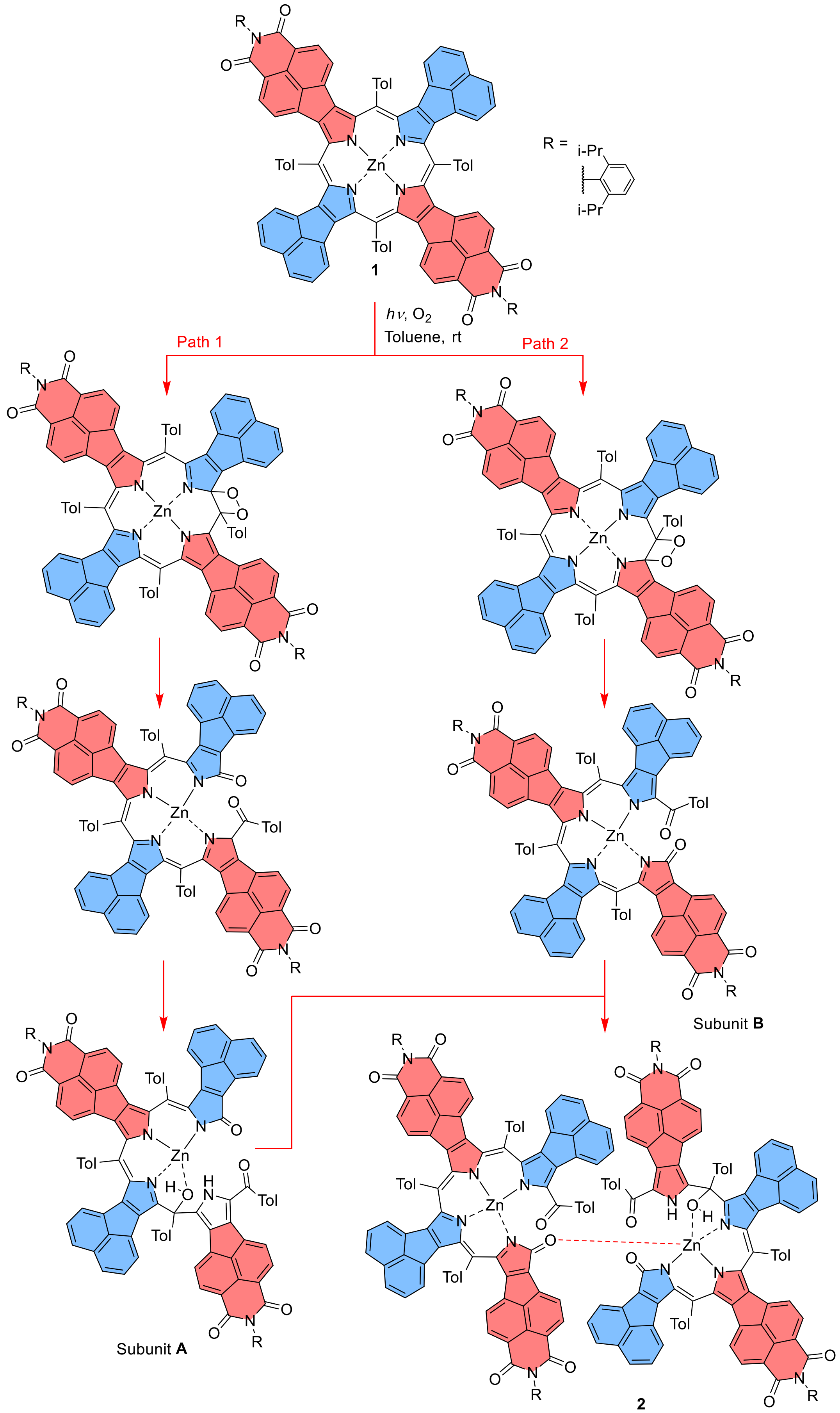


**Supplementary Figure 2. Proposed reaction mechanism for the formation of heterodimer 2.**

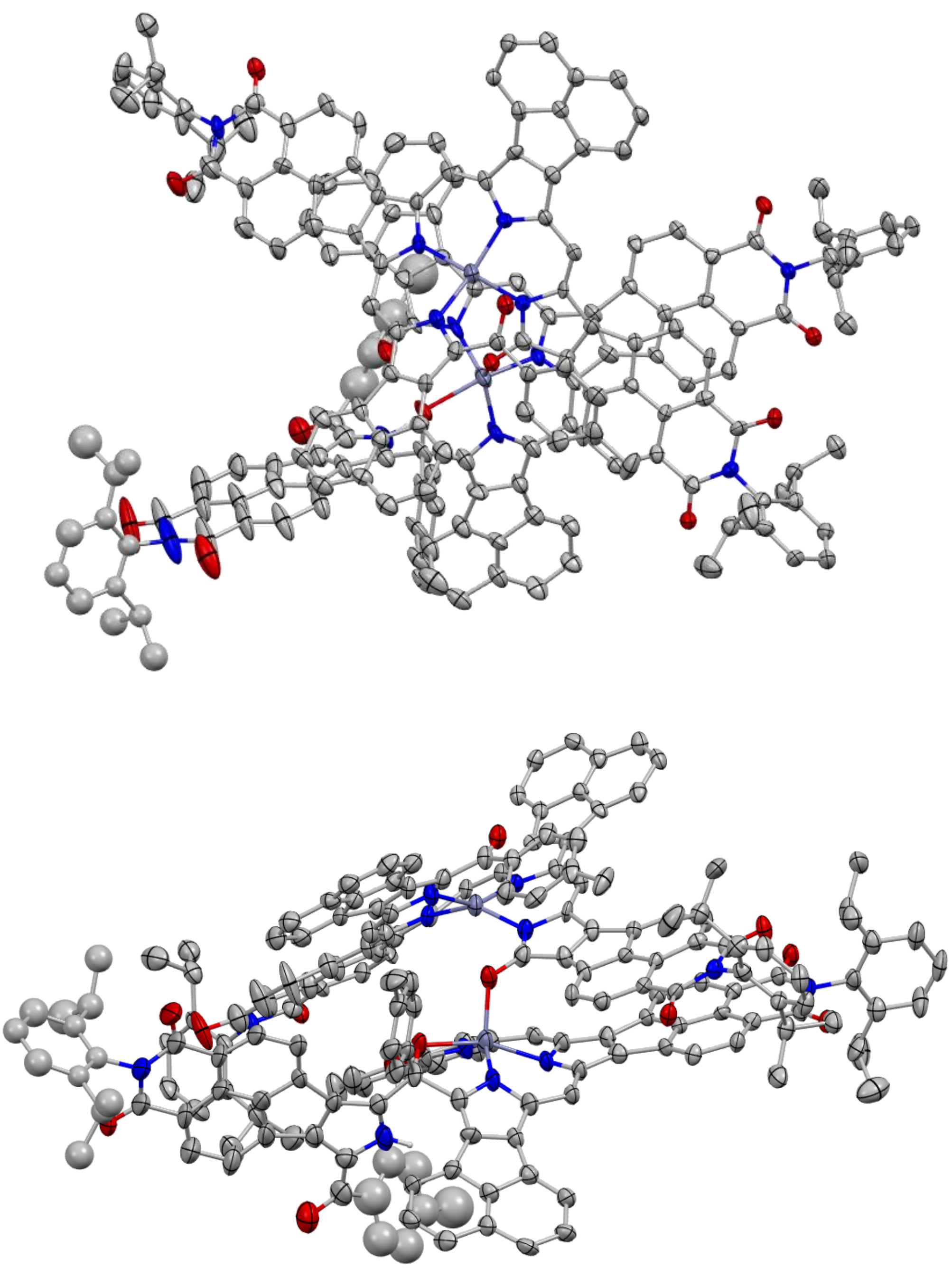

**Supplementary Figure 3. Molecular structure of dimer 2·8.2 $C_7D_8$ (top and side view).** Hydrogen atoms, solvent molecules and *meso*-tolyl rings are omitted for clarity .

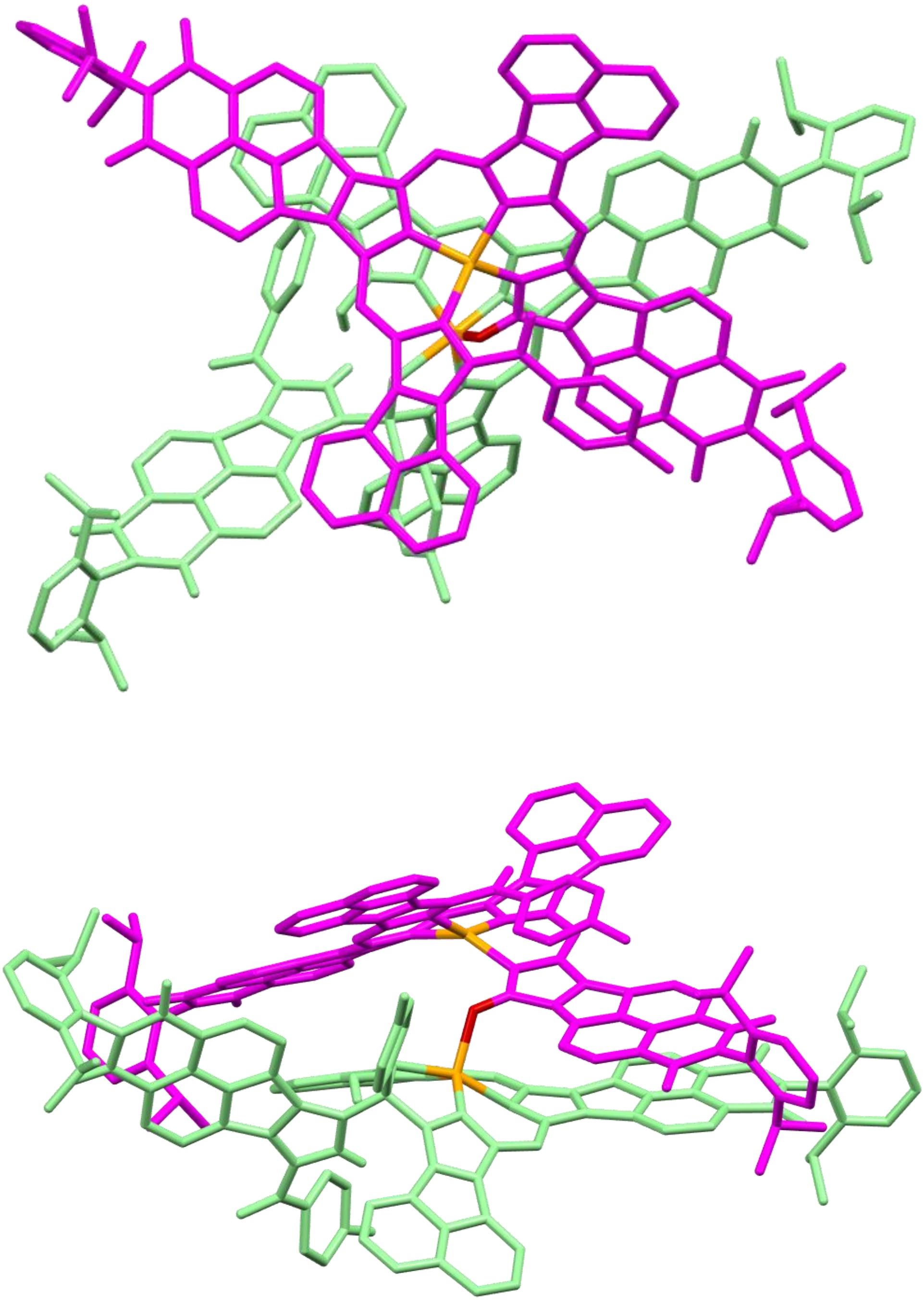

**Supplementary Figure 4. Stick representation of dimer 2·8.2 $C_7D_8$ (top and side view).** The BTO and HBDO subunits are shown in green and purple, respectively. Hydrogen atoms, solvent molecule and *meso*-tolyl ring are omitted for clarity.

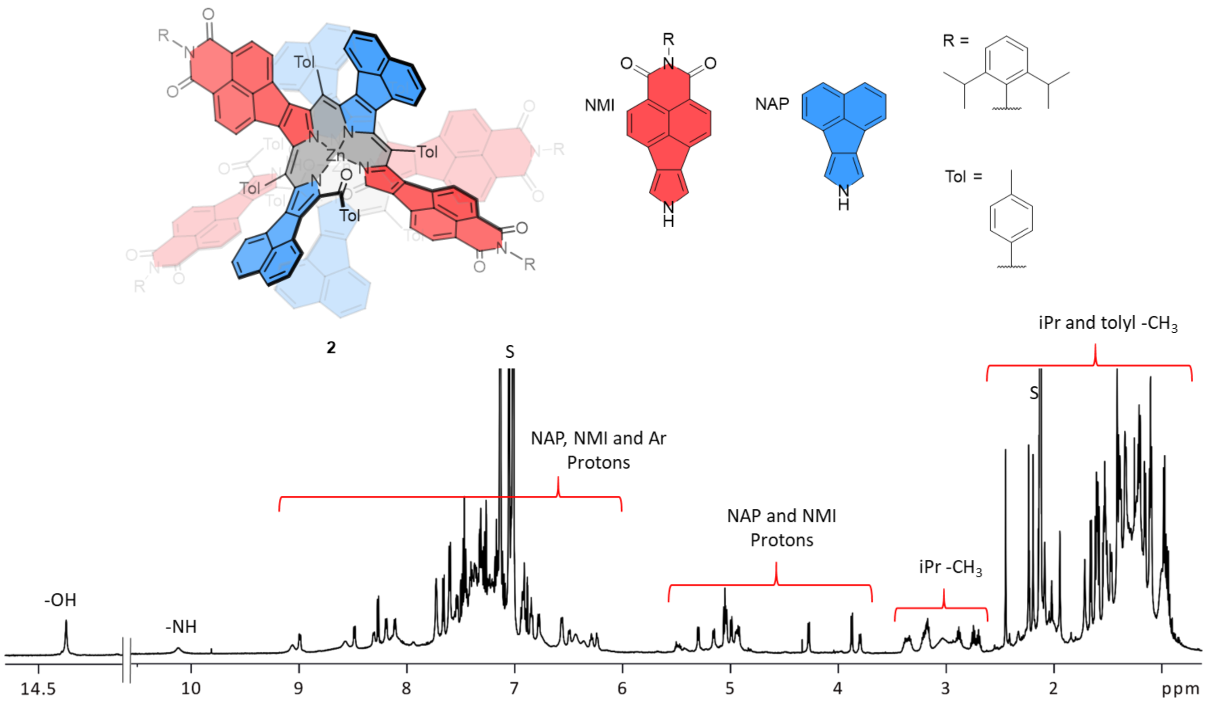


**Supplementary Figure 5. $^1$H NMR spectrum of heterodimer 2.** The spectrum was recorded in toluene-$d_8$ at room temperature.

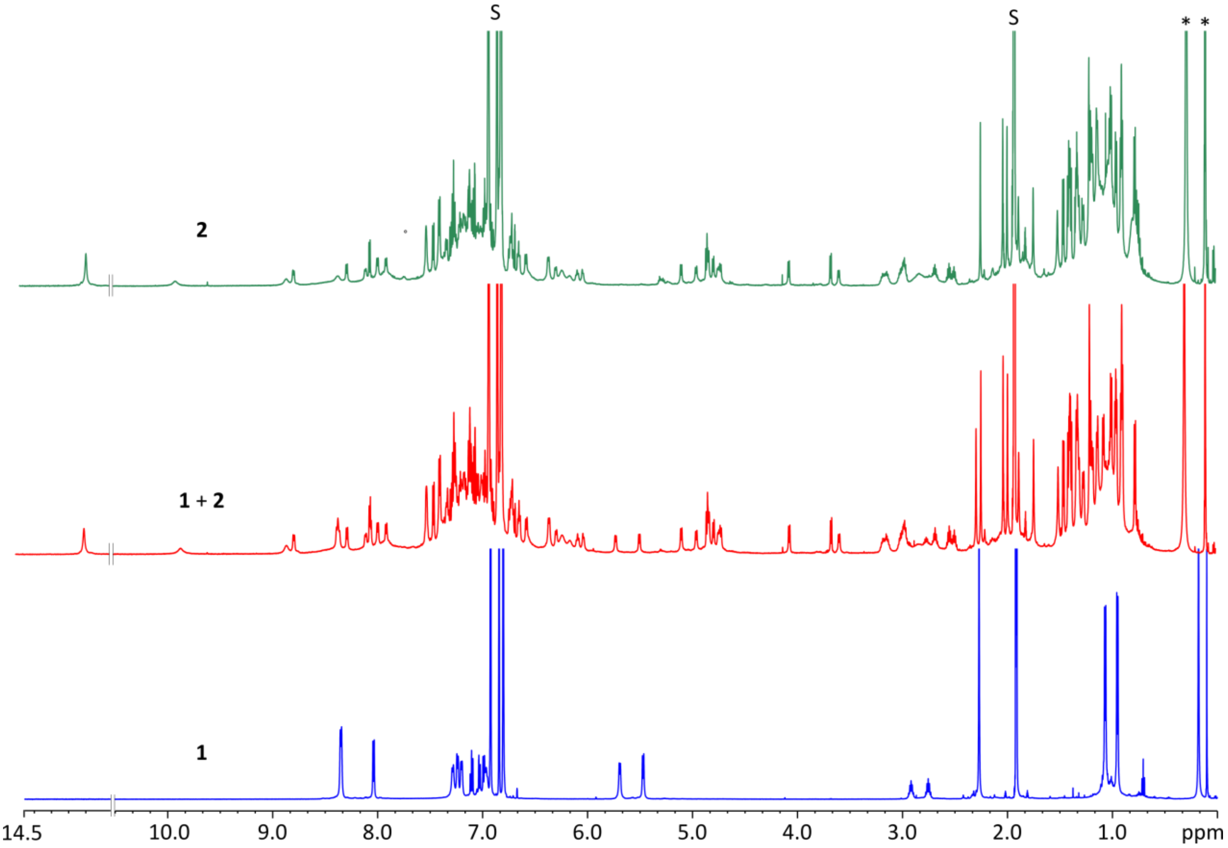


**Supplementary Figure 6. Comparison of $^1$H NMR spectra** of zinc(II) diNMI porphyrin **1** (blue), partly converted heterodimer (**1** + **2**) and heterodimer **2** (green) recorded in toluene-$d_8$ at room temperature.

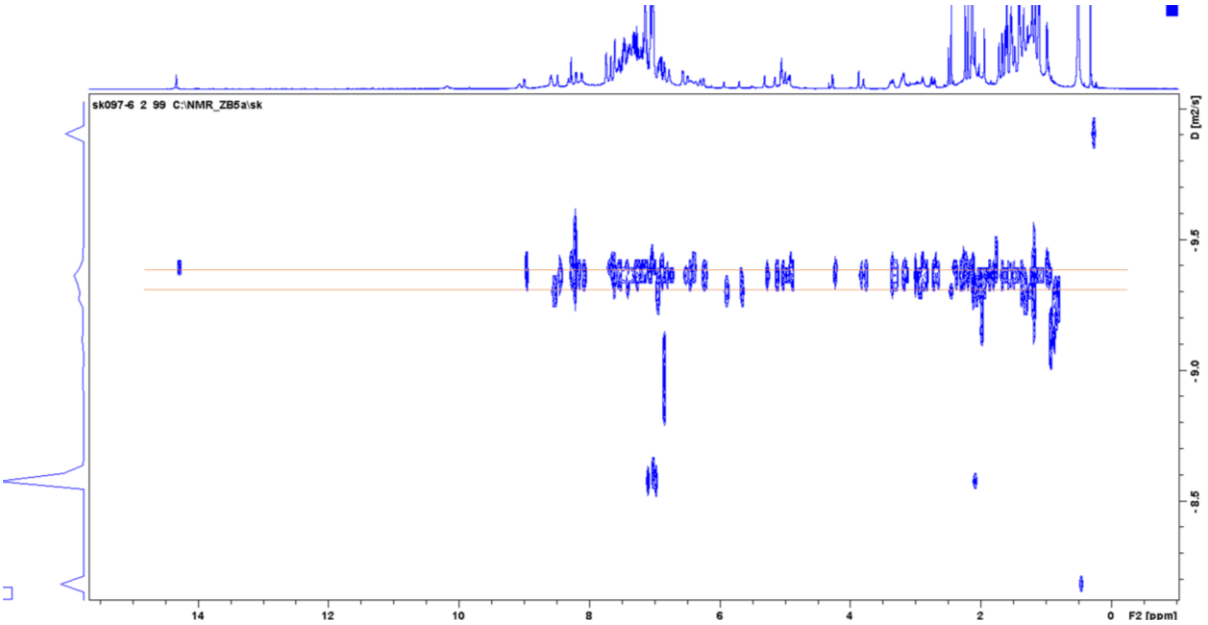


**Supplementary Figure 7. Partial 2D DOSY NMR spectrum** of the partly converted heterodimer (**1** + **2**) recorded in toluene-$d_8$ at room temperature .

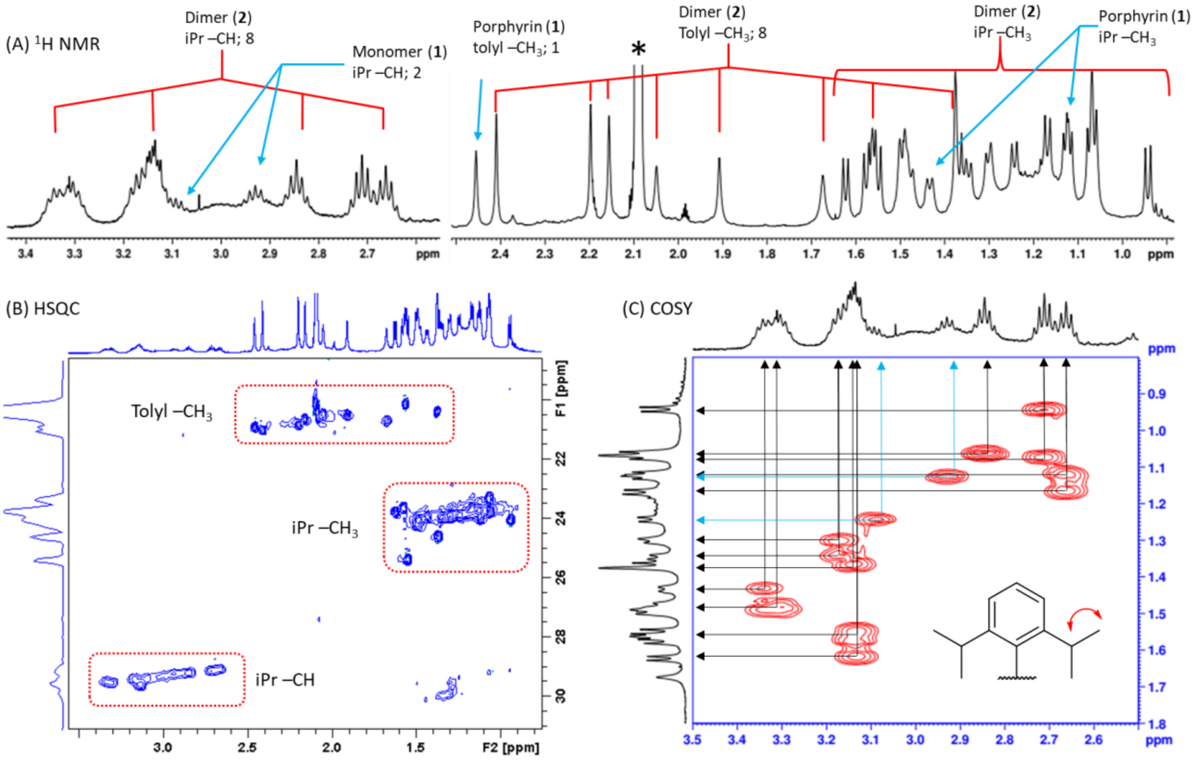


**Supplementary Figure 8. Correlation NMR spectroscopy for 1 and 2.** (A) Partial assingnment of $^{1}$H NMR spectrum of the partly converted heterodimer (**1** + **2**) recorded in toluene-$d_8$ at room temperature based on COSY, HSQC and ROESY correlations. (B) Partial HSQC correlations and (C) COSY correlation; black lines are corresponds to heterodimer **2** and skyblue lines corresponds to zinc(II) di-NMI porphyrin **1**.

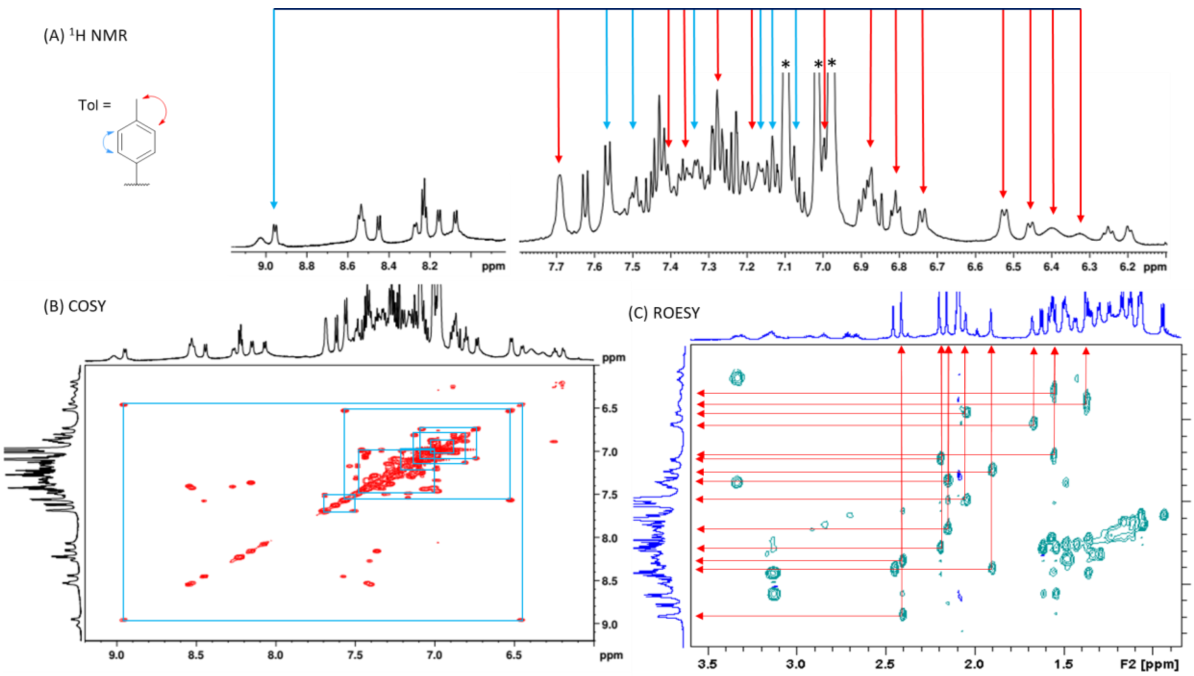


**Supplementary Figure 9. Correlation NMR spectroscopy for 1 and 2.** (A) Partial assignment of $^1$H NMR spectrum of the partly converted heterodimer (**1** + **2**) recorded in toluene-$d_8$ at room temperature based on COSY (skyblue line) and ROESY (red line) correlations. (B) Partial COSY correlations spectrum and (C) Partial ROESY correlation spectrum.

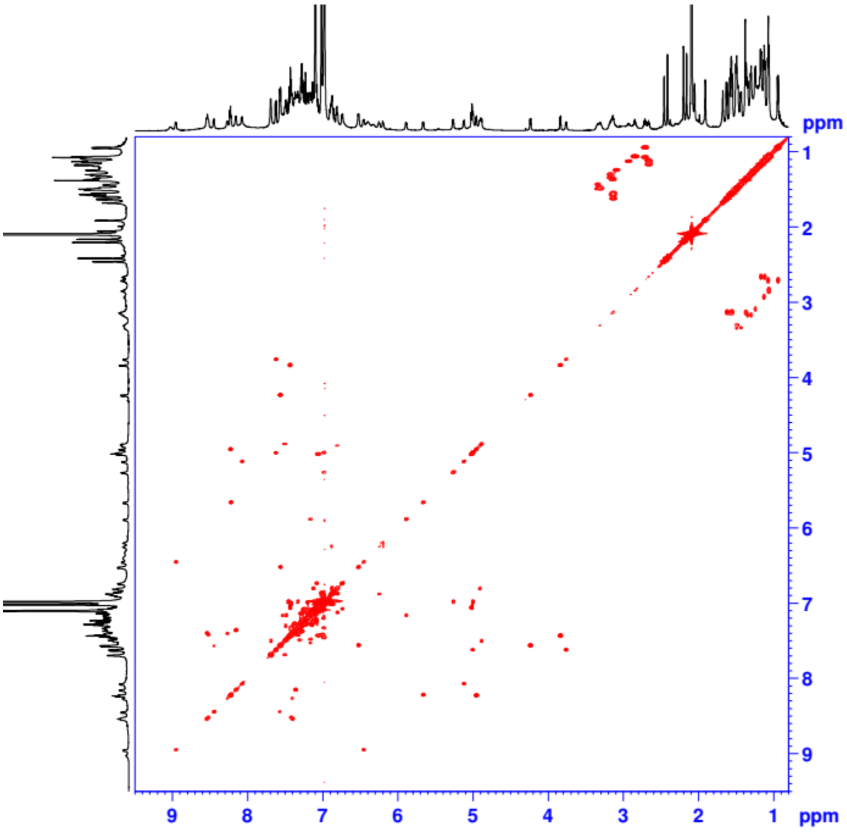

**Supplementary Figure 10. Correlation NMR spectroscopy for 1 and 2.** Partial $^1$H-$^1$H COSY spectrum of partly converted heterodimer (**1** + **2**) recorded in toluene-$d_8$ at room temperature.

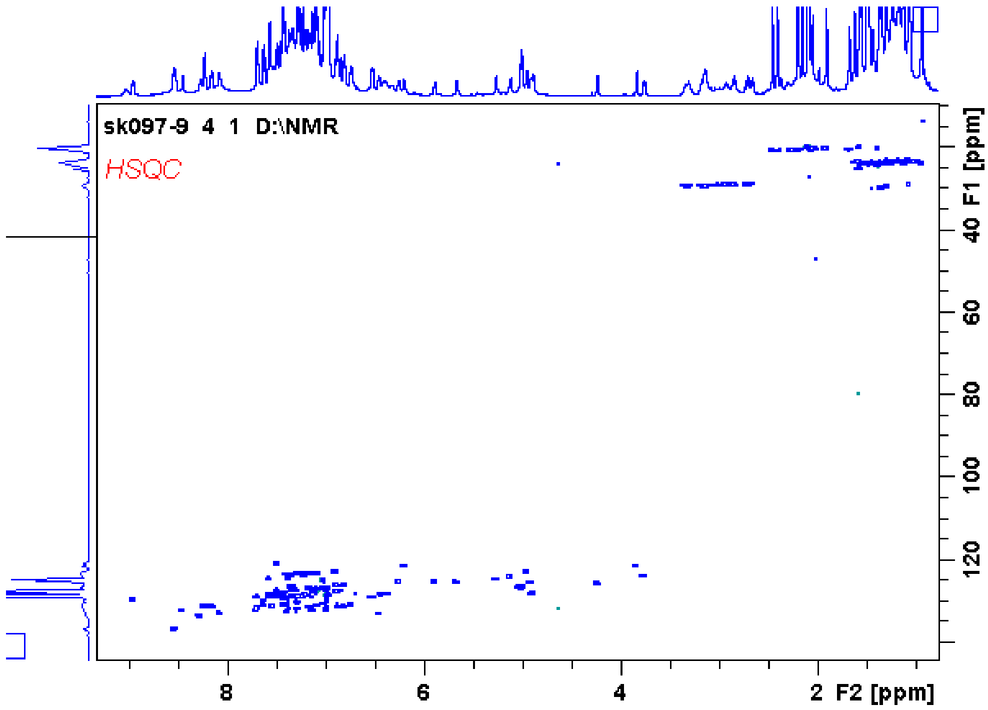


**Supplementary Figure 11. Correlation NMR spectroscopy for 1 and 2.** Partial HSQC spectrum of partly converted heterodimer (**1** + **2**) recorded in toluene-$d_8$ at room temperature.

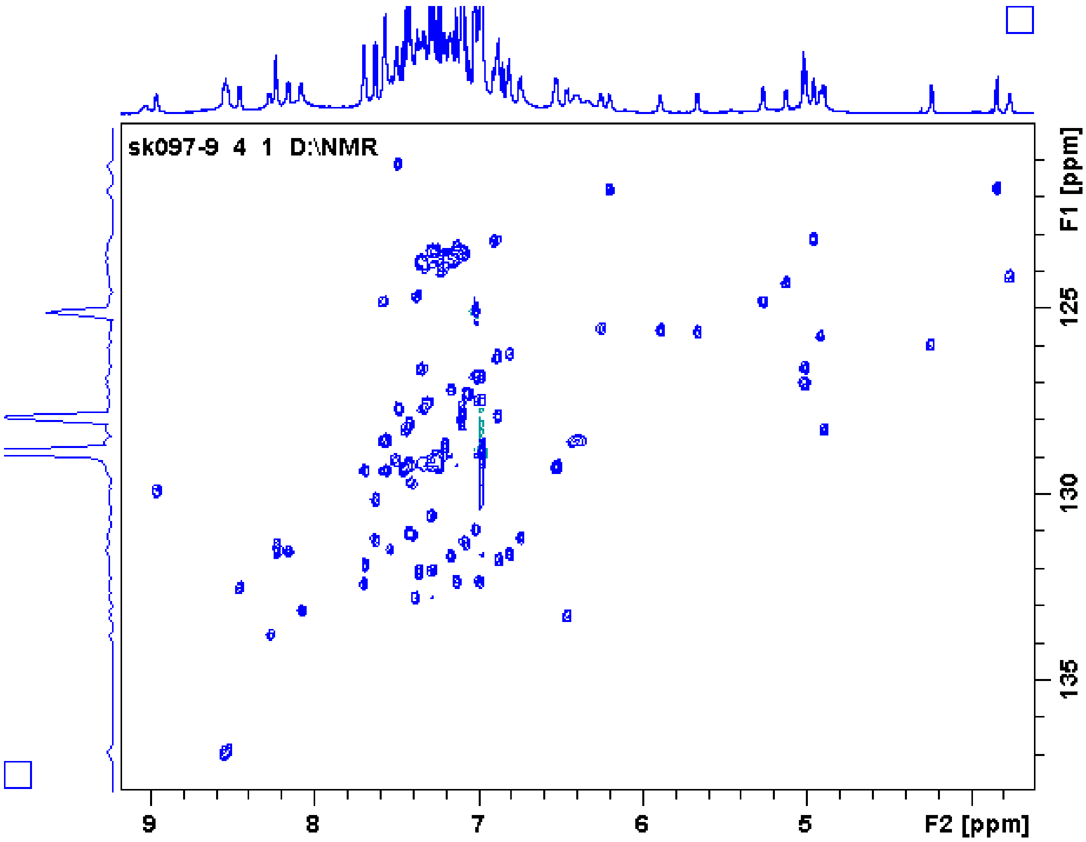


**Supplementary Figure 12. Correlation NMR spectroscopy for 1 and 2.** Partial HSQC spectrum of partly converted heterodimer (**1** + **2**) recorded in toluene-$d_8$ at room temperature.

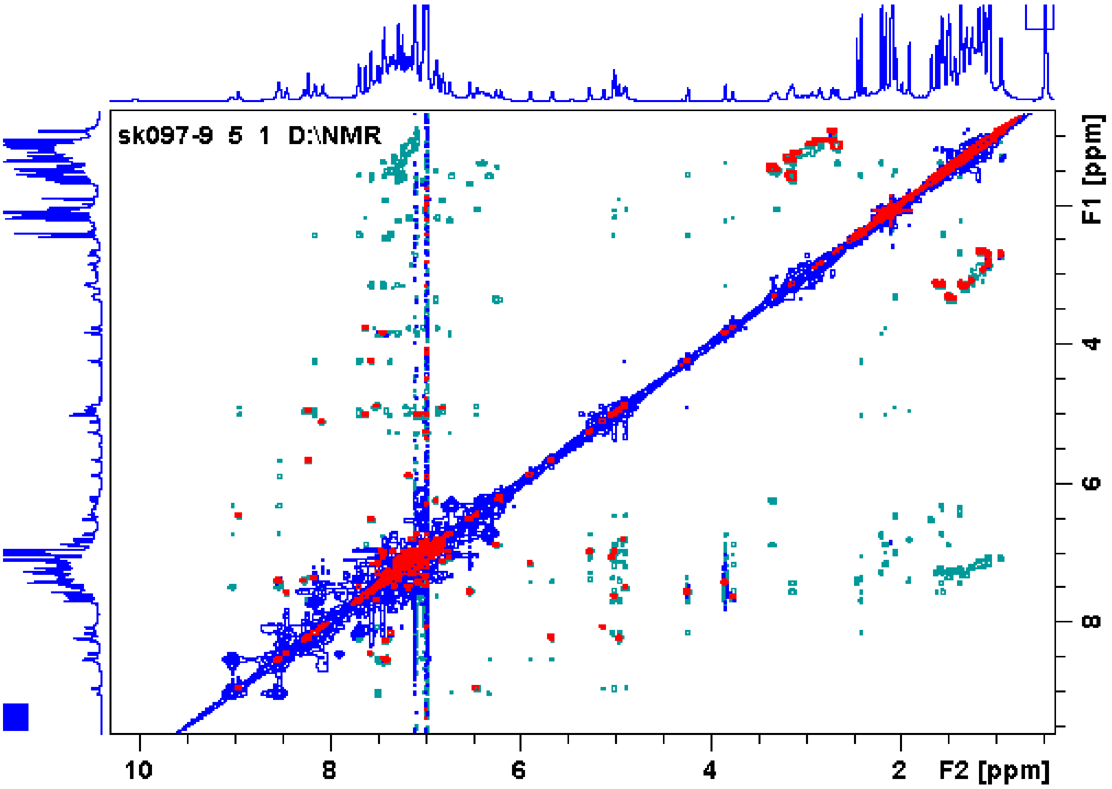


**Supplementary Figure 13. Correlation NMR spectroscopy for 1 and 2.** Overlay of partial COSY (red) and ROESY (green/blue) of partly converted heterodimer (**1** + **2**) recorded in toluene-$d_8$ at room temperature.

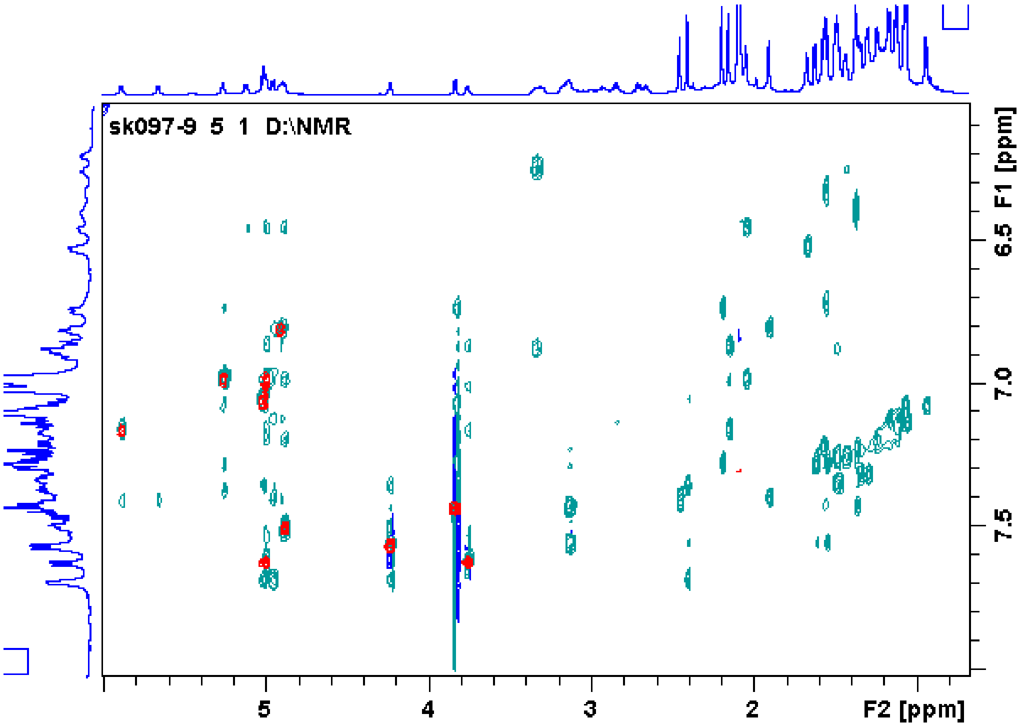


**Supplementary Figure 14. Correlation NMR spectroscopy for 1 and 2.** Overlay of partial COSY (red) and ROESY (green/blue) spectrum of partly converted heterodimer (**1** + **2**) recorded in toluene-$d_8$ at room temperature.

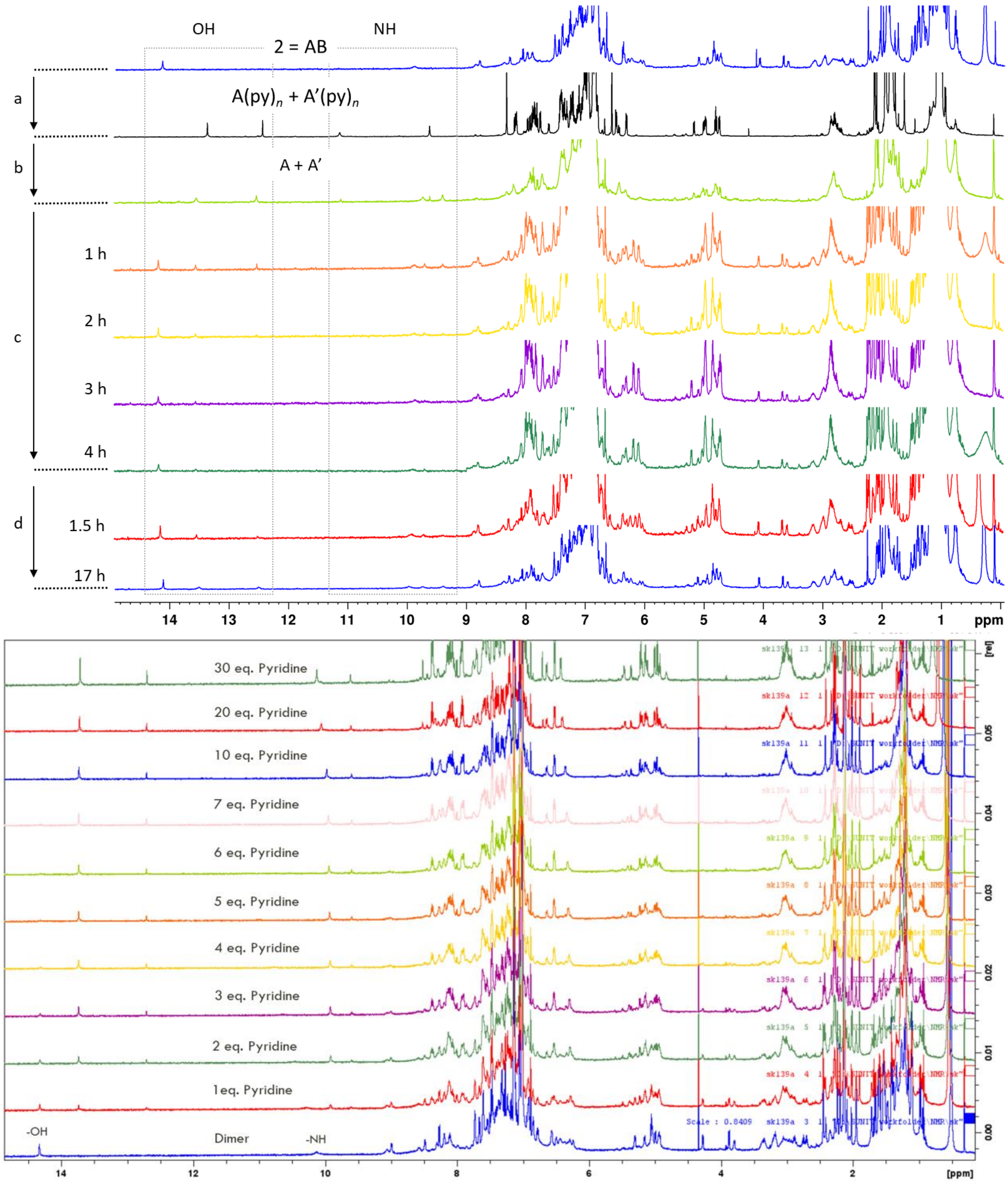


**Supplementary Figure 15. Cleavage and reconstitution of 2.** Top: Full range $^{1}$H NMR spectra of cleavage and reconstitution experiment of **2.** Reagents and conditions: (a) pyridine-$d_5$ (ca. 180 equiv); (b) i). solvent evaporation (5 cycles, fresh toluene-$d_8$ added before each cycle), ii). evacuation (15 h), iii). toluene-$d_8$, 4 Å molecular sieves (powder); (c) heating (90 °C), 4 h; (d) solvent evaporation, fresh toluene-$d_8$ added. Bottom: Sequence of changes observed during titration of the **2** with pyridine-$d_5$.

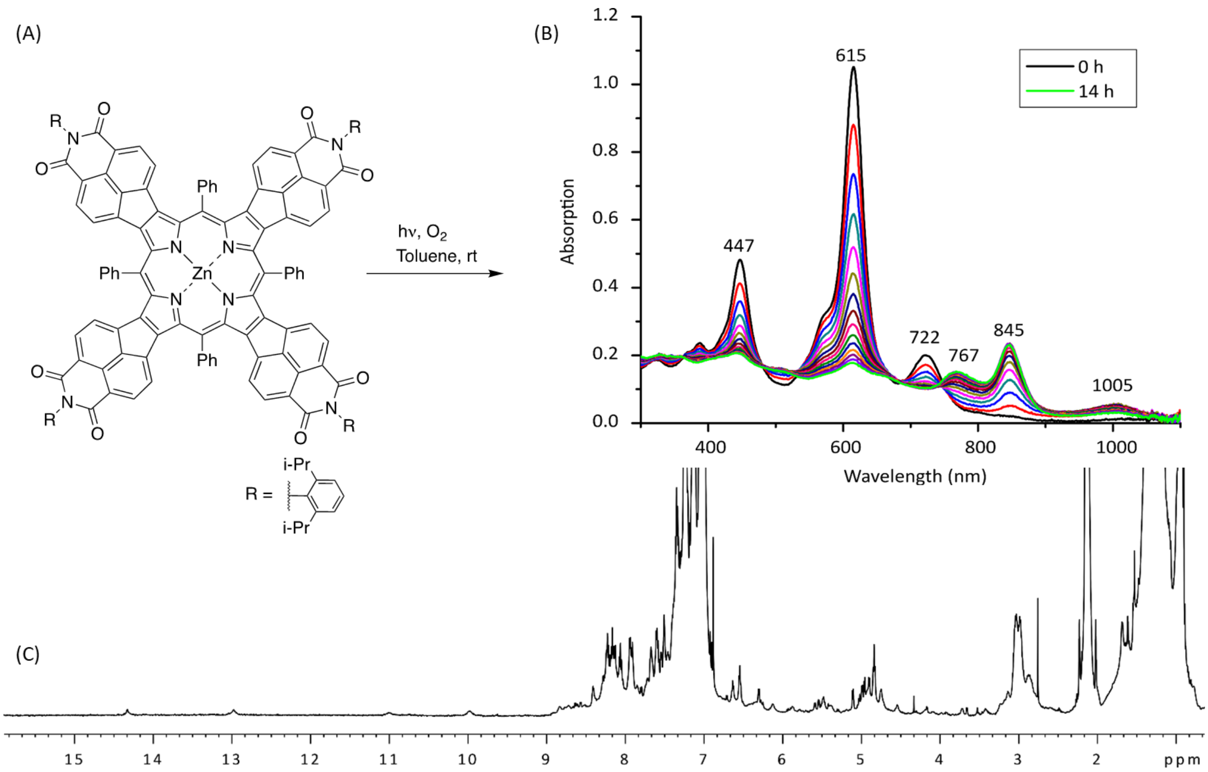


**Supplementary Figure 16. Photocleavage of zinc(II) tetra-NMI porphyrin.** (A) Reaction scheme. (B) Changes of UV-vis-NIR absorption spectrum occurring during photoirradiation of a 0.01 mM solution of zinc(II) tetra NMI porphyrin in toluene at room temperature and (C) $^{1}$H NMR spectrum of zinc(II) tetra-NMI porphyrin after photoirradiation recorded in toluene-$d_8$ at room temperature.

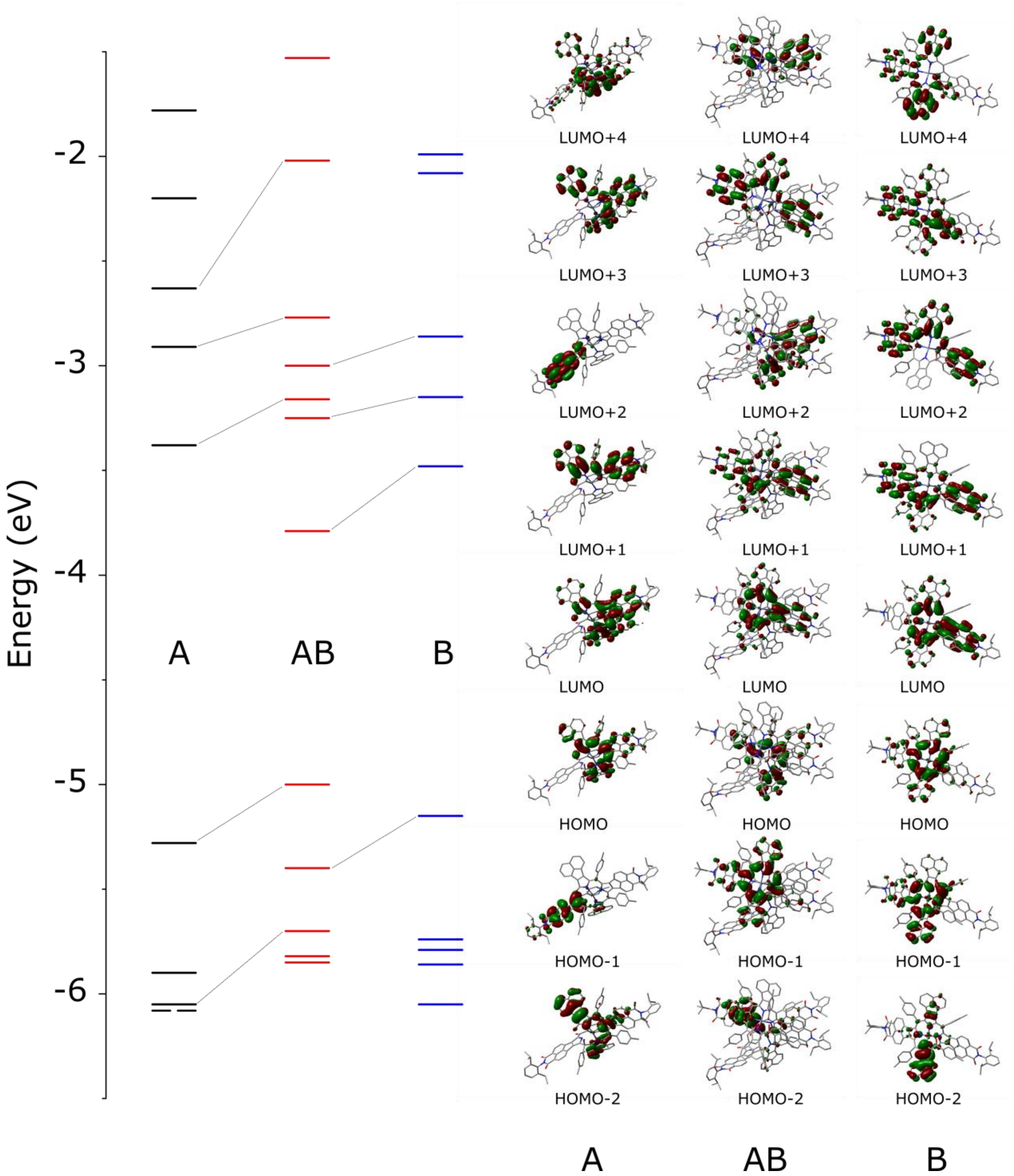


**Supplementary Figure 17. Molecular orbitals for 2 and its subunits.** Frontier molecular orbitals (isovalue=0.02) and orbital energies of subunit **A**, heterodimer **AB** (= **2**), and subunit **B**.

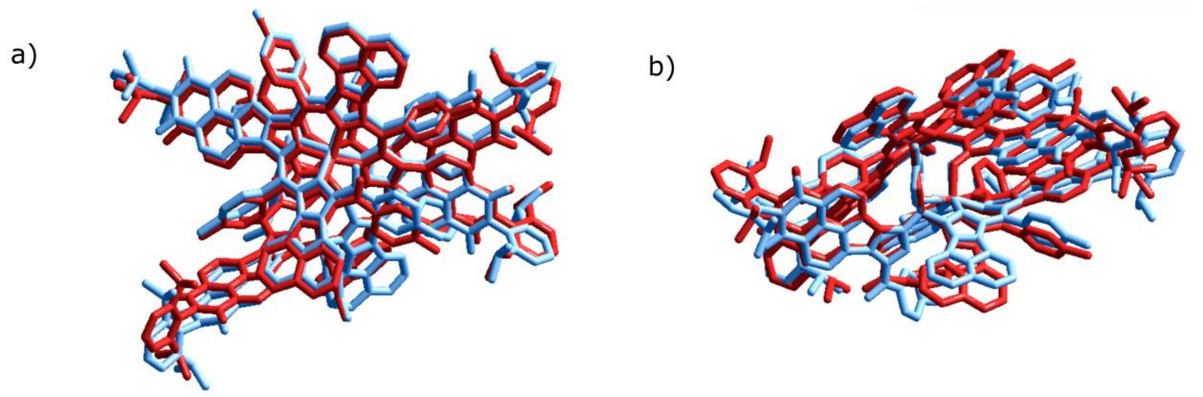


**Supplementary Figure 18. Molecular geometry of 2.** Comparision of the experimental (XRD, blue) and calculated B3LYP-GD3BJ/6-31G(d,p) (red) geometries of heterodimer **AB** (= **2**). Two perspective views a) and b) are shown for clarity.

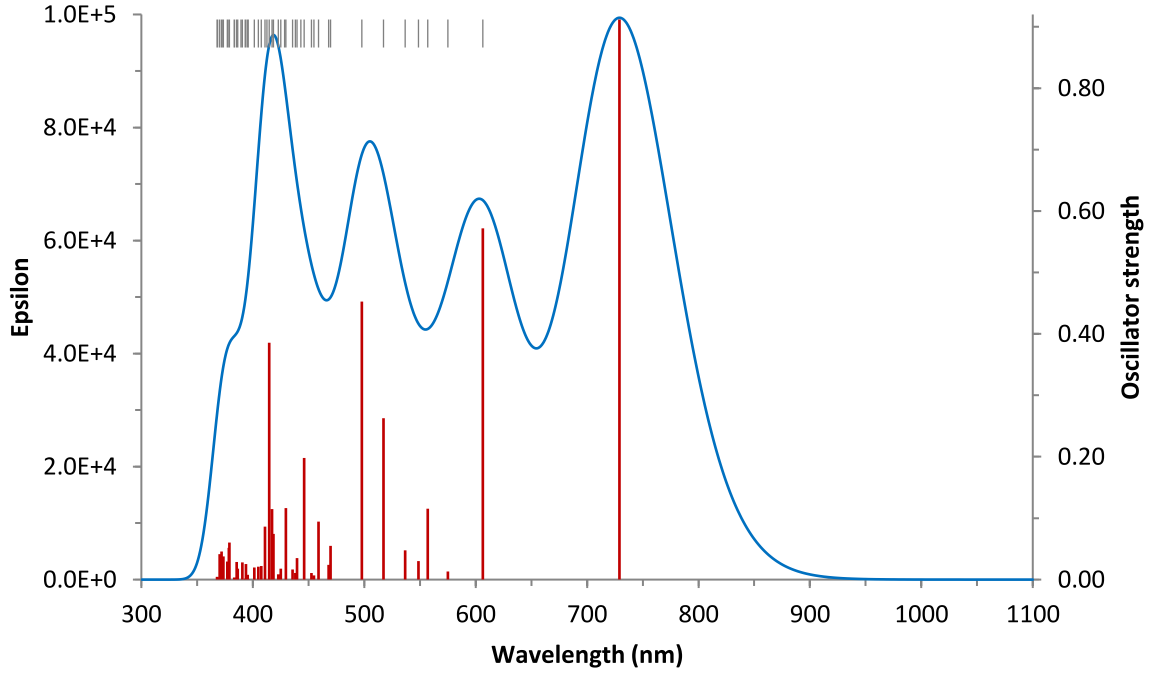


**Supplementary Figure 19. Simulated absorption spectra.** Electronic absorption spectrum of subunit **A** simulated using time-dependent density functional theory (B3LYP-GD3BJ/6-31G(d)/SMD(toluene), 50 transitions, linewidth factor 2000 $cm^{-1}$).

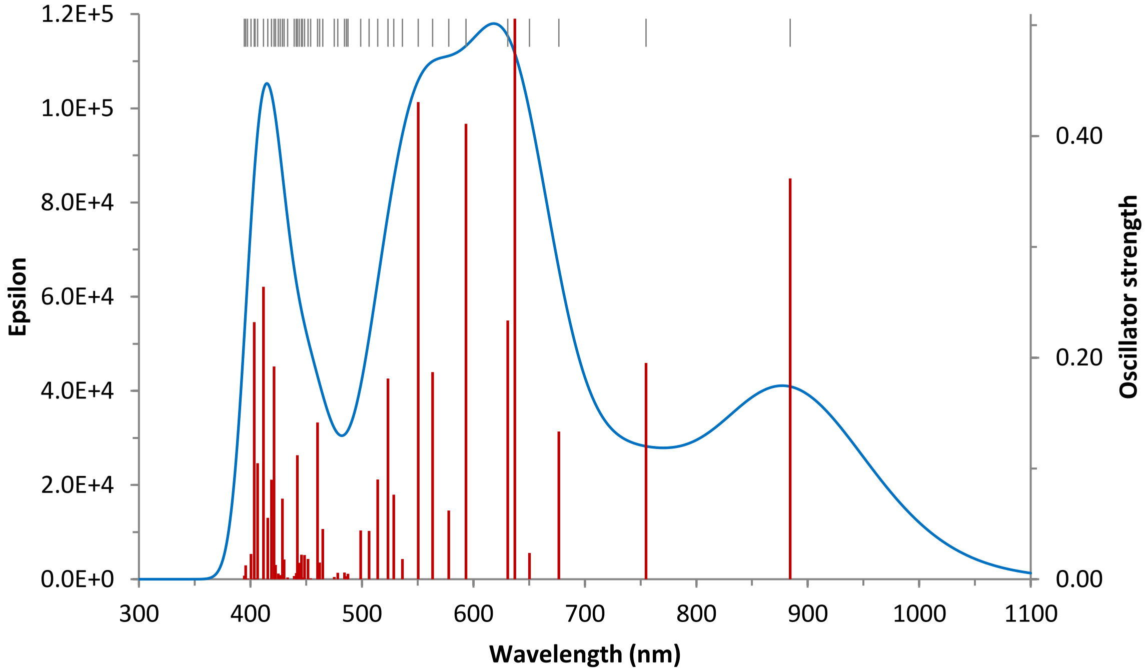


**Supplementary Figure 20. Simulated absorption spectra.** Electronic absorption spectrum of subunit **B** simulated using time-dependent density functional theory (B3LYP-GD3BJ /6-31G(d)/SMD(toluene), 50 transitions, linewidth factor 2000 cm$^{-1}$).

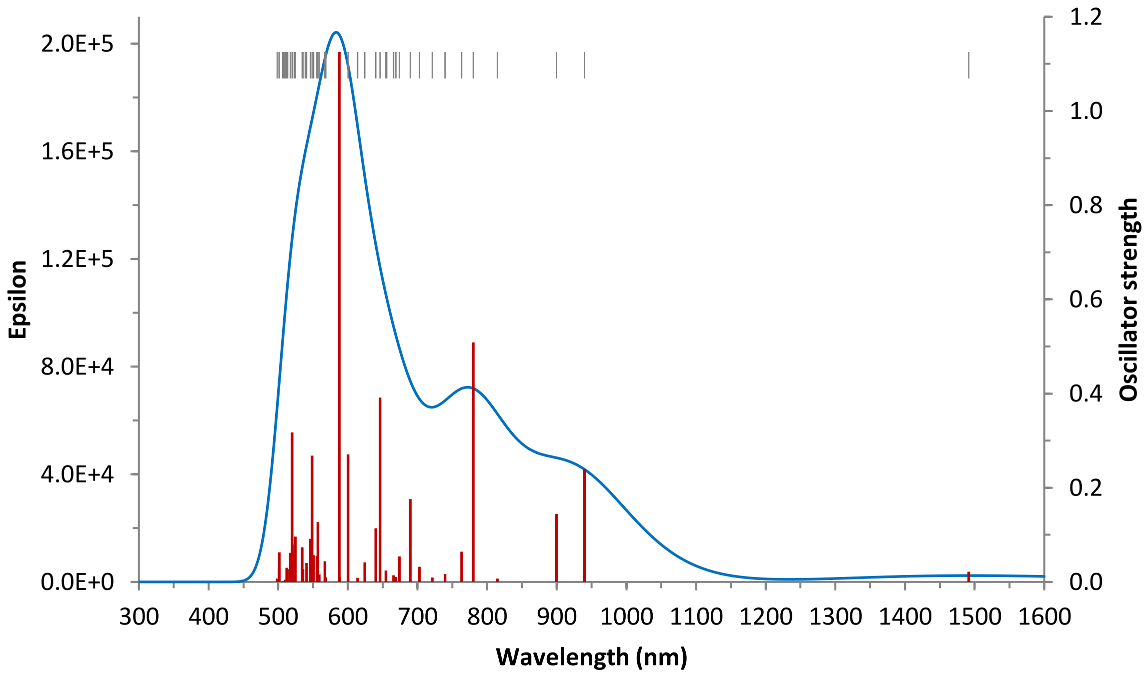


**Supplementary Figure 21. Simulated absorption spectra.** Electronic absorption spectrum of heterodimer **AB** (= **2**) simulated using time-dependent density functional theory (B3LYP-GD3BJ /6-31G(d)/SMD(toluene), 50 transitions, linewidth factor 2000 cm$^{-1}$).

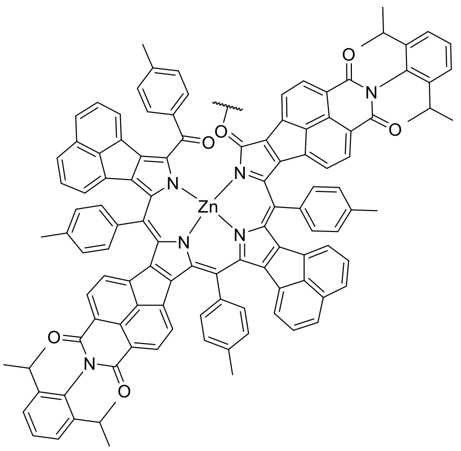


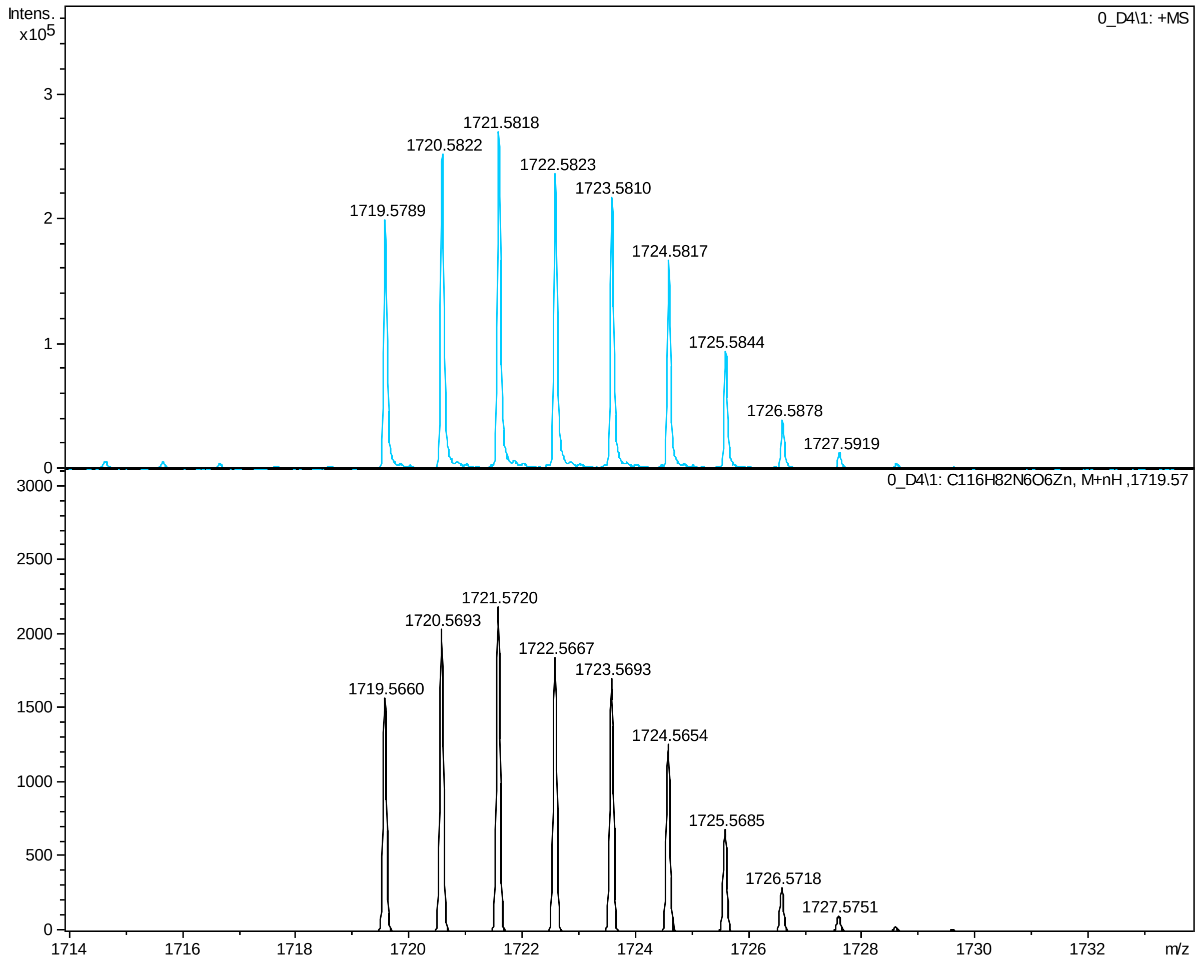


**Supplementary Figure 22. Mass Spectrometry.** High resolution mass spectrum of heterodimer **2**; subunit **B** (MALDI–TOF, top: experimental, bottom: simulated).

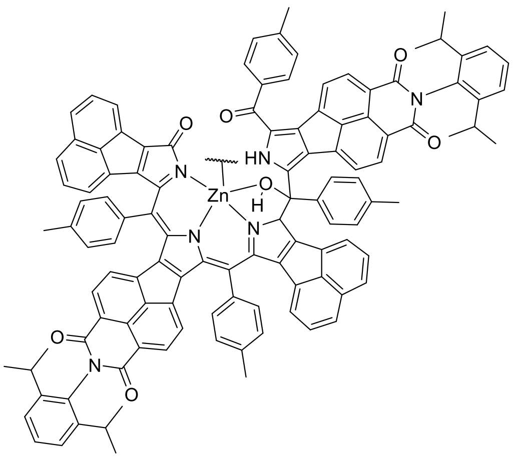


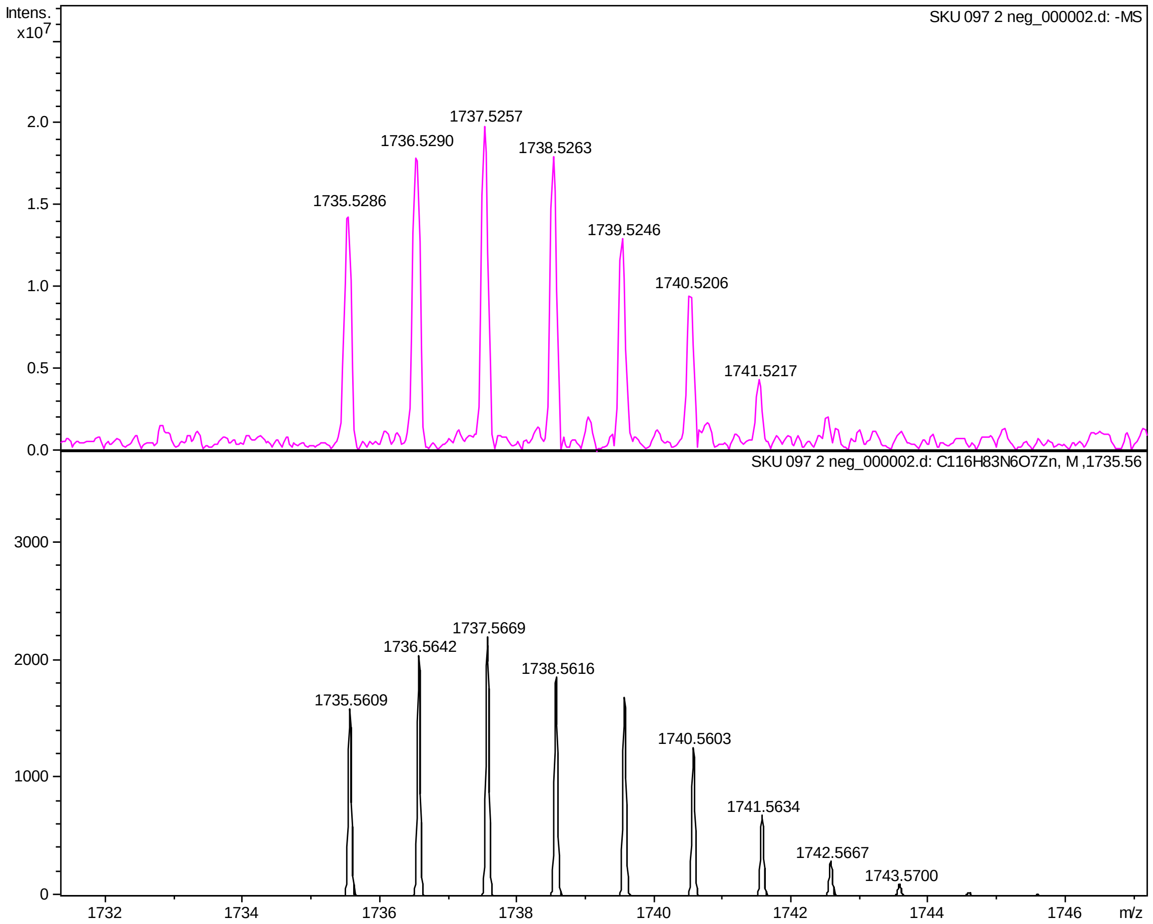


**Supplementary Figure 23. Mass Spectrometry.** High resolution mass spectrum of heterodimer **2**; subunit **A** (ESI–TOF, top: experimental, bottom: simulated).

# Supplementary Tables

**Supplementary Table 1.** Computational details.

| Code[a] | SCF *E* [b] a.u. | SCF *E* [c] a.u. | ZPV [d] $cm^{-1}$ | lowest freq.[e] $cm^{-1}$ | G [f] a.u. |
|---|---|---|---|---|---|
| **AB** | -14136.19162 | -14136.34946 | 3.378647 | 7.4265 | -14133.23654 |
| **A'B** | -14136.18187 | -14136.34029 | 3.378814 | 4.8799 | -14133.22626 |
| **AB'** | -14136.17388 | -14136.33265 | 3.379161 | 5.5876 | -14133.22038 |
| **A'B'** | -14136.16705 | -14136.33118 | 3.378186 | 4.9921 | -14133.22295 |
| **A** | -7106.21156 | -7106.316181 | 1.699572 | 3.9976 | -7104.777392 |
| **A'** | -7106.212428 | -7106.317518 | 1.699774 | 3.8982 | -7104.777897 |
| **B** | -7029.78051 | -7029.879356 | 1.672218 | 5.4668 | -7028.36214 |
| **B'** | -7029.778479 | -7029.878285 | 1.671961 | 4.6244 | -7028.361899 |
| **1** | -6879.319946 | -6879.421927 | 1.664987 | 5.4500 | -6877.910506 |
| **$H_2O$** | -76.42031125 | -76.42433418 | 0.021363 | 1665.2314 | -76.42062793 |
| **$O_2$** | -150.3204958 | -150.3238332 | 0.00378 | 1659.0858 | -150.3400324 |

[a] Structure code (see the zip file for Cartesian coordinates). [b] SCF electronic energy obtained from gas-phase optimized geometries. [c] SCF electronic energy obtained from single-point energy calculation with SMD solvation. [d] Zero-point vibrational energy obtained from gas-phase optimized geometries. [e] Lowest vibrational frequency obtained from gas-phase optimized geometries. [f] SMD solvated Gibbs free energy.

**Supplementary Table 2.** MO composition analysis of the selected molecular orbitals of heterodimer **2** obtained at B3LYP-GD3BJ/6-31G(d,p) level of theory.

| MO levels | MO energy (eV) | % Composition of subunit A | % Composition of subunit B |
|---|---|---|---|
| **L+6** | -2.19 | 2 | 98 |
| **L+5** | -2.68 | 99 | 1 |
| **L+4** | -2.77 | 94 | 6 |
| **L+3** | -3.00 | 4 | 96 |
| **L+2** | -3.16 | 96 | 4 |
| **L+1** | -3.25 | 4 | 96 |
| **LUMO** | -3.79 | 3 | 97 |
| **HOMO** | -5.00 | 96 | 4 |
| **H-1** | -5.40 | 3 | 97 |
| **H-2** | -5.70 | 84 | 16 |
| **H-3** | -5.82 | 99 | 1 |
| **H-4** | -5.85 | 96 | 4 |

**Supplementary Table 3.** Electronic transitions calculated for subunit **A** using the TDA/SMD(toluene)/B3LYP-GD3BJ/6-31G(d,p) level of theory.

| No. | Energy (cm$^{-1}$) | λ (nm) | f[a] | Major excitations[b] |
|---|---|---|---|---|
| 1 | 13716 | 729.1 | 0.912 | HOMO»LUMO (97%) |
| 2 | 16489 | 606.5 | 0.572 | HOMO»L+1 (91%) |
| 3 | 17389 | 575.1 | 0.013 | HOMO»L+2 (99%) |
| 4 | 17953 | 557.0 | 0.115 | H–4»LUMO (13%)<br>H–3»LUMO (26%)<br>H–1»LUMO (54%) |
| 5 | 18226 | 548.7 | 0.030 | H–3»LUMO (12%)<br>H–2»LUMO (72%) |
| 6 | 18631 | 536.8 | 0.048 | H–3»LUMO (55%)<br>H–1»LUMO (36%) |
| 7 | 19331 | 517.3 | 0.263 | H–5»LUMO (43%)<br>H–4»LUMO (27%) |
| 8 | 20086 | 497.9 | 0.453 | H–5»LUMO (34%)<br>H–4»LUMO (43%) |
| 9 | 21286 | 469.8 | 0.055 | H–8»LUMO (12%)<br>H–7»LUMO (12%)<br>H–5»LUMO (14%)<br>H–2»L+1 (41%) |
| 10 | 21365 | 468.1 | 0.024 | H–8»LUMO (39%)<br>H–7»LUMO (30%)<br>H–2»L+1 (17%) |
| 11 | 21787 | 459.0 | 0.094 | H–3»L+1 (10%)<br>H–1»L+1 (35%)<br>HOMO»L+3 (35%) |
| 12 | 21984 | 454.9 | 0.007 | H–8»LUMO (35%)<br>H–7»LUMO (40%) |
| 13 | 22092 | 452.6 | 0.011 | H–5»L+1 (21%)<br>H–4»L+1 (44%) |
| 14 | 22417 | 446.1 | 0.198 | H–9»LUMO (10%)<br>H–1»L+1 (45%)<br>HOMO»L+3 (23%) |
| 15 | 22567 | 443.1 | 0.000 | H–6»LUMO (99%) |
| 16 | 22743 | 439.7 | 0.035 | H–9»LUMO (59%) |
| 17 | 22820 | 438.2 | 0.011 | H–8»L+2 (18%)<br>H–7»L+2 (11%)<br>H–3»L+2 (25%)<br>H–1»L+2 (22%) |
| 18 | 22951 | 435.7 | 0.017 | H–3»L+1 (55%)<br>H–2»L+1 (16%) |
| 19 | 23273 | 429.7 | 0.116 | H–8»L+2 (21%)<br>H–7»L+2 (14%)<br>H–1»L+2 (52%) |
| 20 | 23334 | 428.6 | 0.000 | H–12»LUMO (71%)<br>H–11»LUMO (15%) |
| 21 | 23523 | 425.1 | 0.018 | H–12»LUMO (14%)<br>H–11»LUMO (61%) |
| 22 | 23647 | 422.9 | 0.009 | H–13»LUMO (84%) |
| 23 | 23893 | 418.5 | 0.074 | H–5»L+1 (23%)<br>H–4»L+1 (14%)<br>H–2»L+2 (44%) |
| 24 | 23965 | 417.3 | 0.115 | H–5»L+1 (25%)<br>H–4»L+1 (12%)<br>H–2»L+2 (45%) |
| 25 | 24112 | 414.7 | 0.386 | H–3»L+2 (62%)<br>H–1»L+2 (15%) |
| 26 | 24231 | 412.7 | 0.001 | H–10»LUMO (94%) |
| 27 | 24335 | 410.9 | 0.086 | H–14»LUMO (77%) |
| 28 | 24533 | 407.6 | 0.022 | H–21»LUMO (14%)<br>H–20»LUMO (32%)<br>H–19»LUMO (11%) |
| 29 | 24692 | 405.0 | 0.021 | HOMO»L+4 (80%) |
| 30 | 24914 | 401.4 | 0.020 | H–6»L+2 (97%) |
| 31 | 25260 | 395.9 | 0.001 | H–4»L+2 (79%) |
| 32 | 25290 | 395.4 | 0.008 | H–8»L+1 (16%)<br>H–7»L+1 (74%) |
| 33 | 25390 | 393.9 | 0.025 | H–16»LUMO (70%) |
| 34 | 25433 | 393.2 | 0.002 | H–8»L+1 (75%)<br>H–7»L+1 (17%) |
| 35 | 25598 | 390.6 | 0.028 | H–15»LUMO (83%) |
| 36 | 25681 | 389.4 | 0.000 | H–10»L+2 (86%) |
| 37 | 25874 | 386.5 | 0.018 | H–19»LUMO (12%)<br>HOMO»L+5 (53%) |
| 38 | 25939 | 385.5 | 0.029 | H–18»LUMO (58%)<br>H–9»L+1 (10%) |
| 39 | 26069 | 383.6 | 0.002 | H–5»L+2 (78%)<br>H–4»L+2 (10%) |
| 40 | 26077 | 383.5 | 0.001 | H–17»LUMO (18%)<br>H–6»L+1 (76%) |
| 41 | 26084 | 383.4 | 0.003 | H–17»LUMO (64%)<br>H–6»L+1 (22%) |
| 42 | 26385 | 379.0 | 0.060 | H–19»LUMO (19%)<br>H–11»L+1 (20%)<br>H–9»L+1 (31%) |
| 43 | 26419 | 378.5 | 0.052 | H–20»LUMO (18%)<br>H–19»LUMO (20%)<br>H–11»L+1 (21%) |
| 44 | 26515 | 377.1 | 0.029 | H–26»LUMO (18%)<br>H–12»L+1 (41%) |
| 45 | 26766 | 373.6 | 0.038 | H–23»LUMO (18%)<br>H–21»LUMO (12%)<br>H–11»L+1 (18%)<br>H–9»L+1 (20%) |
| 46 | 26848 | 372.5 | 0.031 | H–23»LUMO (11%)<br>H–22»LUMO (24%)<br>H–21»LUMO (14%) |
| 47 | 26883 | 372.0 | 0.046 | H–11»L+1 (11%)<br>H–1»L+3 (17%)<br>HOMO»L+5 (16%) |
| 48 | 27007 | 370.3 | 0.041 | H–14»L+2 (12%)<br>H–13»L+2 (36%) |
| 49 | 27140 | 368.5 | 0.003 | H–26»LUMO (10%)<br>H–2»L+3 (12%)<br>H–1»L+3 (13%) |
| 50 | 27179 | 367.9 | 0.005 | H–26»LUMO (31%)<br>H–12»L+1 (32%) |

[a] Oscillator strength. [b] Contributions smaller than 10% are not included. H = HOMO, L = LUMO. Orbitals are numbered consecutively regardless of possible degeneracies.

**Supplementary Table 4.** Electronic transitions calculated for subunit **B** using the TDA/SMD(toluene)/B3LYP-GD3BJ/6-31G(d,p) level of theory.

| No. | Energy ($cm^{-1}$) | λ (nm) | f[a] | Major excitations[b] |
|---|---|---|---|---|
| 1 | 11310 | 884.2 | 0.362 | HOMO»LUMO (95%) |
| 2 | 13247 | 754.9 | 0.195 | H–1»LUMO (22%)<br>HOMO»L+1 (74%) |
| 3 | 14778 | 676.7 | 0.133 | H–2»LUMO (75%) |
| 4 | 15376 | 650.4 | 0.024 | H–3»LUMO (93%) |
| 5 | 15693 | 637.2 | 0.506 | H–5»LUMO (10%)<br>H–4»LUMO (10%)<br>H–2»LUMO (10%)<br>H–1»LUMO (41%)<br>HOMO»L+2 (13%) |
| 6 | 15851 | 630.9 | 0.234 | HOMO»L+2 (75%) |
| 7 | 16852 | 593.4 | 0.411 | H–5»LUMO (11%)<br>H–4»LUMO (57%)<br>H–1»LUMO (14%) |
| 8 | 17303 | 577.9 | 0.062 | H–5»LUMO (51%)<br>H–4»LUMO (11%)<br>H–1»L+1 (24%) |
| 9 | 17747 | 563.5 | 0.187 | H–2»L+1 (65%)<br>H–1»L+1 (19%) |
| 10 | 18164 | 550.6 | 0.431 | H–5»LUMO (11%)<br>H–2»L+1 (27%)<br>H–1»L+1 (36%) |
| 11 | 18644 | 536.4 | 0.018 | H–3»L+1 (86%) |
| 12 | 18918 | 528.6 | 0.076 | H–6»LUMO (66%)<br>H–5»L+1 (11%) |
| 13 | 19105 | 523.4 | 0.181 | H–6»LUMO (22%)<br>H–5»L+1 (16%)<br>H–4»L+1 (35%) |
| 14 | 19447 | 514.2 | 0.090 | H–11»LUMO (28%)<br>H–5»L+1 (30%) |
| 15 | 19744 | 506.5 | 0.044 | H–11»LUMO (27%)<br>H–5»L+1 (13%)<br>H–4»L+1 (33%) |
| 16 | 20042 | 499.0 | 0.044 | H–3»L+2 (13%)<br>H–1»L+2 (65%) |
| 17 | 20511 | 487.6 | 0.005 | H–8»LUMO (91%) |
| 18 | 20570 | 486.1 | 0.003 | H–9»LUMO (50%)<br>H–7»LUMO (24%) |
| 19 | 20644 | 484.4 | 0.006 | H–9»LUMO (10%)<br>H–3»L+2 (33%)<br>H–2»L+2 (23%)<br>H–1»L+2 (16%) |
| 20 | 20904 | 478.4 | 0.006 | H–3»L+2 (38%)<br>H–2»L+2 (50%) |
| 21 | 21043 | 475.2 | 0.002 | H–9»LUMO (29%)<br>H–7»LUMO (63%) |
| 22 | 21509 | 464.9 | 0.045 | H–6»L+1 (49%)<br>HOMO»L+3 (25%) |
| 23 | 21641 | 462.1 | 0.015 | H–22»LUMO (13%)<br>H–19»LUMO (13%)<br>H–6»L+1 (21%)<br>HOMO»L+3 (14%) |
| 24 | 21728 | 460.2 | 0.142 | H–13»LUMO (10%)<br>H–12»LUMO (13%)<br>H–6»L+1 (16%)<br>HOMO»L+3 (12%) |
| 25 | 22025 | 454.0 | 0.001 | H–12»LUMO (57%)<br>H–11»LUMO (17%) |
| 26 | 22138 | 451.7 | 0.018 | H–16»LUMO (10%)<br>H–13»LUMO (53%) |
| 27 | 22292 | 448.6 | 0.022 | H–4»L+2 (60%) |
| 28 | 22391 | 446.6 | 0.002 | H–7»L+1 (13%)<br>H–5»L+2 (45%) |
| 29 | 22430 | 445.8 | 0.022 | H–7»L+1 (66%)<br>H–5»L+2 (11%) |
| 30 | 22533 | 443.8 | 0.015 | H–18»LUMO (12%)<br>H–16»LUMO (34%) |
| 31 | 22618 | 442.1 | 0.112 | HOMO»L+4 (50%) |
| 32 | 22671 | 441.1 | 0.006 | H–10»LUMO (91%) |
| 33 | 22765 | 439.3 | 0.003 | H–14»LUMO (81%) |
| 34 | 23073 | 433.4 | 0.002 | H–15»LUMO (50%)<br>H–9»L+1 (16%) |
| 35 | 23243 | 430.2 | 0.018 | H–18»LUMO (24%)<br>H–17»LUMO (36%)<br>H–11»L+1 (16%) |
| 36 | 23325 | 428.7 | 0.073 | H–18»LUMO (10%)<br>H–15»LUMO (24%)<br>H–9»L+1 (36%) |
| 37 | 23434 | 426.7 | 0.004 | H–19»LUMO (18%)<br>H–11»L+1 (36%) |
| 38 | 23529 | 425.0 | 0.005 | H–8»L+1 (83%) |
| 39 | 23666 | 422.5 | 0.013 | H–10»L+1 (70%)<br>H–10»L+2 (12%) |
| 40 | 23740 | 421.2 | 0.192 | H–18»LUMO (21%)<br>H–17»LUMO (17%)<br>H–16»LUMO (11%)<br>H–9»L+1 (21%) |
| 41 | 23872 | 418.9 | 0.090 | H–6»L+2 (78%) |
| 42 | 24062 | 415.6 | 0.055 | H–22»LUMO (16%)<br>H–20»LUMO (44%)<br>H–19»LUMO (10%) |
| 43 | 24288 | 411.7 | 0.264 | H–22»LUMO (17%)<br>H–20»LUMO (35%)<br>H–19»LUMO (17%)<br>HOMO»L+5 (11%) |
| 44 | 24602 | 406.5 | 0.105 | H–24»LUMO (29%)<br>H–23»LUMO (29%) |
| 45 | 24740 | 404.2 | 0.001 | H–21»LUMO (61%)<br>H–13»L+1 (14%) |
| 46 | 24787 | 403.4 | 0.232 | HOMO»L+5 (39%) |
| 47 | 24967 | 400.5 | 0.023 | H–26»LUMO (13%)<br>H–21»LUMO (20%)<br>H–13»L+1 (18%) |
| 48 | 25162 | 397.4 | 0.001 | H–12»L+1 (38%)<br>H–11»L+1 (10%) |
| 49 | 25267 | 395.8 | 0.013 | H–2»L+3 (25%)<br>H–2»L+4 (35%) |
| 50 | 25348 | 394.5 | 0.003 | H–24»LUMO (12%)<br>H–16»L+1 (21%)<br>H–14»L+1 (12%) |

[a] Oscillator strength. [b] Contributions smaller than 10% are not included. H = HOMO, L = LUMO. Orbitals are numbered consecutively regardless of possible degeneracies.

**Supplementary Table 5.** Electronic transitions calculated for heterodimer **AB** (= **2**) using the TD/PCM(toluene)/B3LYP-GD3BJ/6-31G(d,p) level of theory.

| No. | Energy (cm$^{-1}$) | λ (nm) | f[a] | Major excitations[b] |
|---|---|---|---|---|
| 1 | 6704 | 1491.6 | 0.022 | HOMO»LUMO (99%) |
| 2 | 10639 | 939.9 | 0.239 | H–1»LUMO (76%)<br>HOMO»L+1 (21%) |
| 3 | 11117 | 899.5 | 0.144 | H–1»LUMO (19%)<br>HOMO»L+1 (77%) |
| 4 | 12275 | 814.7 | 0.007 | H–2»LUMO (95%) |
| 5 | 12819 | 780.1 | 0.509 | HOMO»L+2 (91%) |
| 6 | 13099 | 763.4 | 0.064 | HOMO»L+3 (93%) |
| 7 | 13522 | 739.5 | 0.017 | H–4»LUMO (69%)<br>H–3»LUMO (18%) |
| 8 | 13865 | 721.2 | 0.009 | H–5»LUMO (15%)<br>H–3»LUMO (52%) |
| 9 | 14228 | 702.9 | 0.032 | H–7»LUMO (47%)<br>H–6»LUMO (16%)<br>H–3»LUMO (11%) |
| 10 | 14500 | 689.6 | 0.176 | H–6»LUMO (19%)<br>H–1»L+1 (57%) |
| 11 | 14838 | 673.9 | 0.054 | H–8»LUMO (42%)<br>H–6»LUMO (12%)<br>H–5»LUMO (20%) |
| 12 | 14948 | 669.0 | 0.011 | H–1»L+2 (90%) |
| 13 | 15022 | 665.7 | 0.014 | H–8»LUMO (48%)<br>H–6»LUMO (13%) |
| 14 | 15248 | 655.8 | 0.001 | HOMO»L+4 (47%)<br>HOMO»L+5 (52%) |
| 15 | 15276 | 654.6 | 0.024 | H–7»LUMO (27%)<br>H–6»LUMO (16%)<br>H–5»LUMO (39%) |
| 16 | 15475 | 646.2 | 0.391 | HOMO»L+4 (36%)<br>HOMO»L+5 (35%) |
| 17 | 15623 | 640.1 | 0.114 | H–11»LUMO (15%)<br>H–10»LUMO (47%)<br>H–9»LUMO (12%)<br>H–6»LUMO (10%) |
| 18 | 16019 | 624.3 | 0.042 | H–11»LUMO (59%)<br>H–10»LUMO (15%)<br>H–1»L+3 (10%) |
| 19 | 16286 | 614.0 | 0.009 | H–2»L+1 (78%) |
| 20 | 16659 | 600.3 | 0.271 | H–9»LUMO (19%)<br>H–1»L+3 (59%) |
| 21 | 16993 | 588.5 | 0.010 | H–4»L+2 (14%)<br>H–2»L+2 (69%) |
| 22 | 17016 | 587.7 | 1.125 | H–10»LUMO (23%)<br>H–9»LUMO (29%)<br>H–1»L+1 (10%)<br>H–1»L+3 (14%) |
| 23 | 17596 | 568.3 | 0.010 | H–4»L+1 (17%)<br>H–4»L+2 (27%)<br>H–3»L+2 (17%)<br>H–2»L+2 (11%) |
| 24 | 17637 | 567.0 | 0.044 | H–1»L+4 (45%)<br>H–1»L+5 (39%) |
| 25 | 17893 | 558.9 | 0.016 | H–12»LUMO (14%)<br>H–4»L+1 (32%)<br>H–4»L+2 (13%) |
| 26 | 17955 | 557.0 | 0.127 | H–14»LUMO (28%)<br>H–13»LUMO (52%) |
| 27 | 18001 | 555.5 | 0.055 | H–12»LUMO (78%) |
| 28 | 18155 | 550.8 | 0.057 | H–13»LUMO (10%)<br>H–3»L+1 (46%) |
| 29 | 18227 | 548.7 | 0.268 | H–17»LUMO (28%)<br>H–16»LUMO (19%)<br>H–14»LUMO (12%) |
| 30 | 18306 | 546.3 | 0.092 | H–6»L+2 (14%)<br>H–4»L+2 (13%)<br>H–3»L+1 (12%)<br>H–3»L+2 (32%) |
| 31 | 18493 | 540.7 | 0.040 | H–2»L+3 (57%) |
| 32 | 18557 | 538.9 | 0.001 | H–1»L+4 (46%)<br>H–1»L+5 (51%) |
| 33 | 18673 | 535.5 | 0.027 | H–19»LUMO (52%) |
| 34 | 18712 | 534.4 | 0.073 | H–19»LUMO (10%)<br>H–7»L+1 (30%)<br>H–6»L+1 (11%)<br>H–5»L+1 (17%)<br>H–4»L+1 (10%) |
| 35 | 19061 | 524.6 | 0.096 | H–14»LUMO (14%)<br>H–6»L+1 (15%)<br>H–5»L+1 (26%) |
| 36 | 19096 | 523.7 | 0.008 | H–19»LUMO (14%)<br>H–16»LUMO (11%)<br>H–14»LUMO (23%)<br>H–13»LUMO (12%)<br>H–5»L+1 (16%) |
| 37 | 19205 | 520.7 | 0.064 | H–20»LUMO (12%)<br>H–18»LUMO (57%) |
| 38 | 19213 | 520.5 | 0.079 | H–20»LUMO (44%)<br>H–18»LUMO (13%) |
| 39 | 19233 | 519.9 | 0.318 | H–18»LUMO (21%)<br>H–6»L+2 (15%)<br>H–5»L+2 (32%)<br>H–3»L+2 (10%) |
| 40 | 19328 | 517.4 | 0.062 | H–7»L+1 (24%)<br>H–6»L+1 (29%)<br>H–5»L+2 (10%) |
| 41 | 19473 | 513.5 | 0.027 | H–10»L+1 (11%)<br>H–9»L+1 (24%)<br>H–8»L+1 (38%) |
| 42 | 19524 | 512.2 | 0.030 | H–6»L+2 (23%)<br>H–5»L+2 (32%) |
| 43 | 19562 | 511.2 | 0.000 | H–16»LUMO (11%)<br>H–15»LUMO (87%) |
| 44 | 19602 | 510.1 | 0.005 | H–10»L+1 (21%)<br>H–8»L+1 (14%) |
| 45 | 19677 | 508.2 | 0.000 | H–17»LUMO (13%)<br>H–16»LUMO (10%)<br>H–2»L+4 (19%)<br>H–2»L+5 (16%) |
| 46 | 19697 | 507.7 | 0.002 | H–20»LUMO (14%)<br>H–17»LUMO (14%)<br>H–16»LUMO (12%)<br>H–2»L+4 (14%)<br>H–2»L+5 (12%)<br>HOMO»L+6 (10%) |
| 47 | 19745 | 506.5 | 0.001 | HOMO»L+6 (43%)<br>HOMO»L+7 (12%) |
| 48 | 19940 | 501.5 | 0.063 | H–11»L+1 (47%)<br>H–8»L+1 (14%) |
| 49 | 19952 | 501.2 | 0.028 | H–11»L+2 (10%)<br>H–9»L+2 (15%)<br>H–7»L+2 (40%) |
| 50 | 20058 | 498.5 | 0.007 | H–23»LUMO (66%)<br>H–22»LUMO (21%) |

[a] Oscillator strength. [b] Contributions smaller than 10% are not included. H = HOMO, L = LUMO. Orbitals are numbered consecutively regardless of possible degeneracies.

**Supplementary Table 6.** Crystal data and structure refinement for heterodimer **2**·8.2 $C_7D_8$.

| **Identification code** | **2**·8.2 $C_7D_8$ (2019726) |
|---|---|
| Empirical formula | $C_{289.4}H_{231.6}N_{12}O_{13}Zn_2$ |
| Formula weight | 4282.00 |
| Temperature/K | 100(2) |
| Crystal system | monoclinic |
| Space group | $P2_1/n$ |
| *a*/Å | 28.172(8) |
| *b*/Å | 28.532(11) |
| *c*/Å | 29.868(7) |
| *α*/° | 90 |
| *β*/° | 103.05(3) |
| *γ*/° | 90 |
| Volume/Å$^3$ | 23388(13) |
| *Z* | 4 |
| $D_{calc}$g/cm$^3$ | 1.216 |
| $\mu$/mm$^{-1}$ | 0.766 |
| *F*(000) | 8864.0 |
| Crystal size/mm$^3$ | 0.15 × 0.15 × 0.15 |
| Radiation | Cu Kα (λ = 1.54184) |
| 2$\theta$ range for data collection/° | 6.984 to 133.998 |
| Index ranges | -32 ≤ h ≤ 33, -34 ≤ k ≤ 33, -35 ≤ l ≤ 32 |
| Reflections collected | 116728 |
| Independent reflections | 41556 [$R_{int}$ = 0.1798, $R_{sigma}$ = 0.2389] |
| Data/restraints/parameters | 41556/189/2552 |
| Goodness-of-fit on $F^2$ | 1.130 |
| Final *R* indexes [I>=2σ (I)] | $R_1$ = 0.1519, $wR_2$ = 0.3124 |
| Final R indexes [all data] | $R_1$ = 0.3382, $wR_2$ = 0.4250 |
| Largest diff. peak/hole / e Å$^{-3}$ | 0.71/-0.57 |

# Supplementary References


1. Sheldrick, G. M. Crystal structure refinement with SHELXL. *Acta Cryst C* **71**, 3–8 (2015).
2. Spackman, M. A. & Jayatilaka, D. Hirshfeld surface analysis. *CrystEngComm* **11**, 19–32 (2009).
3. Turner, M. J., McKinnon, J. J., Jayatilaka, D. & Spackman, M. A. Visualisation and characterisation of voids in crystalline materials. *CrystEngComm* **13**, 1804–1813 (2011).
4. Becke, A. D. Density-functional exchange-energy approximation with correct asymptotic behavior. *Phys. Rev., A* **38**, 3098–3100 (1988).
5. Becke, A. D. Density-functional thermochemistry. III. The role of exact exchange. *J. Chem. Phys.* **98**, 5648–5652 (1993).
6. Lee, C., Yang, W. & Parr, R. G. Development of the Colle-Salvetti correlation-energy formula into a functional of the electron density. *Phys. Rev. B* **37**, 785–789 (1988).
7. Grimme, S., Ehrlich, S. & Goerigk, L. Effect of the damping function in dispersion corrected density functional theory. *Journal of Computational Chemistry* **32**, 1456–1465 (2011).
8. Marenich, A. V., Cramer, C. J. & Truhlar, D. G. Universal Solvation Model Based on Solute Electron Density and on a Continuum Model of the Solvent Defined by the Bulk Dielectric Constant and Atomic Surface Tensions. *J. Phys. Chem. B* **113**, 6378–6396 (2009).
9. Hirata, S. & Head-Gordon, M. Time-dependent density functional theory within the Tamm–Dancoff approximation. *Chemical Physics Letters* **314**, 291–299 (1999).
10. Contreras-García, J. *et al.* NCIPLOT: A Program for Plotting Noncovalent Interaction Regions. *J. Chem. Theory Comput.* **7**, 625–632 (2011).
11. Boto, R. A. *et al.* NCIPLOT4: Fast, Robust, and Quantitative Analysis of Noncovalent Interactions. *J. Chem. Theory Comput.* **16**, 4150–4158 (2020).
12. Zhylitskaya, H., Cybińska, J., Chmielewski, P., Lis, T. & Stępień, M. Bandgap engineering in π-extended pyrroles. a modular approach to electron-deficient chromophores with multi-redox activity. *J. Am. Chem. Soc.* **138**, 11390–11398 (2016).

13. Kumar, S. *et al.* Porphyrin–Ryleneimide Hybrids: Tuning of Visible and Near-Infrared Absorption by Chromophore Desymmetrization. *Org. Lett.* **22**, 7202–7207 (2020).
