# Supplementary material for: Synthesis of a donor–acceptor heterodimer via trifunctional completive self-sorting
Source: Nat Commun. 2022 Jun 9;13:3204. doi: 10.1038/s41467-022-30859-7 (PMC9184498; doi:10.1038/s41467-022-30859-7)
Supplement: Supplementary file 2 — Description of Additional Supplementary Files [file 41467_2022_30859_MOESM2_ESM.pdf]

Description of Additional Supplementary files

File name: Supplementary Data 1

Description: Cartesian coordinates
