# Supplementary material for: Synthesis of a donor–acceptor heterodimer via trifunctional completive self-sorting
Source: Nat Commun. 2022 Jun 9;13:3204. doi: 10.1038/s41467-022-30859-7 (PMC9184498; doi:10.1038/s41467-022-30859-7)
Supplement: Supplementary file 3 — Supplementary Data File 1 cartesian coordinates [file 41467_2022_30859_MOESM3_ESM.docx]

**Supplementary Data file: Cartesian coordinates**

**1**

209

title

Zn -0.000000 -0.000000 1.428700

N 0.002400 2.025000 1.551000

C 1.123600 2.794700 1.802000

C 0.704400 4.048600 2.384500

C -0.694400 4.050300 2.385000

C -1.116800 2.797400 1.802600

C 1.189500 5.254600 3.072000

C 2.407600 5.784800 3.457500

H 3.342200 5.290800 3.234500

C 2.432500 7.007200 4.180800

H 3.398100 7.413100 4.467200

C 1.281600 7.685300 4.535900

C 0.009200 7.151800 4.187600

C -1.261800 7.688200 4.536800

C -2.414500 7.012800 4.182500

H -3.379000 7.420800 4.469500

C -2.392800 5.790300 3.459100

H -3.328700 5.298400 3.236800

C -1.176200 5.257300 3.072800

C 0.007600 5.955000 3.457900

C -2.415600 2.427200 1.410700

C -3.464700 3.475100 1.402200

C -3.323900 4.623800 0.610300

H -2.433900 4.739600 0.001200

C -4.313700 5.602000 0.596100

H -4.189600 6.480400 -0.030900

C -5.469000 5.473400 1.379800

C -5.604900 4.325500 2.171500

H -6.494300 4.201500 2.782800

C -4.622100 3.339000 2.181000

H -4.745100 2.453100 2.794700

C -2.786400 1.124900 1.022200

N -2.023300 0.002500 1.283500

C -4.031400 0.706300 0.429000

C -4.032800 -0.696300 0.428400

C -2.788900 -1.118000 1.021400

C -5.231400 1.193400 -0.271800

C -5.775100 2.407000 -0.670700

H -5.292900 3.347500 -0.447900

C -6.990500 2.422700 -1.402300

H -7.422200 3.369900 -1.707100

C -7.655400 1.257300 -1.758000

C -7.099000 0.008900 -1.390400

C -7.657400 -1.238100 -1.759800

C -6.994800 -2.405100 -1.405300

H -7.428000 -3.351200 -1.711400

C -5.779500 -2.392500 -0.673300

H -5.299200 -3.334100 -0.451400

C -5.233700 -1.180300 -0.273100

C -5.919500 0.007400 -0.658000

C -8.915100 1.276100 -2.528000

C -8.916600 -1.253700 -2.530600

N -9.459600 0.011800 -2.853700

O -9.477200 2.303000 -2.877900

O -9.480800 -2.279400 -2.881000

C -2.421300 -2.421400 1.409500

C -3.472900 -3.466800 1.400300

C -4.630400 -3.328100 2.178500

H -4.751600 -2.442100 2.792300

C -5.615600 -4.312200 2.168200

H -6.505000 -4.186200 2.778900

C -5.482100 -5.460300 1.376200

C -4.326600 -5.591500 0.593100

H -4.204200 -6.470000 -0.034000

C -3.334400 -4.615700 0.608200

H -2.444400 -4.733400 -0.000400

C -1.123600 -2.794700 1.802000

N -0.002400 -2.025000 1.551000

C -0.704400 -4.048600 2.384500

C 0.694400 -4.050300 2.384900

C 1.116800 -2.797400 1.802600

C -1.189500 -5.254600 3.072000

C -2.407600 -5.784800 3.457500

H -3.342200 -5.290800 3.234500

C -2.432500 -7.007200 4.180800

H -3.398100 -7.413100 4.467200

C -1.281600 -7.685300 4.535900

C -0.009200 -7.151800 4.187600

C 1.261800 -7.688200 4.536800

C 2.414500 -7.012800 4.182500

H 3.378900 -7.420800 4.469500

C 2.392800 -5.790300 3.459100

H 3.328700 -5.298400 3.236800

C 1.176200 -5.257300 3.072800

C -0.007600 -5.955000 3.457900

C 2.415600 -2.427200 1.410700

C 3.464700 -3.475100 1.402200

C 3.323900 -4.623800 0.610300

H 2.433900 -4.739500 0.001200

C 4.313700 -5.602000 0.596100

H 4.189600 -6.480400 -0.030900

C 5.469000 -5.473400 1.379800

C 5.604900 -4.325500 2.171500

H 6.494300 -4.201500 2.782800

C 4.622100 -3.339000 2.181000

H 4.745100 -2.453100 2.794700

C 2.786400 -1.124900 1.022200

N 2.023300 -0.002500 1.283500

C 4.031400 -0.706300 0.429000

C 4.032800 0.696300 0.428400

C 2.788900 1.118000 1.021400

C 5.231400 -1.193400 -0.271800

C 5.775100 -2.407000 -0.670700

H 5.292900 -3.347500 -0.447900

C 6.990500 -2.422700 -1.402200

H 7.422200 -3.369900 -1.707000

C 7.655400 -1.257300 -1.758000

C 7.099000 -0.008900 -1.390400

C 7.657400 1.238100 -1.759700

C 6.994800 2.405100 -1.405300

H 7.428000 3.351200 -1.711400

C 5.779500 2.392500 -0.673300

H 5.299200 3.334100 -0.451400

C 5.233700 1.180300 -0.273100

C 5.919500 -0.007400 -0.658000

C 8.915100 -1.276100 -2.528000

C 8.916600 1.253700 -2.530600

N 9.459600 -0.011800 -2.853600

O 9.477200 -2.303000 -2.877800

O 9.480800 2.279400 -2.881000

C 2.421300 2.421400 1.409500

C 3.472900 3.466800 1.400300

C 3.334400 4.615700 0.608200

H 2.444300 4.733400 -0.000400

C 4.326600 5.591500 0.593200

H 4.204200 6.470000 -0.034000

C 5.482000 5.460300 1.376200

C 5.615600 4.312200 2.168200

H 6.505000 4.186200 2.778900

C 4.630300 3.328100 2.178500

H 4.751500 2.442100 2.792300

H 1.319700 -8.618000 5.095500

H -1.342100 -8.615000 5.094500

H 1.342000 8.615000 5.094500

H -1.319700 8.618000 5.095500

C 6.521500 -6.552600 1.387700

H 6.621700 -7.018900 0.403400

H 7.497900 -6.156500 1.678900

H 6.262700 -7.346900 2.098300

C -6.537200 -6.536800 1.383000

H -7.512600 -6.138600 1.674800

H -6.638500 -7.001900 0.398300

H -6.280300 -7.332400 2.092900

C -6.521500 6.552600 1.387700

H -6.621700 7.018900 0.403400

H -7.497900 6.156500 1.678900

H -6.262700 7.346900 2.098300

C 6.537200 6.536800 1.383100

H 7.512600 6.138600 1.674800

H 6.638500 7.001900 0.398400

H 6.280300 7.332400 2.092900

C 10.696400 -0.013200 -3.602600

C 10.636900 -0.064700 -5.001400

C 11.908000 0.037800 -2.900700

C 11.844900 -0.066800 -5.705200

C 13.092200 0.039100 -3.643800

C 13.063000 -0.014100 -5.034200

H 11.829400 -0.109900 -6.789800

H 14.045800 0.082600 -3.126900

H 13.992200 -0.014400 -5.596400

C -10.696400 0.013200 -3.602600

C -10.636800 0.064800 -5.001500

C -11.908000 -0.037800 -2.900700

C -11.844900 0.066800 -5.705300

C -13.092200 -0.039100 -3.643900

C -13.063000 0.014100 -5.034300

H -11.829400 0.110000 -6.789800

H -14.045700 -0.082600 -3.127000

H -13.992100 0.014400 -5.596500

C 9.315000 -0.128100 -5.747000

C 9.163300 -1.472800 -6.476500

C 9.151300 1.069000 -6.696300

H 8.509000 -0.066400 -5.010900

H 9.255400 -2.302100 -5.770000

H 8.186000 -1.537100 -6.966800

H 9.933000 -1.594300 -7.246100

H 9.241100 2.010200 -6.146500

H 9.912200 1.062900 -7.483800

H 8.170000 1.041900 -7.181800

C 11.954200 0.106500 -1.383800

C 12.507100 1.463200 -0.917300

C 12.739500 -1.073900 -0.791900

H 10.929200 0.029000 -1.011500

H 11.910300 2.280600 -1.330800

H 12.487900 1.532700 0.175800

H 13.543500 1.600300 -1.243800

H 12.311900 -2.024600 -1.122900

H 13.790500 -1.050700 -1.098300

H 12.712000 -1.042300 0.302500

C -9.315000 0.128100 -5.747000

C -9.163200 1.472800 -6.476500

C -9.151200 -1.068900 -6.696400

H -8.509000 0.066400 -5.011000

H -9.255400 2.302100 -5.770000

H -8.185900 1.537100 -6.966800

H -9.933000 1.594400 -7.246100

H -9.241100 -2.010200 -6.146600

H -9.912100 -1.062800 -7.483800

H -8.169900 -1.041800 -7.181800

C -11.954200 -0.106600 -1.383900

C -12.507100 -1.463300 -0.917300

C -12.739600 1.073800 -0.791900

H -10.929200 -0.029000 -1.011600

H -11.910200 -2.280700 -1.330900

H -12.487800 -1.532800 0.175700

H -13.543500 -1.600400 -1.243900

H -12.312000 2.024600 -1.122900

H -13.790600 1.050500 -1.098400

H -12.712100 1.042200 0.302500

**A**

214

title

Zn -0.452100 0.657900 -0.810600

O 1.951400 2.866200 -0.497900

N -0.357500 2.509400 -0.323300

C 0.799800 3.259500 -0.205400

C 0.419300 4.558500 0.330300

C -0.950100 4.571600 0.533700

C -1.463800 3.265100 0.097500

C 1.022400 5.793000 0.795400

C 2.295700 6.325500 0.855700

C 2.464000 7.614100 1.431100

C 1.402100 8.341600 1.941900

C -1.103800 8.404700 2.419500

C -2.306500 7.725600 2.337300

C -2.427700 6.443500 1.736200

C -1.303200 5.842400 1.197200

C -0.065900 6.548500 1.313900

C 0.080500 7.809200 1.902700

C -2.771100 2.811100 0.113800

C -3.861100 3.783400 0.388800

C -4.644500 3.679500 1.546500

C -5.670300 4.587500 1.793700

C -5.958800 5.617700 0.886700

C -5.175300 5.716100 -0.270200

C -4.139700 4.815600 -0.515700

C -7.100700 6.569600 1.138100

N -2.273200 0.408300 -0.074600

C -3.174200 1.434800 -0.092500

C -4.489800 0.877800 -0.210800

C -4.341100 -0.533500 -0.201100

C -2.945300 -0.808600 -0.100500

C -5.908400 1.201800 -0.421900

C -6.693700 2.332900 -0.599400

C -8.087100 2.190000 -0.825400

C -8.703900 0.948600 -0.900200

C -8.426800 -1.531500 -0.881800

C -7.556700 -2.610700 -0.789200

C -6.168000 -2.442100 -0.561300

C -5.653000 -1.161000 -0.406500

C -6.555800 -0.065600 -0.517800

C -7.915100 -0.218500 -0.757900

C -10.151100 0.803400 -1.147000

C -9.872400 -1.711100 -1.127100

N -10.632900 -0.524700 -1.235800

O -10.914600 1.749500 -1.269700

O -10.406100 -2.805200 -1.233800

C -12.050800 -0.684100 -1.470700

C -12.915100 -0.721200 -0.368500

C -14.282500 -0.872700 -0.617700

C -14.763100 -0.987600 -1.918700

C -13.881000 -0.949700 -2.994500

C -12.506500 -0.794500 -2.790900

C -12.405400 -0.585700 1.056100

C -12.782200 -1.808900 1.906000

C -12.892500 0.729900 1.685200

C -11.555200 -0.759300 -3.974500

C -11.539200 -2.112900 -4.703100

C -11.884700 0.405400 -4.921000

C -2.271400 -2.045300 0.050900

C -3.068000 -3.219500 0.458900

C -3.023300 -4.406700 -0.285600

C -3.787800 -5.506000 0.095200

C -4.594500 -5.464200 1.241400

C -4.615600 -4.281500 1.994700

C -3.877100 -3.169800 1.603800

C -5.438500 -6.647600 1.639100

N -0.084700 -1.255600 -0.738700

C -0.881000 -2.214400 -0.098400

C 0.000600 -3.206100 0.440100

C 1.323400 -2.795100 0.116700

C 1.209900 -1.598400 -0.625900

C 0.082200 -4.316900 1.398800

C -0.804400 -5.103900 2.113500

C -0.293300 -6.049400 3.041900

C 1.061300 -6.212500 3.272100

C 3.417600 -5.408400 2.738500

C 4.198900 -4.519700 2.023800

C 3.647300 -3.602900 1.087500

C 2.281200 -3.602500 0.878100

C 1.480000 -4.509300 1.632100

C 2.003800 -5.417500 2.561900

C 2.266500 -0.623300 -1.158300

O 1.571200 0.590600 -1.474900

C 2.941700 -1.161800 -2.421900

C 2.944700 -0.409000 -3.595300

C 3.607000 -0.877800 -4.732100

C 4.282700 -2.101000 -4.725200

C 4.257700 -2.856500 -3.544200

C 3.595700 -2.399200 -2.410800

C 5.032400 -2.592300 -5.937600

N 2.824500 -0.439300 1.242100

C 3.270600 -0.393200 -0.053500

C 4.646400 -0.198400 0.004000

C 5.014700 -0.180100 1.401300

C 3.860800 -0.351100 2.155800

C 5.875900 -0.098300 -0.797700

C 6.223500 -0.078800 -2.139000

C 7.595600 -0.000600 -2.495500

C 8.609100 0.054400 -1.546400

C 9.216600 0.066500 0.881300

C 8.776300 0.017800 2.200100

C 7.399100 -0.061800 2.534600

C 6.466500 -0.082900 1.510600

C 6.925800 -0.034200 0.164500

C 8.271300 0.035500 -0.172300

C 10.034800 0.122800 -1.921700

C 10.650700 0.138300 0.536700

N 10.953600 0.164800 -0.846400

O 10.430300 0.142700 -3.077800

O 11.545700 0.171000 1.367500

C 12.354500 0.233200 -1.197700

C 12.937300 1.491200 -1.400100

C 14.292200 1.534100 -1.742400

C 15.034100 0.363800 -1.871500

C 14.429800 -0.872300 -1.662000

C 13.076300 -0.961000 -1.323000

C 12.137400 2.776100 -1.272100

C 12.734800 3.704600 -0.203700

C 12.003500 3.472100 -2.636500

C 12.431100 -2.316700 -1.092000

C 12.515500 -3.196200 -2.349100

C 13.035400 -3.006600 0.141700

C 3.668100 -0.699300 3.565200

O 4.617700 -0.788900 4.338000

C 2.270100 -1.098000 3.943000

C 2.067900 -2.359800 4.512600

C 0.777600 -2.832400 4.729600

C -0.342900 -2.058600 4.401300

C -0.134200 -0.780200 3.868600

C 1.154500 -0.303400 3.637200

C -1.735400 -2.601300 4.588300

H 3.152000 5.776400 0.478600

H 3.463400 8.035300 1.476000

H 1.574500 9.319500 2.382000

H -4.440300 2.883200 2.254700

H -6.261400 4.494600 2.700400

H -5.376000 6.509000 -0.984900

H -3.537900 4.907800 -1.413500

H -7.190900 6.812100 2.201000

H -8.054400 6.128300 0.824900

H -6.972700 7.502700 0.583400

H -6.270100 3.326000 -0.584000

H -8.703400 3.073200 -0.955900

H -7.968600 -3.607400 -0.904900

H -5.534900 -3.318100 -0.521900

H -14.976400 -0.900200 0.216600

H -15.828000 -1.106600 -2.094800

H -14.262500 -1.041300 -4.006600

H -11.313500 -0.543300 1.020800

H -12.405500 -2.727200 1.446500

H -13.867700 -1.904700 2.012500

H -12.356400 -1.722900 2.911500

H -13.984600 0.747700 1.766600

H -12.584100 1.582000 1.073600

H -12.479100 0.852700 2.692200

H -10.546100 -0.585800 -3.591500

H -12.520700 -2.343800 -5.130500

H -11.273300 -2.915800 -4.010200

H -10.811600 -2.101400 -5.521800

H -11.866200 1.356200 -4.380700

H -12.877400 0.289500 -5.368300

H -11.155900 0.454500 -5.736900

H -2.392200 -4.455800 -1.166300

H -3.753100 -6.414000 -0.499500

H -5.226000 -4.230700 2.891600

H -3.912800 -2.256600 2.186600

H -5.540600 -6.717400 2.725600

H -5.011700 -7.584800 1.272400

H -6.449200 -6.561800 1.222600

H -1.874400 -5.016600 1.989900

H -1.001700 -6.659400 3.594200

H 1.409000 -6.938400 4.001600

H 3.875500 -6.088600 3.450600

H 5.272100 -4.505600 2.185300

H 4.303800 -2.905900 0.578900

H 2.435500 0.546000 -3.610300

H 3.602400 -0.275300 -5.636200

H 4.767700 -3.815300 -3.512000

H 3.591200 -3.011700 -1.517800

H 6.115300 -2.559000 -5.770200

H 4.813400 -1.981600 -6.817100

H 4.774700 -3.630200 -6.171900

H 1.895100 -0.737900 1.500700

H 5.474400 -0.125200 -2.918900

H 7.879900 0.013500 -3.542300

H 9.525200 0.036900 2.984600

H 7.079700 -0.117000 3.569100

H 14.769200 2.494800 -1.909300

H 16.086200 0.415100 -2.136000

H 15.014600 -1.781300 -1.762400

H 11.128800 2.513900 -0.941800

H 13.742000 4.035700 -0.477600

H 12.797900 3.191200 0.760000

H 12.113100 4.597900 -0.081500

H 11.549300 2.796400 -3.366300

H 12.982100 3.779200 -3.020700

H 11.379500 4.368400 -2.552800

H 11.370100 -2.154200 -0.884700

H 13.554400 -3.425100 -2.608700

H 12.057300 -2.689400 -3.203200

H 11.995700 -4.146700 -2.188100

H 12.933000 -2.368200 1.023400

H 14.100400 -3.216500 -0.004300

H 12.530000 -3.958400 0.337600

H 2.928900 -2.977700 4.741700

H 0.634700 -3.829400 5.134300

H -0.987800 -0.153600 3.625000

H 1.293800 0.693100 3.227100

H -1.800400 -3.236300 5.476500

H -2.028000 -3.213300 3.728900

H -2.468100 -1.795500 4.688700

H -1.058700 9.383500 2.888500

H -3.201800 8.181900 2.748500

H -3.394900 5.965100 1.725800

H 1.949600 1.453000 -1.070000

**A‘**

214

title

Zn -0.355000 1.192600 1.849300

O -2.100000 -1.544500 2.438800

H -0.583800 -1.220500 2.744700

N -2.198400 0.691000 1.728800

C -2.734000 -0.555000 2.007300

C -4.140500 -0.507600 1.639200

C -4.416000 0.743000 1.100500

C -3.185300 1.531600 1.189600

C -5.255300 -1.416600 1.436700

C -5.546000 -2.748000 1.693600

H -4.840300 -3.382800 2.218200

C -6.782400 -3.281400 1.242400

H -7.027400 -4.320700 1.432700

C -7.699600 -2.515200 0.532300

C -8.227800 -0.316200 -0.530100

C -7.799900 0.974900 -0.808700

H -8.434100 1.599600 -1.428300

C -6.567400 1.487900 -0.324500

H -6.283100 2.489600 -0.605700

C -5.755000 0.688400 0.469900

C -6.209300 -0.639100 0.725000

C -7.404900 -1.157800 0.253200

C -2.992400 2.848600 0.798800

C -4.184400 3.638300 0.399900

C -4.305300 4.140300 -0.904100

H -3.510500 3.954800 -1.618900

C -5.426500 4.874600 -1.279800

H -5.504400 5.250300 -2.296000

C -6.453200 5.147500 -0.363700

C -6.318600 4.659700 0.942700

H -7.096500 4.866200 1.672100

C -5.205100 3.910800 1.319400

H -5.116000 3.538200 2.334200

C -7.675800 5.923900 -0.782000

H -7.426800 6.693900 -1.517700

H -8.152700 6.408500 0.073900

H -8.421100 5.263600 -1.241400

N -0.533300 2.844900 0.779600

C -1.715700 3.525800 0.726500

C -1.433500 4.915100 0.507200

C -0.028700 5.034500 0.380400

C 0.521500 3.718600 0.538500

C -2.030100 6.257900 0.464500

C -3.287900 6.814800 0.607400

H -4.163900 6.209700 0.786200

C -3.434400 8.226300 0.536600

H -4.432000 8.642500 0.640300

C -2.361900 9.077300 0.352500

C 0.161000 9.283900 0.120500

C 1.375500 8.623400 0.092100

H 2.292500 9.200000 0.017600

C 1.477200 7.209700 0.165700

H 2.457600 6.757500 0.167400

C 0.320200 6.454300 0.249400

C -0.927000 7.147100 0.293200

C -1.047300 8.543500 0.241300

C -8.969100 -3.079300 0.027900

C -9.502000 -0.855700 -1.054200

N -9.781600 -2.200500 -0.729700

O -9.322200 -4.229800 0.230800

O -10.279300 -0.198100 -1.728600

C -11.020200 -2.745600 -1.241300

C -11.006800 -3.388700 -2.486000

C -12.214300 -3.908100 -2.962300

H -12.234500 -4.414300 -3.922300

C -13.386600 -3.784600 -2.222400

H -14.316100 -4.192900 -2.607800

C -13.369800 -3.139500 -0.989400

H -14.288100 -3.046000 -0.418400

C -12.183800 -2.608600 -0.473400

C -9.734300 -3.534800 -3.302700

H -8.925600 -3.035800 -2.761900

C -9.864300 -2.835700 -4.665000

H -8.922100 -2.898100 -5.219600

H -10.121200 -1.780800 -4.532500

H -10.643400 -3.299000 -5.279100

C -9.340000 -5.013900 -3.444500

H -8.392600 -5.109600 -3.985300

H -10.100000 -5.575200 -3.998100

H -9.228000 -5.475600 -2.459700

C -12.181000 -1.900800 0.870700

H -11.151300 -1.611600 1.097900

C -13.013500 -0.609700 0.809500

H -12.641700 0.047800 0.018800

H -12.964200 -0.073500 1.763300

H -14.066400 -0.827800 0.601900

C -12.645600 -2.837400 1.996700

H -12.022600 -3.735600 2.031100

H -13.684600 -3.151600 1.852900

H -12.582000 -2.332400 2.966300

C 1.862900 3.279900 0.439000

C 2.861100 4.218000 -0.125300

C 3.944800 4.653300 0.649200

H 4.042700 4.300600 1.670100

C 4.878300 5.539300 0.119300

H 5.705000 5.878700 0.736600

C 4.775700 5.991400 -1.204000

C 3.694700 5.542500 -1.975400

H 3.594200 5.882800 -3.001800

C 2.742600 4.677100 -1.443400

H 1.902800 4.349000 -2.046100

C 5.817300 6.910200 -1.789300

H 6.653300 6.336100 -2.206600

H 6.230600 7.579700 -1.030000

H 5.403800 7.519900 -2.597100

N 1.557300 1.122400 1.578500

C 2.335900 2.008700 0.829200

C 3.591200 1.363300 0.602900

C 3.535600 0.104500 1.270800

C 2.254500 0.006600 1.854300

C 4.858900 1.441900 -0.137500

C 5.481300 2.306800 -1.030200

H 5.018500 3.231800 -1.342000

C 6.749500 1.964200 -1.562500

H 7.241100 2.635400 -2.258500

C 7.399400 0.781500 -1.230400

C 7.326200 -1.373600 0.031400

C 6.609700 -2.204700 0.882900

H 7.043000 -3.161500 1.153300

C 5.333500 -1.845100 1.388700

H 4.813800 -2.535900 2.036200

C 4.778200 -0.628800 1.026800

C 5.530800 0.222700 0.170600

C 6.773300 -0.124000 -0.342800

C 1.509100 -1.142500 2.537200

O 0.211000 -0.606900 2.863600

C 2.152400 -1.654500 3.816900

C 1.458000 -2.625000 4.551700

H 0.506700 -2.995600 4.183700

C 1.981500 -3.122500 5.737200

H 1.431900 -3.880100 6.288900

C 3.218400 -2.672900 6.226300

C 3.901300 -1.702700 5.489300

H 4.860200 -1.337400 5.845500

C 3.373600 -1.190600 4.301100

H 3.924900 -0.434700 3.755500

C 3.779600 -3.220300 7.514200

H 3.757600 -4.314900 7.523100

H 3.193900 -2.879300 8.375800

H 4.813400 -2.900400 7.667100

N 0.592400 -1.991400 0.389800

H -0.041900 -1.213200 0.300900

C 1.373800 -2.236400 1.498800

C 2.063200 -3.422300 1.259400

C 1.687200 -3.867500 -0.058300

C 0.760800 -2.974900 -0.579000

C 3.038200 -4.388900 1.801200

C 3.759800 -4.553400 2.968200

H 3.672200 -3.865900 3.797200

C 4.655800 -5.652900 3.076000

H 5.219600 -5.763300 3.997600

C 4.835000 -6.573600 2.060600

C 4.170000 -7.292300 -0.293700

C 3.400100 -7.023900 -1.411800

H 3.474100 -7.681300 -2.272900

C 2.516500 -5.912700 -1.482400

H 1.935700 -5.710700 -2.374700

C 2.425000 -5.080600 -0.385700

C 3.221700 -5.354100 0.763400

C 4.101800 -6.443600 0.848100

C 8.645900 -1.753000 -0.509200

C 8.718100 0.426500 -1.794200

N 9.244800 -0.821200 -1.392600

O 9.214600 -2.798500 -0.234900

O 9.337600 1.148300 -2.561600

C 10.529200 -1.189800 -1.945400

C 10.560900 -1.960500 -3.115300

C 11.812800 -2.309100 -3.631200

H 11.868400 -2.910300 -4.533200

C 12.984700 -1.896800 -3.003700

H 13.948800 -2.174700 -3.419200

C 12.922900 -1.130000 -1.844000

H 13.840800 -0.810800 -1.360300

C 11.692300 -0.764300 -1.290300

C 9.290100 -2.429100 -3.803500

H 8.439300 -1.982500 -3.282100

C 9.228200 -1.947600 -5.261100

H 8.278500 -2.241300 -5.720300

H 10.034900 -2.380200 -5.861900

H 9.315900 -0.858400 -5.311700

C 9.143000 -3.955700 -3.691700

H 9.161600 -4.265300 -2.643500

H 9.958400 -4.468800 -4.212900

H 8.198500 -4.285500 -4.137500

C 11.639600 0.076200 -0.026200

H 10.590100 0.193800 0.256800

C 12.345500 -0.627300 1.143300

H 11.909800 -1.615400 1.316600

H 12.247300 -0.037900 2.061200

H 13.414400 -0.758800 0.945600

C 12.203400 1.483400 -0.282300

H 11.661500 1.967800 -1.099000

H 13.263100 1.440500 -0.555600

H 12.113800 2.104400 0.615600

C 0.138000 -2.952700 -1.898900

O 0.546400 -3.695800 -2.792500

C -0.969800 -1.983100 -2.162600

C -1.011800 -1.337200 -3.404300

H -0.225700 -1.542000 -4.123000

C -2.042600 -0.452200 -3.704700

H -2.049800 0.059500 -4.663300

C -3.083300 -0.224400 -2.795100

C -3.058400 -0.910400 -1.575300

H -3.878700 -0.784000 -0.883300

C -2.006700 -1.759800 -1.243800

H -2.021600 -2.283400 -0.292400

C -4.206500 0.734000 -3.100100

H -4.133900 1.632400 -2.476300

H -5.181400 0.284100 -2.888000

H -4.193600 1.048800 -4.146700

H 0.125200 10.368300 0.067900

H -2.513000 10.152100 0.310500

H 5.534500 -7.395400 2.183400

H 4.836800 -8.149900 -0.283900

**AB**

425

title

Zn 0.005300 1.759300 -2.456700

Zn -0.205500 -1.191400 1.101400

O 2.952900 -0.412700 1.668200

N 0.804900 0.452700 1.482900

C 2.148600 0.552700 1.715100

C 2.437100 1.957500 2.008900

C 1.263100 2.682500 1.856500

C 0.196500 1.702700 1.602600

C 3.482500 2.796500 2.569700

C 4.774100 2.618400 3.026500

C 5.446500 3.722400 3.616600

C 4.865900 4.975200 3.717700

C 2.806100 6.410300 3.259100

C 1.506400 6.437300 2.784300

C 0.842100 5.281700 2.287600

C 1.525700 4.079200 2.250900

C 2.883900 4.082000 2.701000

C 3.542000 5.192400 3.237400

C -1.174300 1.896800 1.670300

C -1.730000 3.269300 1.576200

C -2.374500 3.882600 2.660800

C -2.920900 5.156100 2.530400

C -2.865900 5.847500 1.309900

C -2.227000 5.227600 0.229400

C -1.655900 3.961100 0.364900

C -3.501900 7.206700 1.159700

N -1.707700 -0.412100 2.290600

C -2.120900 0.830100 1.913200

C -3.556000 0.865000 1.959500

C -3.981500 -0.391600 2.464500

C -2.803900 -1.170200 2.668600

C -4.730800 1.622800 1.526500

C -5.000800 2.837200 0.909600

C -6.338800 3.170700 0.589500

C -7.404300 2.320300 0.854900

C -8.162700 0.156500 1.835900

C -7.799200 -1.053900 2.414500

C -6.447000 -1.393900 2.671800

C -5.447000 -0.471900 2.389600

C -5.838900 0.768200 1.809900

C -7.152500 1.088000 1.502200

C -8.786100 2.654600 0.476400

C -9.569100 0.536200 1.605000

N -9.786000 1.765000 0.934300

O -9.085300 3.651200 -0.171900

O -10.519000 -0.141000 1.969100

C -11.156200 2.232400 0.856100

C -11.583400 3.161600 1.816700

C -12.895800 3.636800 1.727400

C -13.749800 3.188800 0.725900

C -13.306100 2.249300 -0.200700

C -12.001700 1.748800 -0.152800

C -10.672400 3.663400 2.926300

C -11.272200 3.386800 4.314000

C -10.340100 5.152300 2.732000

C -11.510400 0.749100 -1.184200

C -12.579400 -0.268100 -1.599700

C -10.944100 1.494400 -2.406200

C -2.644800 -2.482200 3.178000

C -3.764900 -3.076900 3.928900

C -4.264100 -4.339200 3.569500

C -5.388300 -4.862100 4.196400

C -6.021400 -4.165300 5.237300

C -5.482500 -2.933900 5.634800

C -4.383100 -2.385100 4.980200

C -7.268700 -4.714900 5.878900

N -0.528700 -2.928000 2.005900

C -1.470100 -3.235300 2.997900

C -0.946900 -4.329200 3.758200

C 0.324000 -4.638000 3.207700

C 0.517800 -3.756900 2.120600

C -1.131400 -5.123800 4.978200

C -2.094100 -5.255800 5.963900

C -1.853000 -6.148600 7.042500

C -0.689400 -6.889200 7.154500

C 1.595600 -7.424200 6.155200

C 2.499400 -7.167400 5.140800

C 2.218300 -6.265800 4.076600

C 0.990600 -5.631400 4.049700

C 0.066900 -5.894800 5.105700

C 0.328800 -6.771100 6.166600

C 1.651500 -3.628400 1.109300

O 1.609500 -2.301400 0.556300

C 1.448500 -4.640300 -0.013000

C 1.256700 -4.223800 -1.330100

C 1.082100 -5.171600 -2.340600

C 1.101600 -6.542700 -2.065600

C 1.292700 -6.945800 -0.736900

C 1.457100 -6.009200 0.278500

C 0.908600 -7.553700 -3.167800

N 3.129200 -3.150500 2.991900

C 2.975800 -3.786500 1.797400

C 4.235900 -4.219700 1.411200

C 5.170100 -3.785100 2.426600

C 4.446900 -3.109100 3.408700

C 4.980900 -4.828500 0.307800

C 4.693600 -5.373100 -0.933000

C 5.766100 -5.771300 -1.768900

C 7.096200 -5.604400 -1.403700

C 8.719800 -4.805400 0.323600

C 8.906300 -4.216200 1.571600

C 7.818200 -3.847600 2.406600

C 6.525500 -4.091000 1.967300

C 6.348300 -4.715300 0.697400

C 7.405500 -5.066600 -0.131100

C 8.196700 -5.899300 -2.334500

C 9.845900 -5.091600 -0.587300

N 9.498800 -5.617800 -1.860400

O 8.024700 -6.322000 -3.471000

O 11.015100 -4.881200 -0.304400

C 10.587600 -5.853900 -2.783100

C 10.844300 -4.902000 -3.781100

C 11.907100 -5.143800 -4.656200

C 12.684600 -6.291900 -4.536900

C 12.403200 -7.223300 -3.542400

C 11.345300 -7.025400 -2.649500

C 10.001300 -3.647700 -3.938500

C 10.841300 -2.377800 -3.734900

C 9.263300 -3.639900 -5.286700

C 11.050200 -8.059500 -1.576100

C 10.734200 -9.430500 -2.194900

C 12.199700 -8.139600 -0.558500

C 4.834500 -2.273500 4.556400

O 6.009200 -2.107500 4.864700

C 3.675700 -1.512300 5.148000

C 2.484900 -2.149400 5.541500

C 1.307700 -1.418400 5.679000

C 1.284900 -0.035800 5.433800

C 2.498400 0.605200 5.161500

C 3.679600 -0.121100 5.018900

C -0.021700 0.713100 5.389400

O -0.753000 -0.889900 -0.794500

N -1.666100 0.997400 -1.768500

C -1.752600 -0.213800 -1.158400

C -3.155900 -0.573900 -1.066200

C -3.904600 0.424300 -1.673200

C -2.939500 1.436900 -2.146000

C -3.998400 -1.653200 -0.593300

C -3.829200 -2.874800 0.041500

C -4.976700 -3.656400 0.322600

C -6.256900 -3.242100 -0.030200

C -7.684000 -1.517100 -1.135500

C -7.734800 -0.276300 -1.756600

C -6.572800 0.512600 -1.981000

C -5.329200 0.026700 -1.605200

C -5.308900 -1.241000 -0.949100

C -6.432400 -2.008700 -0.698800

C -7.445600 -4.067100 0.253600

C -8.869300 -2.386200 -0.979400

N -8.667500 -3.605600 -0.289500

O -7.397000 -5.113500 0.885800

O -9.968400 -2.109800 -1.433500

C -9.762300 -4.557100 -0.316100

C -10.789400 -4.468700 0.633700

C -11.809600 -5.423000 0.574400

C -11.806600 -6.419300 -0.397700

C -10.782200 -6.474600 -1.336500

C -9.741800 -5.540600 -1.316700

C -10.777200 -3.403100 1.714100

C -12.172800 -2.865100 2.050600

C -10.069700 -3.940100 2.973500

C -8.631300 -5.622000 -2.351900

C -7.788300 -6.892600 -2.155400

C -9.181500 -5.512400 -3.782100

C -3.187500 2.612100 -2.839000

C -4.591300 2.899300 -3.247300

C -5.401900 3.733100 -2.472200

C -6.729900 3.966600 -2.826600

C -7.271400 3.401100 -3.988500

C -6.443900 2.588000 -4.778700

C -5.123900 2.330000 -4.410500

C -8.701500 3.671400 -4.382500

N -0.900900 3.502600 -2.725500

C -2.199100 3.632600 -3.098000

C -2.407400 4.990800 -3.552800

C -1.215600 5.695000 -3.292900

C -0.275900 4.744900 -2.731800

C -3.369200 5.897800 -4.192300

C -4.622000 5.806300 -4.772500

C -5.209300 6.967200 -5.342100

C -4.565700 8.189800 -5.368800

C -2.433100 9.470200 -4.846200

C -1.145400 9.412300 -4.343700

C -0.595000 8.227100 -3.792100

C -1.377200 7.085800 -3.728000

C -2.701600 7.156500 -4.254700

C -3.254200 8.309600 -4.829600

C 0.971600 4.961000 -2.135900

C 1.398300 6.371000 -1.938000

C 0.754300 7.175500 -0.990400

C 1.156900 8.495500 -0.798800

C 2.200400 9.050700 -1.551400

C 2.833200 8.239700 -2.504400

C 2.446900 6.914900 -2.690000

C 2.657500 10.468300 -1.319700

N 1.731400 2.621400 -2.028800

C 1.886700 3.945200 -1.702800

C 3.155600 4.097400 -1.051000

C 3.780500 2.824000 -1.063100

C 2.883600 1.927100 -1.736000

C 4.044800 5.062000 -0.388200

C 4.002000 6.384200 0.029500

C 5.119000 6.943800 0.697300

C 6.256900 6.203700 0.982800

C 7.358100 3.967500 0.957600

C 7.246100 2.616600 0.657100

C 6.112700 2.083200 -0.005500

C 5.093400 2.933000 -0.409600

C 5.209600 4.309100 -0.064200

C 6.300000 4.839100 0.612700

C 7.389000 6.773300 1.737500

C 8.512900 4.509600 1.699100

N 8.455500 5.887400 2.014800

O 7.427900 7.932400 2.123800

O 9.471200 3.834100 2.043800

C 9.578600 6.431500 2.745800

C 10.666100 6.943500 2.026500

C 11.743800 7.457400 2.754600

C 11.729900 7.461900 4.146300

C 10.635100 6.947800 4.835300

C 9.539200 6.421200 4.146300

C 10.704500 6.920400 0.508000

C 10.930300 8.322500 -0.077000

C 11.753700 5.911200 0.011700

C 8.352400 5.853200 4.902300

C 7.655000 6.933400 5.743000

C 8.757000 4.631300 5.741300

C 3.137600 0.610000 -2.239700

C 4.479500 0.025800 -1.965600

C 4.629100 -0.952500 -0.975600

C 5.892000 -1.459500 -0.681600

C 7.027300 -1.036400 -1.387900

C 6.858900 -0.087300 -2.405200

C 5.602700 0.449700 -2.684700

C 8.384800 -1.596500 -1.055700

N 0.949100 0.244800 -3.311100

C 2.265500 -0.147500 -3.029700

C 2.482900 -1.420000 -3.692900

C 1.257100 -1.759900 -4.305200

C 0.346300 -0.708600 -4.030300

C 3.466700 -2.459200 -4.036700

C 4.806700 -2.717600 -3.799500

C 5.400000 -3.880500 -4.358300

C 4.691100 -4.785800 -5.127000

C 2.443400 -5.375500 -6.174800

C 1.123400 -5.003800 -6.370300

C 0.579700 -3.812600 -5.818300

C 1.398600 -3.004800 -5.052200

C 2.754500 -3.392300 -4.855200

C 3.312900 -4.556000 -5.399700

O -1.446500 0.587200 -4.825500

C -1.086000 -0.511200 -4.403100

C -2.045600 -1.612800 -4.167700

C -3.375700 -1.439400 -4.579700

C -4.337100 -2.389300 -4.267400

C -4.001800 -3.542200 -3.541300

C -2.673000 -3.707900 -3.126800

C -1.705800 -2.756200 -3.426000

C -5.046300 -4.576000 -3.217500

H 5.275000 1.661900 2.933800

H 6.455600 3.573300 3.985900

H 5.416000 5.798500 4.161700

H -2.441100 3.354600 3.606500

H -3.408200 5.619500 3.383900

H -2.185400 5.730600 -0.731500

H -1.174700 3.489200 -0.478300

H -3.211800 7.875900 1.976200

H -4.595600 7.134500 1.177600

H -3.216400 7.676200 0.214700

H -4.210500 3.529100 0.657400

H -6.557900 4.120400 0.117600

H -8.580100 -1.757800 2.671000

H -6.225600 -2.368200 3.082600

H -13.249900 4.362700 2.452600

H -14.766400 3.566700 0.670100

H -13.983100 1.896100 -0.970400

H -9.730700 3.110700 2.874100

H -11.486600 2.321400 4.438500

H -12.205700 3.938100 4.465900

H -10.574000 3.693900 5.099900

H -11.243500 5.767800 2.799000

H -9.887600 5.318700 1.751200

H -9.642500 5.495200 3.503900

H -10.698900 0.172600 -0.737800

H -13.381600 0.192900 -2.186200

H -13.025700 -0.742400 -0.720900

H -12.119300 -1.050400 -2.206300

H -10.146700 2.182600 -2.113300

H -11.727100 2.085200 -2.894300

H -10.552300 0.783400 -3.142500

H -3.770000 -4.894300 2.781100

H -5.789600 -5.815800 3.867700

H -5.947500 -2.387500 6.450100

H -3.996900 -1.415700 5.275400

H -7.194400 -5.795000 6.035600

H -8.139600 -4.541400 5.235700

H -7.467100 -4.239200 6.842600

H -3.020100 -4.695800 5.935600

H -2.616400 -6.245400 7.808600

H -0.545500 -7.555500 8.000200

H 1.852300 -8.114500 6.953400

H 3.465300 -7.662700 5.153800

H 2.970100 -6.083100 3.315900

H 1.264500 -3.165800 -1.559800

H 0.943700 -4.839900 -3.361600

H 1.319100 -8.004900 -0.495700

H 1.601000 -6.346500 1.299000

H 1.541100 -8.433800 -3.016800

H 1.146100 -7.121600 -4.143100

H -0.130100 -7.904900 -3.204400

H 2.384000 -2.659200 3.465900

H 3.678000 -5.474900 -1.290700

H 5.554000 -6.185200 -2.746300

H 9.923800 -4.014200 1.889000

H 7.991400 -3.357100 3.358200

H 12.126100 -4.424500 -5.439200

H 13.509300 -6.462300 -5.222400

H 13.011300 -8.118300 -3.456300

H 9.238900 -3.655100 -3.156500

H 11.625600 -2.292800 -4.494000

H 11.323200 -2.384100 -2.752900

H 10.210400 -1.485200 -3.806700

H 8.675100 -4.553600 -5.401800

H 9.966000 -3.578000 -6.124400

H 8.589500 -2.778500 -5.351100

H 10.155800 -7.738800 -1.035200

H 11.597400 -9.836000 -2.732700

H 9.902400 -9.355800 -2.901500

H 10.462300 -10.148700 -1.414200

H 12.383500 -7.158500 -0.114000

H 13.125300 -8.479000 -1.035800

H 11.956400 -8.847800 0.240900

H 2.465300 -3.227000 5.680500

H 0.386800 -1.935800 5.934100

H 2.512000 1.677600 4.995800

H 4.596500 0.380700 4.733000

H 0.137500 1.794500 5.396400

H -0.667700 0.451200 6.233700

H -0.559100 0.457600 4.468000

H -2.846400 -3.233800 0.325300

H -4.877800 -4.611700 0.822100

H -8.695200 0.097600 -2.088200

H -6.691900 1.474700 -2.450200

H -12.617600 -5.382000 1.296100

H -12.608300 -7.151300 -0.425800

H -10.787900 -7.250000 -2.096200

H -10.205600 -2.551800 1.341100

H -12.790300 -3.611900 2.561600

H -12.695400 -2.548700 1.143400

H -12.078300 -1.994600 2.702200

H -9.059800 -4.292400 2.743000

H -10.626800 -4.785000 3.393200

H -10.012900 -3.160500 3.741900

H -7.966200 -4.767900 -2.204700

H -8.393400 -7.792700 -2.306000

H -7.376400 -6.926300 -1.143500

H -6.961100 -6.921700 -2.872600

H -9.762500 -4.593700 -3.904200

H -9.832000 -6.357600 -4.028500

H -8.360700 -5.503300 -4.507600

H -4.981800 4.193400 -1.586800

H -7.364400 4.569500 -2.184900

H -6.844900 2.138900 -5.683100

H -4.491400 1.693700 -5.020800

H -8.754300 4.434900 -5.167900

H -9.283700 4.027800 -3.529900

H -9.185400 2.770900 -4.771500

H -5.161400 4.873400 -4.821800

H -6.199700 6.877300 -5.777700

H -5.045800 9.054400 -5.817700

H -2.815500 10.394900 -5.268800

H -0.521000 10.299800 -4.376900

H 0.427600 8.238500 -3.448600

H -0.050600 6.757400 -0.397400

H 0.652900 9.104900 -0.054300

H 3.643500 8.649400 -3.100400

H 2.952700 6.295900 -3.423300

H 3.517000 10.494400 -0.639300

H 1.866100 11.075700 -0.872900

H 2.970200 10.944500 -2.253400

H 3.123800 6.994000 -0.104100

H 5.085700 7.976500 1.027000

H 8.048100 1.954800 0.965700

H 6.053700 1.017200 -0.150800

H 12.603500 7.855100 2.224600

H 12.575400 7.865300 4.695400

H 10.630500 6.951900 5.920900

H 9.729200 6.576700 0.152600

H 11.908300 8.724700 0.206700

H 10.163400 9.017200 0.277600

H 10.890800 8.290700 -1.170900

H 11.552500 4.917600 0.421000

H 12.761200 6.207900 0.322400

H 11.743500 5.849000 -1.081900

H 7.630100 5.507000 4.161600

H 7.351700 7.772100 5.109700

H 8.316800 7.320500 6.524700

H 6.765400 6.523700 6.234200

H 9.469300 4.904100 6.526900

H 9.225800 3.871900 5.109100

H 7.879200 4.189700 6.225700

H 3.764300 -1.293500 -0.424800

H 5.998400 -2.186600 0.113600

H 7.723800 0.252300 -2.967600

H 5.491400 1.205200 -3.455600

H 8.507500 -2.596400 -1.482000

H 8.517600 -1.699100 0.024200

H 9.188000 -0.969200 -1.448200

H 5.418900 -2.060900 -3.203500

H 6.447700 -4.075200 -4.158300

H 5.188500 -5.668100 -5.519100

H 2.823000 -6.291300 -6.618500

H 0.477400 -5.639600 -6.967600

H -0.462000 -3.563500 -5.989500

H -3.633300 -0.540600 -5.127000

H -5.367700 -2.235300 -4.573600

H -2.394900 -4.578900 -2.540700

H -0.700700 -2.882900 -3.047300

H -4.984200 -5.424100 -3.910000

H -6.053000 -4.159400 -3.294900

H -4.918300 -4.969000 -2.206100

H 3.268200 7.313800 3.646100

H 0.957000 7.373900 2.800300

H -0.181300 5.368100 1.952800

H 2.235800 -1.662300 1.045900

**AB‘**

425

title

Zn -0.541900 0.569200 1.711200

Zn 1.066400 -1.064600 -1.925600

O -2.025900 -2.308900 1.592700

H -2.604400 -0.825800 1.262600

N 0.043300 -1.287300 1.814200

C -0.777300 -2.374800 1.735300

C 0.047000 -3.567300 1.889300

C 1.355800 -3.159300 2.099800

C 1.365400 -1.694900 2.040000

C -0.081400 -4.998700 2.088600

C -1.108900 -5.920000 2.076700

H -2.119500 -5.633300 1.808000

C -0.817400 -7.264600 2.431400

H -1.622400 -7.991000 2.403800

C 0.452400 -7.678400 2.792200

H 0.630900 -8.716400 3.056300

C 2.868100 -6.984300 3.249700

H 3.171800 -7.984500 3.545900

C 3.769000 -5.934300 3.318700

H 4.778600 -6.125500 3.670300

C 3.427200 -4.600900 2.961000

H 4.170800 -3.822800 3.064100

C 2.147200 -4.337100 2.502200

C 1.225400 -5.430800 2.456400

C 1.531000 -6.744800 2.825600

C 2.430800 -0.832100 2.223300

C 3.810400 -1.365700 2.184800

C 4.708000 -1.164000 3.243900

H 4.382400 -0.607200 4.116500

C 6.005800 -1.670400 3.182900

H 6.684900 -1.503300 4.014300

C 6.451000 -2.384500 2.061800

C 5.547900 -2.588100 1.010600

H 5.868900 -3.149100 0.143000

C 4.253500 -2.082800 1.064800

H 3.568900 -2.236300 0.241600

C 7.868100 -2.889500 1.951700

H 8.478800 -2.206600 1.349300

H 7.904100 -3.866500 1.460000

H 8.343700 -2.979800 2.931800

N 1.085900 1.099700 2.894800

C 2.273300 0.587300 2.463200

C 3.231600 1.646100 2.372800

C 2.566400 2.830700 2.791900

C 1.236700 2.455200 3.139100

C 4.558600 2.003400 1.859100

C 5.667900 1.359600 1.322200

H 5.698300 0.285600 1.198700

C 6.782200 2.130400 0.901700

H 7.644800 1.631700 0.472900

C 6.796000 3.520300 0.966800

C 5.566000 5.602500 1.596700

C 4.409700 6.162600 2.127400

H 4.344200 7.244100 2.173500

C 3.333500 5.371000 2.596600

H 2.451500 5.860000 2.986300

C 3.423600 3.987700 2.517200

C 4.608400 3.428300 1.957500

C 5.674500 4.193700 1.509200

C 7.912300 4.322400 0.443200

C 6.688800 6.430000 1.124600

N 7.797800 5.723900 0.594400

O 8.895800 3.837900 -0.105600

O 6.698900 7.651600 1.172900

C 8.941400 6.511300 0.190300

C 10.027100 6.609700 1.073600

C 11.135600 7.357300 0.665000

H 11.992600 7.442400 1.325500

C 11.154200 7.991600 -0.573200

H 12.022800 8.569900 -0.874000

C 10.058900 7.887500 -1.424600

H 10.076300 8.391200 -2.386100

C 8.932700 7.143000 -1.060400

C 10.031000 5.923300 2.429800

H 9.046600 5.473800 2.585300

C 10.248100 6.930800 3.569700

H 10.182400 6.428100 4.540400

H 9.493900 7.722500 3.538200

H 11.234300 7.402200 3.507400

C 11.066500 4.786800 2.463900

H 11.027900 4.260000 3.423400

H 12.081900 5.177200 2.336500

H 10.874200 4.069600 1.662200

C 7.743300 7.056000 -2.001000

H 7.010400 6.374000 -1.562400

C 7.071600 8.432000 -2.142700

H 6.739000 8.790700 -1.166100

H 6.206800 8.380300 -2.812900

H 7.768100 9.166300 -2.560700

C 8.149000 6.470600 -3.362000

H 8.652300 5.509500 -3.233800

H 8.829800 7.137200 -3.901000

H 7.269300 6.314400 -3.996400

C 0.178000 3.224600 3.696000

C 0.506500 4.520100 4.319700

C -0.202400 5.677400 3.964300

H -1.013900 5.599700 3.249700

C 0.129700 6.908900 4.518800

H -0.423400 7.796100 4.224000

C 1.162000 7.022200 5.460200

C 1.850400 5.858400 5.831600

H 2.645800 5.921900 6.568600

C 1.538800 4.627900 5.263800

H 2.087800 3.737900 5.550200

C 1.541300 8.360900 6.039100

H 0.708600 9.067700 5.992000

H 2.379200 8.801700 5.485600

H 1.855700 8.269400 7.082600

N -1.607300 1.846500 2.756800

C -1.162100 2.813000 3.668200

C -2.305600 3.223800 4.431500

C -3.410500 2.484400 3.945000

C -2.922200 1.681300 2.882700

C -2.748700 4.023800 5.578700

C -2.171400 4.888300 6.491600

H -1.119200 5.141300 6.450200

C -2.984200 5.450000 7.512800

H -2.521900 6.131000 8.220900

C -4.330900 5.157600 7.640800

H -4.913100 5.606100 8.440700

C -6.308300 3.810200 6.756100

H -6.988000 4.191700 7.512500

C -6.748200 2.881100 5.831300

H -7.777300 2.537800 5.873600

C -5.898300 2.343400 4.825300

H -6.282200 1.586400 4.149200

C -4.589300 2.779600 4.755800

C -4.139300 3.725700 5.725200

C -4.954400 4.255500 6.733000

C -3.645800 0.868900 1.804700

O -2.652600 0.154400 1.046400

C -4.260500 1.936200 0.902200

C -3.477800 2.432600 -0.146000

H -2.551600 1.933600 -0.400400

C -3.868300 3.580900 -0.827200

H -3.245200 3.960500 -1.630100

C -5.042900 4.265100 -0.498400

C -5.838100 3.739700 0.528900

H -6.756100 4.246900 0.811800

C -5.445700 2.599800 1.231500

H -6.050800 2.254800 2.061000

C -5.408100 5.544600 -1.207200

H -5.628300 6.343200 -0.491800

H -6.293500 5.432400 -1.837100

H -4.593700 5.879200 -1.854200

N -3.866300 -0.932700 3.376000

H -2.874300 -0.850000 3.544000

C -4.535500 -0.154900 2.460700

C -5.785000 -0.741100 2.280600

C -5.804300 -1.942000 3.093400

C -4.589200 -2.045200 3.756300

C -7.068700 -0.660900 1.560100

C -7.699600 0.196600 0.672500

H -7.226300 1.109000 0.334600

C -8.983400 -0.140700 0.161600

H -9.460600 0.517000 -0.558500

C -9.630300 -1.318600 0.512400

C -9.562700 -3.442300 1.833800

C -8.862600 -4.246300 2.728300

H -9.308600 -5.193400 3.012300

C -7.602300 -3.867200 3.257400

H -7.066000 -4.512100 3.943900

C -7.052300 -2.657400 2.862500

C -7.770600 -1.844700 1.941300

C -9.011300 -2.202200 1.429400

C -10.934600 -1.699800 -0.064300

C -10.855000 -3.861700 1.255300

N -11.433800 -2.966400 0.325300

O -11.560500 -1.000900 -0.845600

O -11.408300 -4.915900 1.528900

C -12.660100 -3.395900 -0.309100

C -12.573900 -4.087100 -1.526300

C -13.767400 -4.481900 -2.136900

H -13.733200 -5.010900 -3.084000

C -14.997300 -4.202800 -1.547100

H -15.915500 -4.517800 -2.034000

C -15.052300 -3.520300 -0.335800

H -16.014800 -3.306100 0.118200

C -13.882500 -3.101200 0.305900

C -11.235000 -4.368300 -2.187500

H -10.452300 -4.138800 -1.461000

C -11.072900 -5.847200 -2.565700

H -10.070200 -6.029300 -2.967700

H -11.792500 -6.151700 -3.332400

H -11.215900 -6.489400 -1.691900

C -11.024000 -3.439900 -3.396100

H -11.118500 -2.392000 -3.099800

H -11.768200 -3.639600 -4.174500

H -10.030700 -3.593200 -3.832900

C -13.955600 -2.347700 1.622200

H -12.933600 -2.119300 1.936000

C -14.686200 -1.006700 1.448800

H -14.198100 -0.402400 0.679100

H -14.684400 -0.443600 2.388200

H -15.729500 -1.156900 1.151500

C -14.584800 -3.216900 2.722200

H -14.024200 -4.148400 2.839300

H -15.622800 -3.472000 2.483500

H -14.583400 -2.685600 3.680000

C -4.022600 -3.135400 4.559500

O -4.670300 -4.166600 4.738700

C -2.601500 -3.005800 5.014600

C -2.014600 -1.793900 5.423900

H -2.626500 -0.911000 5.580000

C -0.646500 -1.717700 5.661600

H -0.207600 -0.766300 5.948200

C 0.176100 -2.845200 5.520500

C -0.427400 -4.064300 5.194400

H 0.187600 -4.952200 5.090200

C -1.794200 -4.146200 4.944400

H -2.245300 -5.085900 4.648300

C 1.667800 -2.729800 5.697600

H 2.153000 -3.706700 5.645200

H 1.920800 -2.266300 6.657300

H 2.096600 -2.104200 4.907500

O -0.140900 1.324600 -0.065900

N 1.791000 0.612000 -1.129900

C 1.017200 1.543200 -0.519900

C 1.687800 2.828400 -0.587800

C 2.798700 2.691300 -1.402000

C 2.919100 1.246200 -1.684800

C 1.467800 4.211900 -0.222600

C 0.534800 4.909900 0.517300

H -0.273400 4.399700 1.030200

C 0.651300 6.324500 0.580400

H -0.066400 6.878500 1.176000

C 1.644000 7.017200 -0.094100

C 3.593300 6.885800 -1.734300

C 4.346400 6.059600 -2.548900

H 5.070000 6.500800 -3.224300

C 4.215200 4.644900 -2.546900

H 4.816900 4.065400 -3.231300

C 3.314100 4.049900 -1.683200

C 2.493000 4.925000 -0.903700

C 2.596100 6.318700 -0.892500

C -8.083900 2.334800 -3.090200

C -6.874600 4.270500 -4.172500

N -8.028600 3.627300 -3.657500

O -9.145900 1.867700 -2.703400

O -6.947000 5.399200 -4.633700

C -9.281000 4.348500 -3.740400

C -9.799600 4.949300 -2.582600

C -11.014900 5.631600 -2.692500

H -11.444100 6.098100 -1.811600

C -11.683000 5.715600 -3.910000

H -12.624200 6.253000 -3.976400

C -11.150600 5.103100 -5.039700

H -11.680500 5.163900 -5.984900

C -9.944400 4.399000 -4.975000

C -9.108200 4.850400 -1.232100

H -8.137600 4.370900 -1.382200

C -8.847300 6.237900 -0.625600

H -8.282400 6.148600 0.308600

H -9.784500 6.753000 -0.392900

H -8.278100 6.870500 -1.312100

C -9.911100 3.961300 -0.266700

H -9.359900 3.814600 0.669200

H -10.105800 2.985900 -0.716100

H -10.873500 4.421700 -0.019300

C -9.402700 3.709000 -6.216200

H -8.486400 3.180900 -5.939800

C -10.389800 2.649900 -6.733600

H -9.962600 2.114500 -7.588100

H -11.329300 3.105500 -7.062500

H -10.625200 1.922800 -5.951000

C -9.033700 4.731300 -7.302900

H -8.308600 5.452100 -6.917400

H -9.917300 5.281600 -7.643400

H -8.599100 4.226700 -8.172500

C 3.936400 0.549300 -2.304100

C 5.100700 1.237700 -2.906400

C 5.874300 2.156200 -2.189500

H 5.585000 2.412900 -1.182700

C 7.029400 2.708700 -2.740700

H 7.643700 3.371300 -2.140400

C 7.438800 2.367700 -4.036200

C 6.647500 1.466900 -4.763800

H 6.946000 1.188200 -5.770500

C 5.502400 0.904300 -4.210100

H 4.913400 0.194100 -4.781100

C 8.695900 2.948700 -4.630300

H 9.426000 3.187700 -3.852600

H 8.483000 3.876400 -5.174700

H 9.159800 2.254200 -5.336300

N 2.909000 -1.717900 -2.249100

C 3.983800 -0.902600 -2.337100

C 5.176000 -1.712100 -2.364900

C 4.768500 -3.058600 -2.320300

C 3.326800 -3.049000 -2.155100

C 6.636200 -1.637600 -2.210800

C 7.607600 -0.663700 -2.026100

H 7.369800 0.387100 -2.050800

C 8.950100 -1.047100 -1.774800

H 9.702200 -0.283100 -1.608900

C 9.339600 -2.378100 -1.720400

C 8.651500 -4.770400 -1.869600

C 7.622100 -5.677900 -2.075900

H 7.859200 -6.735900 -2.050400

C 6.282200 -5.263500 -2.280100

H 5.529200 -6.016700 -2.438100

C 5.969300 -3.911700 -2.263600

C 7.054200 -2.995500 -2.152900

C 8.368300 -3.386000 -1.928800

C 2.491900 -4.098800 -1.745600

C 3.144300 -5.429500 -1.565800

C 3.811200 -5.718900 -0.369500

H 3.840300 -4.979300 0.419700

C 4.422300 -6.956600 -0.185000

H 4.919800 -7.163400 0.757200

C 4.390900 -7.935700 -1.188300

C 3.719700 -7.637100 -2.381500

H 3.673600 -8.382800 -3.169500

C 3.101600 -6.399900 -2.571200

H 2.576000 -6.189600 -3.496700

C 5.078800 -9.262500 -0.990200

H 4.713600 -10.012100 -1.696800

H 4.922600 -9.642600 0.023800

H 6.161700 -9.170000 -1.136100

N 0.331000 -2.893400 -1.622000

C 1.085200 -4.034500 -1.478500

C 0.193700 -5.143000 -1.231800

C -1.121000 -4.648500 -1.382200

C -1.009100 -3.229300 -1.609800

C 0.128700 -6.583300 -0.944400

C 0.999500 -7.582900 -0.556100

H 2.039000 -7.388100 -0.365100

C 0.511900 -8.900400 -0.359100

H 1.223100 -9.668600 -0.071100

C -0.820100 -9.227800 -0.511500

C -3.163500 -8.380700 -0.981500

C -3.955200 -7.291400 -1.290100

H -5.026600 -7.424000 -1.404400

C -3.425700 -5.986000 -1.455900

H -4.106300 -5.190700 -1.699200

C -2.064500 -5.776300 -1.310000

C -1.253800 -6.917400 -1.034500

C -1.759700 -8.212900 -0.841000

C 10.723600 -2.779200 -1.397800

C 10.027600 -5.204500 -1.549300

N 10.960100 -4.171900 -1.311300

O 11.624400 -1.983100 -1.185800

O 10.357500 -6.377600 -1.462900

C 12.272100 -4.576200 -0.857900

C 13.265900 -4.855500 -1.803000

C 14.521400 -5.248400 -1.328700

H 15.311800 -5.474200 -2.037700

C 14.769200 -5.352100 0.036800

H 15.750500 -5.657100 0.387700

C 13.762300 -5.064500 0.954100

H 13.964000 -5.144700 2.017800

C 12.491900 -4.671700 0.523600

C 13.005000 -4.746900 -3.294800

H 11.978900 -4.394400 -3.430900

C 13.930900 -3.708200 -3.946800

H 13.817800 -2.734800 -3.461300

H 13.693700 -3.596700 -5.010100

H 14.982000 -4.005300 -3.869700

C 13.108100 -6.123500 -3.970900

H 12.417500 -6.830600 -3.503500

H 14.121100 -6.531200 -3.887000

H 12.864900 -6.048500 -5.036200

C 11.402000 -4.352500 1.533700

H 10.491500 -4.104800 0.982600

C 11.772500 -3.117600 2.370400

H 10.960000 -2.860100 3.058400

H 11.963900 -2.259300 1.720700

H 12.672100 -3.298500 2.967800

C 11.075900 -5.572000 2.409500

H 10.243100 -5.347200 3.084700

H 11.933300 -5.864900 3.024100

H 10.798400 -6.428000 1.787400

C -2.054600 -2.305900 -1.896900

C -3.462100 -2.782000 -1.776500

C -4.052300 -2.998900 -0.523600

H -3.458500 -2.874800 0.371800

C -5.389400 -3.383700 -0.435000

H -5.834400 -3.527400 0.544100

C -6.164400 -3.594400 -1.583300

C -5.550900 -3.420900 -2.833500

H -6.125400 -3.592700 -3.739100

C -4.224700 -3.004600 -2.932200

H -3.774500 -2.850500 -3.907300

C -7.624100 -3.951800 -1.475500

H -8.244700 -3.050400 -1.539000

H -7.849600 -4.431600 -0.520000

H -7.937600 -4.618900 -2.283300

N -0.637600 -0.472300 -2.765200

C -1.877100 -1.001200 -2.401400

C -2.878400 -0.022600 -2.740000

C -2.202300 1.060200 -3.352100

C -0.818800 0.742400 -3.318400

C -4.310200 0.291400 -2.688600

C -5.459300 -0.253300 -2.139800

H -5.440800 -1.153100 -1.549400

C -6.699900 0.399500 -2.334400

H -7.603900 -0.059400 -1.959600

C -6.798700 1.626200 -2.970900

C -5.612300 3.504200 -4.097900

C -4.405200 4.010100 -4.567100

H -4.409700 4.987500 -5.037400

C -3.183400 3.301100 -4.424700

H -2.268100 3.751800 -4.786800

C -3.181300 2.066100 -3.788400

C -4.430900 1.545200 -3.355900

C -5.629300 2.231800 -3.484700

O 1.354600 0.867200 -4.204200

C 0.367100 1.489400 -3.810800

C 0.345800 2.971900 -3.795000

C 1.211400 3.670900 -4.649800

H 1.839800 3.103000 -5.327100

C 1.266800 5.056700 -4.609200

H 1.942200 5.589000 -5.272700

C 0.480100 5.784800 -3.702300

C -0.359700 5.078900 -2.833900

H -0.937900 5.619800 -2.091900

C -0.429500 3.690600 -2.875100

H -1.048400 3.161700 -2.160800

C 0.546900 7.288600 -3.665100

H -0.035200 7.728400 -4.483900

H 1.578600 7.635200 -3.770400

H 0.152000 7.677800 -2.724000

H 3.741800 7.961100 -1.759100

H 1.690500 8.100500 -0.030100

H -1.160300 -10.248000 -0.358500

H -3.605700 -9.363900 -0.848800

**A’B**

425

title

Zn -1.588200 0.343900 0.245300

Zn 0.941900 -2.789300 2.047400

O 1.274900 1.823400 0.698800

H -0.103700 1.957500 1.474100

N 0.251500 0.043400 -0.388300

C 1.323000 0.821600 -0.057300

C 2.484200 0.292500 -0.750700

C 2.085300 -0.809800 -1.497200

C 0.641800 -0.956400 -1.282800

C 3.848800 0.664200 -1.055500

C 4.719800 1.684800 -0.713700

H 4.440100 2.445200 0.005900

C 6.000800 1.720600 -1.322000

H 6.703800 2.501700 -1.057200

C 6.391900 0.777600 -2.264000

C 5.757800 -1.189800 -3.668200

C 4.766900 -2.103900 -4.009600

H 4.975600 -2.805300 -4.810400

C 3.496700 -2.127900 -3.370700

H 2.763500 -2.849100 -3.702300

C 3.220800 -1.214600 -2.358300

C 4.257000 -0.289800 -2.029000

C 5.492100 -0.247100 -2.649400

C -0.234700 -1.824500 -1.917000

C 0.322500 -2.981900 -2.655800

C 0.102800 -3.165000 -4.029900

H -0.490800 -2.441000 -4.576500

C 0.635700 -4.268900 -4.688900

H 0.459000 -4.391200 -5.753900

C 1.385100 -5.235800 -3.999700

C 1.595700 -5.052300 -2.627400

H 2.167800 -5.784300 -2.065300

C 1.080100 -3.936400 -1.968500

H 1.247700 -3.803400 -0.910100

C 1.954300 -6.431100 -4.721300

H 1.193500 -6.926000 -5.333800

H 2.356500 -7.164500 -4.017900

H 2.766400 -6.135700 -5.395900

N -2.230000 -0.451000 -1.558600

C -1.665400 -1.626900 -1.952800

C -2.692100 -2.474100 -2.498700

C -3.885300 -1.719000 -2.498000

C -3.570600 -0.447200 -1.912700

C -2.998700 -3.847000 -2.915800

C -2.317700 -5.045000 -3.028900

H -1.261300 -5.117100 -2.812200

C -3.029700 -6.206100 -3.430300

H -2.481600 -7.138200 -3.518800

C -4.386300 -6.195100 -3.701100

C -6.523500 -4.811500 -3.774800

C -7.100700 -3.574800 -3.553200

H -8.163600 -3.447200 -3.710400

C -6.358000 -2.448300 -3.113700

H -6.880900 -1.525400 -2.912700

C -4.992700 -2.573100 -2.941700

C -4.402600 -3.850200 -3.183300

C -5.124500 -4.985200 -3.575600

C 7.716600 0.818900 -2.897800

C 7.049700 -1.111500 -4.385600

N 7.946700 -0.112600 -3.941400

O 8.582000 1.621200 -2.582400

O 7.341000 -1.847100 -5.316600

C 9.163900 0.090700 -4.700000

C 9.203800 1.176600 -5.590800

C 10.401100 1.421700 -6.268400

H 10.462400 2.261500 -6.953200

C 11.512200 0.607100 -6.074400

H 12.436800 0.814300 -6.604600

C 11.435900 -0.477400 -5.207800

H 12.301000 -1.118200 -5.074300

C 10.261400 -0.757900 -4.501000

C 8.002100 2.076700 -5.838200

H 7.156600 1.687900 -5.264400

C 7.584400 2.044800 -7.317700

H 6.679800 2.642300 -7.471600

H 7.382400 1.020100 -7.643500

H 8.367400 2.454800 -7.963500

C 8.264600 3.511700 -5.352000

H 7.379300 4.137100 -5.508400

H 9.097100 3.967200 -5.898400

H 8.511400 3.517100 -4.288000

C 10.200500 -1.982700 -3.603100

H 9.251200 -1.954700 -3.058300

C 10.206400 -3.259200 -4.463500

H 9.375000 -3.248000 -5.171100

H 10.123500 -4.150200 -3.833500

H 11.140900 -3.338200 -5.029000

C 11.333800 -1.994900 -2.565700

H 11.314200 -1.103100 -1.936500

H 12.314300 -2.055700 -3.048500

H 11.244700 -2.873200 -1.915700

C -4.380200 0.694300 -1.720600

C -5.669800 0.766400 -2.436700

C -6.863000 0.951200 -1.721700

H -6.817200 1.088900 -0.647600

C -8.089400 0.940400 -2.376000

H -9.004800 1.042200 -1.801000

C -8.160600 0.792200 -3.769600

C -6.962200 0.663800 -4.484800

H -6.994900 0.564600 -5.565700

C -5.733700 0.631700 -3.829600

H -4.815900 0.499100 -4.391900

C -9.495700 0.739700 -4.466300

H -10.181200 1.496300 -4.072900

H -9.970900 -0.236800 -4.314500

H -9.392500 0.896500 -5.542800

N -3.019800 1.692200 0.073800

C -4.021900 1.783600 -0.898000

C -4.475400 3.138500 -0.911500

C -3.694500 3.840500 0.049400

C -2.837800 2.890000 0.645800

C -5.313500 4.107900 -1.631800

C -6.238600 4.102300 -2.668900

H -6.532500 3.187000 -3.164100

C -6.806900 5.328100 -3.100400

H -7.530900 5.331700 -3.908100

C -6.461100 6.548000 -2.531900

C -5.040900 7.767700 -0.876800

C -4.053000 7.689000 0.097000

H -3.692100 8.614900 0.531600

C -3.505000 6.449300 0.517900

H -2.702800 6.446700 1.247800

C -3.980600 5.271200 -0.040900

C -4.976100 5.369800 -1.056600

C -5.509400 6.577000 -1.485000

C -1.828300 3.012200 1.789000

O -1.045200 1.805300 1.800900

C -2.617800 3.088300 3.090000

C -2.803000 1.927800 3.846400

H -2.305000 1.015000 3.544400

C -3.614000 1.952900 4.978300

H -3.750400 1.044700 5.555400

C -4.242900 3.130500 5.395700

C -4.061300 4.283400 4.622400

H -4.545600 5.210400 4.917700

C -3.268300 4.262500 3.477500

H -3.154200 5.166700 2.893000

C -5.062800 3.165600 6.659800

H -4.470000 3.558700 7.494800

H -5.396100 2.164700 6.945200

H -5.942000 3.808200 6.552700

N -0.398000 4.145800 0.213600

H -0.611600 3.418600 -0.452000

C -0.896100 4.158200 1.490900

C -0.175600 5.123300 2.190800

C 0.800200 5.665600 1.272200

C 0.656700 5.022900 0.047000

C 0.009700 5.785500 3.492500

C -0.557400 5.713400 4.748400

H -1.369700 5.032600 4.966700

C -0.050200 6.556000 5.778200

H -0.523100 6.511400 6.755000

C 1.008900 7.423700 5.585200

C 2.760400 8.301900 3.949300

C 3.275100 8.246500 2.665000

H 4.138400 8.856200 2.416600

C 2.716900 7.418600 1.652500

H 3.142900 7.381500 0.656100

C 1.619200 6.645900 1.972600

C 1.098900 6.692900 3.299900

C 1.635800 7.504700 4.309200

C -7.040900 7.823200 -2.999500

C -5.602100 9.057600 -1.321300

N -6.570000 8.988600 -2.353300

O -7.877900 7.897700 -3.887000

O -5.274400 10.136700 -0.851500

C -7.127700 10.245600 -2.800100

C -8.311200 10.705400 -2.207800

C -8.835200 11.921200 -2.657100

H -9.753500 12.300000 -2.219300

C -8.195300 12.649400 -3.655600

H -8.614400 13.593300 -3.991500

C -7.018900 12.170100 -4.224000

H -6.523400 12.744400 -5.000500

C -6.463300 10.956200 -3.808500

C -9.024300 9.916900 -1.122700

H -8.413800 9.041400 -0.885900

C -9.158200 10.736300 0.170000

H -9.619300 10.133700 0.959700

H -9.783600 11.622500 0.020300

H -8.176100 11.070300 0.516700

C -10.382700 9.402600 -1.626800

H -10.250900 8.799800 -2.529500

H -11.054500 10.233300 -1.867900

H -10.870400 8.787700 -0.862600

C -5.174500 10.449400 -4.432500

H -4.953800 9.469000 -4.001900

C -5.326800 10.251800 -5.948600

H -6.155700 9.572200 -6.166400

H -4.410000 9.830000 -6.374000

H -5.523900 11.200500 -6.458700

C -3.998000 11.375300 -4.083200

H -3.897900 11.472000 -2.998800

H -4.145300 12.376500 -4.502000

H -3.061000 10.977300 -4.487200

C 1.497600 5.014600 -1.150500

O 2.508100 5.712800 -1.221300

C 1.164300 3.987800 -2.196800

C -0.147900 3.683300 -2.599100

H -0.975300 4.317000 -2.293600

C -0.402800 2.565500 -3.388500

H -1.427400 2.322100 -3.654400

C 0.640000 1.734400 -3.820000

C 1.951300 2.092900 -3.486900

H 2.775500 1.468700 -3.818600

C 2.212800 3.204700 -2.690800

H 3.227600 3.448300 -2.398700

C 0.349900 0.496700 -4.628800

H -0.585700 0.035000 -4.301300

H 1.149200 -0.240900 -4.523600

H 0.251300 0.732600 -5.695500

O -1.785100 -1.041600 1.670000

N -0.865200 -3.139900 1.314000

C -1.891100 -2.248300 1.316300

C -3.094300 -2.929400 0.876100

C -2.783400 -4.259600 0.645000

C -1.345600 -4.404000 0.949600

C -4.496900 -2.661300 0.624600

C -5.355600 -1.574500 0.692900

H -4.999900 -0.592400 0.984900

C -6.722000 -1.767500 0.369400

H -7.417300 -0.938500 0.417600

C -7.220300 -3.008200 -0.015700

C -6.753200 -5.419800 -0.469200

C -5.797900 -6.424400 -0.537000

H -6.117500 -7.413100 -0.847300

C -4.431100 -6.200800 -0.218000

H -3.740600 -7.021500 -0.316400

C -4.019900 -4.943300 0.198100

C -5.016100 -3.921000 0.227300

C -6.348300 -4.119300 -0.089800

C -8.648100 -3.212400 -0.322100

C -8.183700 -5.660400 -0.747500

N -9.044100 -4.545600 -0.579900

O -9.471500 -2.307300 -0.317800

O -8.635000 -6.746600 -1.073800

C -10.466200 -4.819300 -0.529100

C -11.210800 -4.897300 -1.712900

C -12.582300 -5.145500 -1.607700

H -13.184400 -5.204800 -2.508200

C -13.185000 -5.314700 -0.364500

H -14.252100 -5.505100 -0.300700

C -12.421700 -5.240900 0.796200

H -12.898200 -5.373300 1.762400

C -11.046400 -4.995700 0.737100

C -10.561000 -4.666500 -3.063400

H -9.486000 -4.789300 -2.925100

C -10.993400 -5.691200 -4.119600

H -10.424200 -5.541900 -5.043200

H -12.054300 -5.597900 -4.373400

H -10.814500 -6.709900 -3.765000

C -10.817600 -3.220800 -3.529000

H -10.312900 -3.021600 -4.481800

H -10.462500 -2.505500 -2.781300

H -11.889000 -3.048800 -3.676300

C -10.234300 -4.895400 2.019000

H -9.178800 -4.823700 1.746700

C -10.593900 -3.613800 2.790200

H -9.969100 -3.513400 3.684200

H -11.640500 -3.632100 3.112000

H -10.449200 -2.732000 2.160800

C -10.383000 -6.148600 2.894400

H -10.114900 -7.049500 2.334900

H -11.408800 -6.269700 3.256500

H -9.730000 -6.078800 3.770900

C -0.571700 -5.549300 0.906000

C -1.253400 -6.836000 0.592200

C -1.167600 -7.402300 -0.684800

H -0.583700 -6.901200 -1.448400

C -1.808800 -8.605900 -0.966900

H -1.729100 -9.034700 -1.961700

C -2.545200 -9.282900 0.015100

C -2.616200 -8.715900 1.294600

H -3.175200 -9.225600 2.074200

C -1.983900 -7.506100 1.579600

H -2.042600 -7.074600 2.571800

C -3.260700 -10.570600 -0.306500

H -2.696200 -11.171700 -1.025000

H -4.244200 -10.373000 -0.749600

H -3.422400 -11.172700 0.591500

N 1.613300 -4.453500 1.210700

C 0.866000 -5.575200 1.034400

C 1.755900 -6.680600 0.776900

C 3.053500 -6.145100 0.649800

C 2.946400 -4.723000 0.918600

C 1.817400 -8.139400 0.625800

C 0.957800 -9.213100 0.775800

H -0.072800 -9.082200 1.065600

C 1.446900 -10.529400 0.569100

H 0.751400 -11.356200 0.676600

C 2.765600 -10.789000 0.247800

H 3.105400 -11.809700 0.098200

C 5.087200 -9.822600 -0.103700

H 5.530400 -10.799100 -0.276800

C 5.874500 -8.686300 -0.104300

H 6.941900 -8.777000 -0.280600

C 5.345700 -7.391800 0.133000

H 6.023300 -6.556700 0.153700

C 3.986600 -7.240500 0.349300

C 3.184200 -8.421200 0.340200

C 3.691600 -9.714300 0.143900

C 3.919600 -3.719000 0.835200

C 5.241300 -4.122400 0.282000

C 5.394900 -4.321600 -1.094600

H 4.546300 -4.171800 -1.752400

C 6.634100 -4.691100 -1.617800

H 6.743800 -4.824100 -2.690000

C 7.739900 -4.892000 -0.780200

C 7.572200 -4.698000 0.598800

H 8.416400 -4.848300 1.265200

C 6.343300 -4.309800 1.125000

H 6.230400 -4.153000 2.192400

C 9.082800 -5.283100 -1.339000

H 9.743400 -4.412200 -1.409200

H 8.990400 -5.707200 -2.341500

H 9.581700 -6.016600 -0.698800

N 2.701400 -1.869400 1.950700

C 3.798500 -2.361200 1.279800

C 4.832700 -1.366600 1.328600

C 4.361100 -0.317200 2.158700

C 3.020200 -0.667500 2.545700

C 6.189800 -1.044000 0.864800

C 7.122400 -1.549300 -0.030200

H 6.917000 -2.418000 -0.632200

C 8.373100 -0.904000 -0.188500

H 9.112600 -1.327600 -0.857600

C 8.682000 0.273100 0.475400

C 7.884300 2.106400 1.954300

C 6.861300 2.602000 2.749600

H 7.005400 3.563400 3.230500

C 5.642000 1.901600 2.940700

H 4.893700 2.351200 3.568900

C 5.435700 0.682900 2.307400

C 6.506300 0.182200 1.509700

C 7.709200 0.855500 1.319400

C 9.993800 0.938200 0.331800

C 9.146700 2.848600 1.740100

N 10.128900 2.193500 0.961800

O 10.932900 0.444500 -0.272800

O 9.360000 3.950500 2.220600

C 11.425900 2.833200 0.874000

C 12.288500 2.732900 1.976700

C 13.528300 3.372900 1.896200

H 14.211300 3.318700 2.738300

C 13.897400 4.073500 0.751800

H 14.864800 4.565000 0.704900

C 13.031500 4.136700 -0.334600

H 13.330100 4.671500 -1.230500

C 11.777300 3.517600 -0.298100

C 11.918900 1.956100 3.230500

H 10.943100 1.490100 3.069000

C 12.917200 0.816500 3.491100

H 12.981800 0.152400 2.624100

H 12.606200 0.225400 4.359200

H 13.921300 1.202700 3.694200

C 11.785500 2.890900 4.443400

H 11.048900 3.672600 4.242600

H 12.741100 3.372800 4.675800

H 11.469900 2.329200 5.329500

C 10.867100 3.577100 -1.512100

H 9.938200 3.057900 -1.277400

C 11.501600 2.842200 -2.705000

H 10.815000 2.826600 -3.553800

H 11.726100 1.807200 -2.439200

H 12.431500 3.329100 -3.020500

C 10.497700 5.025900 -1.867400

H 9.795000 5.040800 -2.707000

H 11.378400 5.607200 -2.161400

H 10.030500 5.531900 -1.016800

C 2.171500 -0.019700 3.508900

C 2.627000 1.283000 4.078200

C 2.473100 2.487200 3.379000

H 2.061300 2.474900 2.380000

C 2.876100 3.690200 3.956800

H 2.746700 4.612200 3.401400

C 3.458600 3.727500 5.230700

C 3.630700 2.513900 5.911400

H 4.090700 2.513100 6.895400

C 3.211000 1.308500 5.351700

H 3.337700 0.380800 5.900200

C 3.859300 5.039300 5.854200

H 3.006100 5.494800 6.369500

H 4.187300 5.755400 5.096300

H 4.662700 4.907600 6.584200

N 0.563800 -1.870300 3.763700

C 1.016600 -0.577900 4.071200

C 0.132900 -0.050900 5.095800

C -0.797300 -1.072600 5.383000

C -0.502200 -2.158500 4.519600

C -0.173400 1.096800 5.959800

C 0.243500 2.409200 6.090300

H 0.994800 2.839000 5.446800

C -0.354600 3.227300 7.085200

H 0.007200 4.243700 7.184800

C -1.369100 2.781900 7.912000

H -1.798600 3.444000 8.658500

C -2.948600 0.867500 8.481800

H -3.458500 1.437200 9.253000

C -3.360500 -0.420000 8.182200

H -4.192200 -0.851600 8.730200

C -2.737500 -1.200100 7.171200

H -3.110000 -2.194800 6.952400

C -1.673700 -0.657400 6.474800

C -1.247600 0.661200 6.797900

C -1.866800 1.456700 7.769700

O -0.432800 -4.475900 4.146400

C -1.147300 -3.491400 4.330300

C -2.624800 -3.589900 4.266800

C -3.192000 -4.869200 4.160900

H -2.536000 -5.726400 4.255700

C -4.548200 -5.021700 3.915600

H -4.969400 -6.017000 3.805700

C -5.384800 -3.902900 3.780000

C -4.818500 -2.629000 3.913000

H -5.445400 -1.751700 3.789700

C -3.456300 -2.466300 4.142100

H -3.034400 -1.470200 4.166900

C -6.853200 -4.072200 3.493700

H -7.037200 -4.973200 2.902800

H -7.251600 -3.217000 2.943500

H -7.427600 -4.165700 4.423400

H -7.136400 -5.653500 -4.083700

H -4.893900 -7.107600 -3.999900

H 1.366000 8.043100 6.403000

H 3.217400 8.950300 4.691500

**A’B‘**

425

title

Zn -1.080100 0.339000 0.550300

Zn 2.039500 0.242400 -2.942900

O -1.041600 -2.931100 0.943700

H -2.207700 -1.933400 0.446700

N 0.252800 -0.995000 1.098000

C 0.054100 -2.353300 1.132000

C 1.345000 -2.971400 1.443000

C 2.287400 -1.965100 1.592200

C 1.585200 -0.689300 1.392300

C 1.938000 -4.274200 1.697700

C 1.511800 -5.588400 1.789100

H 0.476100 -5.852900 1.614900

C 2.456600 -6.598400 2.109400

H 2.140100 -7.632600 2.173500

C 3.794100 -6.308600 2.348900

C 5.546100 -4.548100 2.611200

C 5.830300 -3.189900 2.648200

H 6.829700 -2.882900 2.937400

C 4.856100 -2.201100 2.348500

H 5.137300 -1.164700 2.438400

C 3.577800 -2.588000 1.966600

C 3.303200 -3.987500 1.972700

C 4.231600 -4.962400 2.296600

C 2.096900 0.590000 1.552000

C 3.554500 0.772100 1.785900

C 4.035400 1.148100 3.048400

H 3.331600 1.285100 3.862800

C 5.398500 1.346300 3.263000

H 5.748700 1.637800 4.249200

C 6.322100 1.185600 2.220500

C 5.835300 0.800100 0.966400

H 6.536300 0.652600 0.157700

C 4.473400 0.599300 0.746600

H 4.109700 0.307900 -0.229800

C 7.792100 1.462900 2.414900

H 8.015900 2.518100 2.216100

H 8.404100 0.869800 1.729800

H 8.113400 1.249400 3.438100

N -0.058400 1.733200 1.662800

C 1.294600 1.791500 1.590800

C 1.707300 3.176300 1.662500

C 0.527400 3.934800 1.805500

C -0.566600 3.009500 1.864700

C 2.850500 4.101900 1.591000

C 4.224200 4.020200 1.454100

H 4.732100 3.076400 1.337400

C 4.995100 5.215400 1.453200

H 6.073200 5.123600 1.373900

C 4.426100 6.472400 1.521900

C 2.255000 7.810200 1.558700

C 0.871800 7.766500 1.584300

H 0.311700 8.695100 1.532900

C 0.147800 6.549100 1.685200

H -0.934800 6.571100 1.702300

C 0.851400 5.360200 1.748300

C 2.274200 5.410400 1.660100

C 3.008900 6.602600 1.591800

C 4.773700 -7.353500 2.696800

C 6.552800 -5.574400 2.948200

N 6.072000 -6.899800 3.044600

O 4.513800 -8.547900 2.714200

O 7.730800 -5.318000 3.152900

C 6.983100 -7.886100 3.584900

C 6.941200 -8.136100 4.964900

C 7.823900 -9.090500 5.480200

H 7.808500 -9.310600 6.542800

C 8.718200 -9.761400 4.651400

H 9.398300 -10.496900 5.070500

C 8.739400 -9.492300 3.286300

H 9.437800 -10.019000 2.643100

C 7.868400 -8.552100 2.729200

C 5.951500 -7.434900 5.881100

H 5.465100 -6.639800 5.309400

C 6.646700 -6.767700 7.077100

H 5.919100 -6.212500 7.677900

H 7.419600 -6.070900 6.739600

H 7.120400 -7.505500 7.732600

C 4.852100 -8.413400 6.330000

H 4.103400 -7.898900 6.941800

H 5.275300 -9.227400 6.928300

H 4.352800 -8.855300 5.463700

C 7.885600 -8.267900 1.241300

H 7.096300 -7.542100 1.043200

C 9.213800 -7.626500 0.812600

H 9.400200 -6.722200 1.398300

H 9.190600 -7.360000 -0.250500

H 10.055200 -8.310800 0.964200

C 7.558800 -9.529000 0.427500

H 6.605900 -9.956200 0.753000

H 8.330700 -10.296200 0.548300

H 7.491000 -9.295000 -0.640900

C -1.909600 3.217000 2.222100

C -2.286900 4.505600 2.838000

C -3.353500 5.259200 2.328100

H -3.899500 4.887600 1.471500

C -3.689300 6.483100 2.896900

H -4.505900 7.062600 2.475600

C -2.997100 6.977400 4.011000

C -1.945500 6.209600 4.530300

H -1.396300 6.572100 5.394500

C -1.587900 4.997200 3.950500

H -0.764500 4.419500 4.355100

C -3.393600 8.282000 4.653100

H -4.149200 8.120900 5.431500

H -3.822900 8.972400 3.921900

H -2.537500 8.770800 5.126200

N -2.765700 1.139400 1.224100

C -2.918500 2.232100 2.076900

C -4.191700 2.109200 2.701400

C -4.797500 0.927200 2.174600

C -3.868000 0.374400 1.268400

C -5.041300 2.626900 3.780500

C -4.999300 3.651200 4.720500

H -4.180600 4.357800 4.752600

C -6.044500 3.763100 5.671200

H -6.017300 4.553700 6.413500

C -7.114700 2.875900 5.707000

C -8.190500 0.838700 4.738600

C -8.129000 -0.177900 3.794100

H -8.909900 -0.930600 3.804400

C -7.071400 -0.273100 2.853600

H -7.056700 -1.106900 2.160700

C -6.062100 0.679900 2.868800

C -6.150100 1.729900 3.830100

C -7.173800 1.824600 4.761800

C -3.942700 -0.799500 0.293900

O -2.596600 -1.153700 -0.065700

C -4.653600 -0.254100 -0.948000

C -3.919500 0.196700 -2.047800

H -2.845700 0.061700 -2.075700

C -4.569200 0.859900 -3.089400

H -3.991700 1.216800 -3.932700

C -5.948700 1.088900 -3.069700

C -6.675800 0.602400 -1.975500

H -7.750000 0.758900 -1.931800

C -6.038600 -0.053800 -0.926900

H -6.620400 -0.398100 -0.079600

C -6.625200 1.845800 -4.184300

H -7.105100 1.172700 -4.900500

H -5.907100 2.449000 -4.745100

H -7.406400 2.506900 -3.797600

N -4.576200 -2.161500 2.262700

H -4.227600 -1.461900 2.901800

C -4.644700 -1.974000 0.905100

C -5.350600 -3.051700 0.380400

C -5.713900 -3.898500 1.486900

C -5.239900 -3.314700 2.659100

C -5.936900 -3.615500 -0.846300

C -6.002700 -3.269700 -2.182300

H -5.508600 -2.385000 -2.567000

C -6.750100 -4.100200 -3.064900

H -6.839500 -3.812000 -4.105300

C -7.392800 -5.251300 -2.644100

C -7.960900 -6.772400 -0.672700

C -7.851100 -6.987400 0.690300

H -8.339800 -7.852500 1.128200

C -7.130000 -6.111300 1.547600

H -7.075000 -6.286400 2.616000

C -6.508600 -5.014700 0.986800

C -6.615300 -4.799300 -0.419400

C -7.339400 -5.641300 -1.276000

C -9.249100 0.892300 5.761600

C -8.175800 2.973500 6.726800

N -9.174100 1.973100 6.675100

O -10.147400 0.068100 5.852800

O -8.210700 3.843300 7.585100

C -10.203900 2.036600 7.687900

C -9.957100 1.447100 8.934900

C -10.965600 1.512600 9.900300

H -10.800900 1.063200 10.874800

C -12.175700 2.143000 9.625700

H -12.950800 2.183300 10.385200

C -12.394100 2.721200 8.378800

H -13.341400 3.208600 8.170700

C -11.410100 2.682000 7.385900

C -8.645000 0.747100 9.240700

H -8.007700 0.841900 8.358300

C -7.903600 1.423900 10.403100

H -6.933600 0.943100 10.571800

H -8.474000 1.355400 11.335200

H -7.735800 2.481600 10.182500

C -8.864100 -0.754600 9.483700

H -9.367900 -1.212300 8.627300

H -9.483100 -0.926700 10.370200

H -7.907800 -1.265600 9.639700

C -11.668000 3.302900 6.023800

H -10.743100 3.237100 5.444300

C -12.743500 2.510300 5.262200

H -12.450900 1.461100 5.170500

H -12.889100 2.923500 4.258200

H -13.705200 2.551000 5.784900

C -12.023200 4.793000 6.139600

H -11.236500 5.337900 6.669400

H -12.962000 4.941700 6.683100

H -12.142900 5.235700 5.145100

C -5.573100 -3.618800 4.050000

O -6.229100 -4.619600 4.336400

C -5.249000 -2.569900 5.072500

C -6.281400 -2.157100 5.920200

H -7.234400 -2.673000 5.876000

C -6.102400 -1.071500 6.772400

H -6.925500 -0.748600 7.400200

C -4.889000 -0.376800 6.815700

C -3.836200 -0.835200 6.010500

H -2.872800 -0.334500 6.053500

C -4.009200 -1.915100 5.150000

H -3.170400 -2.264200 4.553400

C -4.713800 0.839600 7.688000

H -4.587100 1.738500 7.075700

H -3.827500 0.748900 8.325000

H -5.580500 0.999500 8.333600

O -0.639200 0.820800 -1.350600

N 1.414400 1.745500 -1.847200

C 0.103700 1.830000 -1.523600

C -0.268400 3.233200 -1.488700

C 0.828300 3.988200 -1.880600

C 1.935700 3.025100 -2.079700

C -1.449500 4.053400 -1.333300

C -2.787800 3.824600 -1.073400

H -3.162400 2.830700 -0.856700

C -3.676000 4.932300 -1.121700

H -4.729700 4.757800 -0.925900

C -3.246000 6.215600 -1.421600

C -1.303100 7.693100 -2.160900

C 0.021500 7.725700 -2.556800

H 0.442300 8.657700 -2.921600

C 0.864900 6.581700 -2.523100

H 1.874800 6.669100 -2.894100

C 0.369200 5.386900 -2.033900

C -1.012800 5.367400 -1.657400

C -1.875200 6.466000 -1.722500

C -6.717700 -2.367300 -6.629600

C -6.701200 0.024900 -7.440500

N -7.322300 -1.224600 -7.196300

O -7.348600 -3.407400 -6.496900

O -7.324600 0.938000 -7.959400

C -8.724900 -1.337200 -7.534100

C -9.674400 -1.181400 -6.512700

C -11.023900 -1.300300 -6.858300

H -11.781500 -1.192600 -6.088700

C -11.407300 -1.557300 -8.171200

H -12.460500 -1.643200 -8.421300

C -10.443000 -1.708400 -9.162900

H -10.748700 -1.910900 -10.184500

C -9.081400 -1.607400 -8.861800

C -9.280200 -0.918300 -5.067900

H -8.195100 -0.787800 -5.027200

C -9.914800 0.377100 -4.538900

H -9.573300 0.579000 -3.518600

H -11.006600 0.303800 -4.512700

H -9.650000 1.231500 -5.168100

C -9.626000 -2.121000 -4.173800

H -9.235600 -1.973000 -3.161900

H -9.192000 -3.037700 -4.577500

H -10.710500 -2.258200 -4.103900

C -8.041800 -1.788600 -9.954700

H -7.052300 -1.719600 -9.494700

C -8.144500 -3.182700 -10.593500

H -7.351500 -3.323300 -11.335500

H -9.104200 -3.320000 -11.102500

H -8.051400 -3.964200 -9.833800

C -8.140600 -0.666000 -11.000000

H -8.034300 0.310600 -10.521000

H -9.106400 -0.690900 -11.516000

H -7.355100 -0.774000 -11.755600

C 3.263400 3.218800 -2.423600

C 3.795400 4.582000 -2.634300

C 3.655600 5.581800 -1.667300

H 3.125400 5.365400 -0.751100

C 4.202300 6.847500 -1.872700

H 4.079700 7.603200 -1.103800

C 4.904200 7.151800 -3.043800

C 5.034400 6.147100 -4.015800

H 5.569400 6.361700 -4.936800

C 4.494800 4.882100 -3.815600

H 4.602400 4.115000 -4.575600

C 5.530900 8.507500 -3.248400

H 5.117200 9.246500 -2.557400

H 5.376200 8.869200 -4.269600

H 6.613900 8.468200 -3.081200

N 3.897000 0.838700 -2.774500

C 4.223500 2.128000 -2.538100

C 5.645000 2.192900 -2.297200

C 6.148500 0.879200 -2.381400

C 5.006600 0.006000 -2.603000

C 6.689200 3.086000 -1.779200

C 6.809000 4.409600 -1.378300

H 5.993200 5.108200 -1.470300

C 8.021200 4.855800 -0.792100

H 8.107100 5.883000 -0.454100

C 9.109200 4.013800 -0.610200

C 10.053000 1.721800 -0.877200

C 9.851000 0.419600 -1.311400

H 10.659600 -0.292900 -1.189400

C 8.620200 -0.005100 -1.873300

H 8.526600 -1.030400 -2.188500

C 7.570000 0.894800 -1.991600

C 7.822000 2.243500 -1.607800

C 9.016400 2.669700 -1.042500

C 4.909600 -1.384100 -2.406000

C 6.169900 -2.112800 -2.073500

C 6.558600 -2.251700 -0.735300

H 5.931900 -1.842100 0.045500

C 7.728500 -2.929300 -0.404400

H 7.999800 -3.050100 0.640100

C 8.543600 -3.490400 -1.398100

C 8.139000 -3.361300 -2.734400

H 8.745800 -3.801300 -3.520400

C 6.966200 -2.681100 -3.072700

H 6.666700 -2.594300 -4.112100

C 9.820500 -4.201800 -1.030600

H 10.139900 -4.885900 -1.821100

H 9.701400 -4.774700 -0.107500

H 10.634100 -3.485400 -0.865700

N 2.486800 -1.655100 -2.737200

C 3.702100 -2.169100 -2.382600

C 3.508400 -3.556400 -2.009700

C 2.118700 -3.810100 -2.103900

C 1.514000 -2.629000 -2.662000

C 4.176800 -4.805000 -1.608900

C 5.470400 -5.259200 -1.436300

H 6.324000 -4.626400 -1.591200

C 5.694400 -6.607200 -1.055900

H 6.721400 -6.935400 -0.945700

C 4.665400 -7.500800 -0.827500

C 2.152700 -7.863200 -0.810300

C 0.902100 -7.297100 -0.984100

H 0.015400 -7.894700 -0.796500

C 0.726000 -5.951000 -1.394400

H -0.273300 -5.550800 -1.461600

C 1.843200 -5.179400 -1.663700

C 3.123800 -5.748600 -1.412600

C 3.320300 -7.077000 -1.007500

C 10.340700 4.459800 0.073100

C 11.301600 2.139700 -0.204600

N 11.341500 3.476900 0.248500

O 10.501300 5.594800 0.494300

O 12.243900 1.387000 -0.013700

C 12.489200 3.856100 1.042700

C 13.591600 4.445600 0.413400

C 14.681500 4.802800 1.213400

H 15.550100 5.264700 0.754900

C 14.666100 4.573500 2.586000

H 15.521900 4.855500 3.192000

C 13.556400 3.982700 3.184300

H 13.552900 3.804700 4.255100

C 12.443800 3.613700 2.423000

C 13.616100 4.703800 -1.082700

H 12.688000 4.309900 -1.506200

C 13.648100 6.212200 -1.376800

H 12.795800 6.710600 -0.907000

H 13.610000 6.395200 -2.456100

H 14.565000 6.671100 -0.991800

C 14.775700 3.955800 -1.758500

H 14.713900 2.883600 -1.551300

H 15.746100 4.314500 -1.400000

H 14.746700 4.103000 -2.843300

C 11.240700 2.961500 3.084400

H 10.478600 2.802400 2.317800

C 10.617800 3.877400 4.149000

H 9.714900 3.419400 4.566900

H 10.347300 4.844300 3.714900

H 11.311200 4.060400 4.976200

C 11.605900 1.579600 3.649700

H 10.721600 1.089800 4.071700

H 12.354200 1.661800 4.444800

H 12.016100 0.940500 2.863100

C 0.228500 -2.481500 -3.250500

C -0.733100 -3.608900 -3.109100

C -1.751600 -3.496800 -2.155000

H -1.832400 -2.599000 -1.558800

C -2.661800 -4.532700 -1.976200

H -3.434800 -4.432400 -1.223000

C -2.609900 -5.687200 -2.771300

C -1.593600 -5.783400 -3.732300

H -1.532800 -6.668200 -4.359100

C -0.655000 -4.761400 -3.896400

H 0.134500 -4.859100 -4.634200

C -3.652900 -6.762500 -2.603400

H -4.643500 -6.384400 -2.880000

H -3.721600 -7.086100 -1.560000

H -3.434100 -7.637100 -3.221400

N 0.497200 -0.178300 -4.091100

C -0.224500 -1.359200 -3.980900

C -1.458200 -1.171700 -4.704300

C -1.443900 0.158600 -5.195400

C -0.219900 0.736400 -4.767700

C -2.713100 -1.807900 -5.126600

C -3.331500 -3.043300 -5.012800

H -2.857800 -3.875800 -4.524100

C -4.629000 -3.229200 -5.550000

H -5.108000 -4.195400 -5.450800

C -5.325500 -2.204600 -6.174000

C -5.295600 0.147600 -7.002000

C -4.570400 1.322700 -7.159000

H -5.047800 2.151900 -7.669600

C -3.253700 1.469300 -6.650300

H -2.738400 2.412100 -6.783600

C -2.673000 0.417700 -5.953200

C -3.408800 -0.793900 -5.848100

C -4.691500 -0.952400 -6.351100

O 1.600700 2.204600 -4.924900

C 0.372000 2.088700 -4.925300

C -0.510800 3.272900 -5.024100

C -0.003600 4.455700 -5.584300

H 1.012300 4.455300 -5.963800

C -0.789100 5.598000 -5.633600

H -0.388300 6.509000 -6.068700

C -2.093100 5.602100 -5.111800

C -2.579200 4.429100 -4.525300

H -3.563300 4.424700 -4.068800

C -1.799600 3.279200 -4.475500

H -2.176200 2.399300 -3.970600

C -2.942400 6.843300 -5.179800

H -3.357700 6.980900 -6.185300

H -2.353000 7.733300 -4.942200

H -3.776900 6.789100 -4.476900

H -1.914700 8.589100 -2.215600

H -3.959700 7.034000 -1.453300

H 4.877100 -8.516700 -0.510500

H 2.250200 -8.898300 -0.496800

H 2.770600 8.764800 1.500100

H 5.051600 7.360200 1.499600

H -7.955900 -5.846800 -3.357000

H -8.531200 -7.462000 -1.288800

**B**

211

title

Zn 0.186700 0.465200 -0.654800

O -1.155800 -1.575800 1.546900

N -1.258000 0.582800 0.676100

C -1.788600 -0.559600 1.282100

C -3.232100 -0.301100 1.457500

C -3.530500 0.929000 0.907400

C -2.246700 1.511600 0.448700

C -4.443000 -1.000500 1.855200

C -4.775500 -2.226800 2.408100

C -6.147200 -2.551100 2.586300

C -7.163700 -1.680800 2.207700

C -7.785100 0.488100 1.133400

C -7.339100 1.643800 0.507600

C -5.957700 1.947400 0.362800

C -5.005800 1.075900 0.870900

C -5.491000 -0.118700 1.480000

C -6.830600 -0.433900 1.623400

C -8.592300 -2.005500 2.408800

C -9.220600 0.183200 1.309500

N -9.518700 -1.013900 2.000000

O -8.982600 -3.049400 2.906500

O -10.116000 0.914700 0.913400

C -10.904900 -1.226900 2.362300

C -11.371600 -0.644700 3.550300

C -12.715100 -0.838500 3.886200

C -13.555800 -1.590000 3.070700

C -13.063900 -2.162400 1.901700

C -11.728300 -1.991800 1.526600

C -10.476100 0.200000 4.442300

C -10.878600 1.682700 4.360400

C -10.465900 -0.310800 5.891000

C -11.205700 -2.600900 0.240300

C -11.404400 -4.122700 0.205900

C -11.822700 -1.903200 -0.982700

C -2.044100 2.772300 -0.122500

C -3.208200 3.684800 -0.267100

C -3.780100 4.320700 0.840800

C -4.868700 5.178500 0.683500

C -5.418500 5.422300 -0.581700

C -4.846800 4.774400 -1.687300

C -3.756000 3.921500 -1.535900

C -6.577200 6.371500 -0.757600

N 0.308200 2.432700 -0.694700

C -0.769800 3.244900 -0.580200

C -0.354000 4.598900 -0.926400

C 1.015400 4.540800 -1.216400

C 1.445000 3.171200 -0.993000

C -0.816500 5.984200 -1.098800

C -1.988300 6.698700 -0.928600

C -2.008900 8.085200 -1.240700

C -0.901900 8.757700 -1.721400

C 1.543300 8.593000 -2.404900

C 2.650200 7.775500 -2.548100

C 2.635500 6.402900 -2.187600

C 1.473500 5.856700 -1.669800

C 0.328100 6.698500 -1.565000

C 0.324300 8.058100 -1.903400

C 2.734900 2.644800 -0.924400

C 3.880500 3.585000 -0.988800

C 4.050200 4.567700 -0.004600

C 5.127900 5.447600 -0.061000

C 6.060800 5.381300 -1.104400

C 5.882800 4.399400 -2.089400

C 4.816800 3.505800 -2.028600

C 7.242400 6.316300 -1.150100

N 2.140200 0.261100 -0.982100

C 3.051200 1.249800 -0.754300

C 4.302200 0.637600 -0.400700

C 4.102500 -0.763800 -0.451900

C 2.747000 -0.975400 -0.877600

C 5.662100 0.911200 0.087600

C 6.443100 2.014000 0.406900

C 7.754800 1.821500 0.911300

C 8.288700 0.558200 1.124600

C 7.914600 -1.907700 1.085100

C 7.032100 -2.950500 0.837800

C 5.729200 -2.731100 0.323400

C 5.317600 -1.438000 0.028900

C 6.224800 -0.378100 0.316100

C 7.497000 -0.578900 0.835700

C 9.647200 0.361500 1.666800

C 9.268300 -2.138900 1.628200

N 10.042300 -0.981200 1.873100

O 10.409100 1.280000 1.930100

O 9.720200 -3.249900 1.861900

C 11.372800 -1.191200 2.398800

C 12.442900 -1.292300 1.500100

C 13.721500 -1.492000 2.029100

C 13.918500 -1.591600 3.403200

C 12.836500 -1.489900 4.272600

C 11.541700 -1.285100 3.786400

C 12.243600 -1.175000 -0.001300

C 12.910600 0.101000 -0.540700

C 12.729100 -2.436100 -0.732200

C 10.369500 -1.180900 4.746900

C 10.547800 -0.004600 5.719300

C 10.144400 -2.512600 5.481100

C 2.100800 -2.174300 -1.285100

C 2.922000 -3.411200 -1.303700

C 2.664100 -4.443100 -0.393000

C 3.448200 -5.595000 -0.397100

C 4.490800 -5.758200 -1.319800

C 4.726400 -4.728700 -2.241700

C 3.962000 -3.565000 -2.228800

C 5.354800 -6.993600 -1.304400

N -0.145100 -1.235000 -1.612700

C 0.765000 -2.279500 -1.721300

C 0.078200 -3.385300 -2.353400

C -1.253500 -2.961400 -2.564400

C -1.340600 -1.627300 -2.085500

C 0.237300 -4.717400 -2.954100

C 1.257100 -5.634100 -3.142400

C 0.981800 -6.846900 -3.828200

C -0.268700 -7.152600 -4.333000

C -2.664700 -6.366200 -4.681200

C -3.585600 -5.352000 -4.493200

C -3.265900 -4.150700 -3.804300

C -1.991800 -3.994500 -3.292100

C -1.047000 -5.040700 -3.495300

C -1.338300 -6.226500 -4.182700

O -2.165500 0.559300 -2.276600

C -2.440300 -0.634000 -2.115800

C -3.840900 -1.079300 -1.936900

C -4.878600 -0.235400 -2.360300

C -6.202400 -0.600300 -2.163400

C -6.528700 -1.804800 -1.521900

C -5.488600 -2.625100 -1.069700

C -4.158900 -2.272500 -1.273300

C -7.965700 -2.207300 -1.333300

H -4.007100 -2.936700 2.694600

H -6.428700 -3.501300 3.027200

H -8.080800 2.334000 0.120000

H -5.684200 2.849300 -0.160300

H -13.105900 -0.393500 4.795700

H -14.596500 -1.729800 3.347400

H -13.723700 -2.747900 1.269200

H -9.451600 0.119800 4.068500

H -11.893600 1.833200 4.743300

H -10.853100 2.031900 3.324800

H -10.198600 2.300700 4.956600

H -10.168000 -1.362500 5.930800

H -11.452300 -0.221700 6.357200

H -9.761700 0.271100 6.494600

H -10.132000 -2.419100 0.207100

H -12.465600 -4.392700 0.208500

H -10.931400 -4.588000 1.074900

H -10.958100 -4.545000 -0.701000

H -11.637900 -0.825900 -0.942000

H -12.906100 -2.058600 -1.021700

H -11.393400 -2.298000 -1.910100

H -3.365900 4.139900 1.827200

H -5.300000 5.662800 1.555000

H -5.263300 4.941900 -2.676800

H -3.324300 3.416300 -2.393700

H -6.228000 7.365600 -1.061800

H -7.140100 6.491400 0.171800

H -7.266300 6.020800 -1.531500

H -2.889400 6.237500 -0.558100

H -2.939500 8.626300 -1.098500

H -0.961100 9.815700 -1.960200

H 1.596800 9.641400 -2.684600

H 3.570300 8.191600 -2.947000

H 3.529200 5.812400 -2.333000

H 3.330100 4.637100 0.803400

H 5.244300 6.201300 0.712300

H 6.592600 4.331300 -2.908800

H 4.697300 2.745800 -2.793600

H 8.117100 5.865000 -0.666500

H 7.029400 7.254600 -0.631100

H 7.526400 6.550200 -2.180000

H 6.075000 3.022500 0.297400

H 8.368600 2.682100 1.155000

H 7.360900 -3.960100 1.059200

H 5.076900 -3.580000 0.186800

H 14.570300 -1.569800 1.356800

H 14.918000 -1.748600 3.797700

H 12.996900 -1.570000 5.343200

H 11.170900 -1.085700 -0.193000

H 13.995700 0.070800 -0.395400

H 12.523300 0.982000 -0.021800

H 12.718700 0.213000 -1.613400

H 12.223000 -3.324500 -0.343500

H 13.807600 -2.580600 -0.610100

H 12.523600 -2.360400 -1.805300

H 9.469400 -0.978700 4.160400

H 10.682400 0.931100 5.169200

H 11.421200 -0.147000 6.364200

H 9.669500 0.094900 6.365700

H 11.005700 -2.770100 6.106800

H 9.992200 -3.322900 4.763000

H 9.264800 -2.450500 6.130800

H 1.851700 -4.333100 0.317500

H 3.243100 -6.383300 0.321300

H 5.523700 -4.837300 -2.971300

H 4.163000 -2.770700 -2.939700

H 5.684900 -7.261800 -2.311900

H 4.822500 -7.848100 -0.878500

H 6.255300 -6.833000 -0.699600

H 2.261100 -5.452300 -2.792800

H 1.794700 -7.554800 -3.959700

H -0.435500 -8.090800 -4.854400

H -2.945700 -7.267900 -5.217500

H -4.590600 -5.468500 -4.887200

H -4.021300 -3.382500 -3.690400

H -4.619800 0.705900 -2.831900

H -6.999900 0.059300 -2.492600

H -5.724800 -3.539600 -0.535000

H -3.365600 -2.906300 -0.894500

H -8.354600 -2.710900 -2.226700

H -8.601200 -1.336500 -1.153800

H -8.075900 -2.898700 -0.494900

**B‘**

211

title

Zn 0.049800 0.589100 -1.303600

O -1.811800 -0.381200 -3.811100

N 0.186000 -0.799500 -2.691300

C -0.930700 -1.157000 -3.459700

C -0.841300 -2.614900 -3.670000

C 0.260700 -3.092000 -2.992800

C 0.937400 -1.911100 -2.400700

C -1.644200 -3.706100 -4.185900

C -2.832700 -3.827600 -4.878700

H -3.380000 -2.951200 -5.209700

C -3.335700 -5.131700 -5.133800

H -4.267700 -5.231900 -5.681500

C -2.682900 -6.274100 -4.699400

C -0.713400 -7.238700 -3.395400

C 0.413500 -6.954300 -2.646400

H 0.969900 -7.770100 -2.194700

C 0.882800 -5.629000 -2.430600

H 1.750200 -5.484000 -1.807200

C 0.201300 -4.569600 -3.000300

C -0.972800 -4.883000 -3.753400

C -1.462400 -6.173800 -3.970700

C -9.305100 0.776100 2.015100

C -8.884100 -1.705200 2.279500

N -9.678100 -0.537800 2.380900

O -10.082600 1.707400 2.162000

O -9.323900 -2.786800 2.639700

C -11.008500 -0.705700 2.921700

C -12.054500 -1.028300 2.047100

C -13.332500 -1.189100 2.590900

H -14.161300 -1.443700 1.937800

C -13.553800 -1.027600 3.955400

H -14.553100 -1.154200 4.361300

C -12.496300 -0.704600 4.800500

H -12.676100 -0.578400 5.863500

C -11.201400 -0.539900 4.299500

C -11.825500 -1.217500 0.557400

H -10.778700 -0.984500 0.344900

C -12.057300 -2.682700 0.152900

H -11.837800 -2.828900 -0.910200

H -13.097500 -2.979600 0.324700

H -11.414100 -3.345800 0.737700

C -12.680800 -0.245600 -0.269900

H -12.459400 -0.353400 -1.337100

H -12.480100 0.788700 0.023700

H -13.750400 -0.435100 -0.131900

C -10.057900 -0.181400 5.232800

H -9.137400 -0.154300 4.643700

C -10.258900 1.221800 5.828100

H -9.406000 1.499700 6.456600

H -11.160200 1.262300 6.449100

H -10.362400 1.962700 5.030800

C -9.866600 -1.250800 6.319300

H -9.703500 -2.233000 5.866800

H -10.742900 -1.320000 6.972300

H -9.002300 -1.009100 6.947000

C 2.126600 -1.893400 -1.658000

C 2.866700 -3.165900 -1.456000

C 3.573700 -3.775300 -2.499200

H 3.573400 -3.312200 -3.480400

C 4.271500 -4.963900 -2.284800

H 4.815000 -5.422000 -3.106200

C 4.280800 -5.578600 -1.025700

C 3.560900 -4.969400 0.012900

H 3.546500 -5.434000 0.994900

C 2.866600 -3.779700 -0.195400

H 2.302000 -3.319800 0.609000

C 5.064400 -6.844300 -0.783600

H 5.216400 -7.403700 -1.710500

H 4.556000 -7.497500 -0.068500

H 6.054800 -6.620100 -0.369500

N 1.993500 0.458100 -0.992400

C 2.674600 -0.712700 -1.067500

C 4.005100 -0.488500 -0.514700

C 4.070300 0.862800 -0.145300

C 2.809200 1.472700 -0.521800

C 5.288000 -1.134200 -0.190600

C 5.892400 -2.374900 -0.337000

H 5.394400 -3.195300 -0.827900

C 7.205800 -2.580100 0.162300

H 7.676400 -3.550800 0.049400

C 7.918800 -1.579300 0.806400

C 7.952400 0.783400 1.609400

C 7.271500 1.988100 1.726400

H 7.762500 2.803400 2.246500

C 5.972600 2.175100 1.188400

H 5.487000 3.131600 1.320100

C 5.354800 1.128300 0.516700

C 6.050400 -0.110100 0.442900

C 7.324900 -0.303000 0.955700

C 2.458500 2.825200 -0.564400

C 3.520000 3.825000 -0.295600

C 4.644200 3.908400 -1.129100

H 4.723900 3.237800 -1.977700

C 5.643400 4.844500 -0.879100

H 6.503800 4.898600 -1.539900

C 5.560800 5.713900 0.217200

C 4.431700 5.630100 1.043800

H 4.342000 6.300800 1.893400

C 3.420100 4.707500 0.788200

H 2.547200 4.662400 1.430900

C 6.667900 6.693600 0.511800

H 6.299900 7.555700 1.074400

H 7.133500 7.057400 -0.408500

H 7.457000 6.223000 1.110600

N 0.042400 2.531600 -0.857200

C 1.149900 3.324200 -0.888900

C 0.744200 4.665300 -1.231000

C -0.658400 4.642800 -1.377500

C -1.089500 3.299300 -1.073400

C 1.241400 6.001200 -1.588200

C 2.466400 6.633900 -1.700300

H 3.393700 6.128700 -1.476800

C 2.511600 7.984000 -2.140700

H 3.482000 8.465700 -2.213200

C 1.377100 8.692000 -2.488200

C -1.144600 8.626700 -2.807700

C -2.295000 7.861600 -2.751300

H -3.237300 8.293500 -3.074500

C -2.296200 6.518400 -2.292300

H -3.222300 5.963200 -2.304600

C -1.108900 5.952600 -1.861700

C 0.076900 6.741100 -1.953000

C 0.102200 8.063700 -2.417500

C 9.276900 -1.799600 1.342900

C 9.311200 0.593800 2.159200

N 9.877200 -0.689200 1.982200

O 9.871400 -2.863300 1.257600

O 9.932500 1.474700 2.734100

C 11.208000 -0.888700 2.512000

C 11.346300 -1.387400 3.814000

C 12.641800 -1.574500 4.305400

H 12.779700 -1.963400 5.309400

C 13.753000 -1.269000 3.525100

H 14.752300 -1.418400 3.923000

C 13.585600 -0.772500 2.235800

H 14.456700 -0.534500 1.633600

C 12.308100 -0.575200 1.703000

C 10.142700 -1.732400 4.674200

H 9.241400 -1.458300 4.119300

C 10.074500 -3.246200 4.934300

H 10.052500 -3.795000 3.988900

H 9.175900 -3.498700 5.507400

H 10.943800 -3.588300 5.505800

C 10.134400 -0.919200 5.977800

H 10.161400 0.152800 5.762400

H 10.998300 -1.160100 6.606000

H 9.231000 -1.134700 6.558100

C 12.140300 -0.024600 0.297200

H 11.071700 -0.008200 0.066500

C 12.812500 -0.932200 -0.744500

H 12.631200 -0.554100 -1.756200

H 12.418700 -1.950400 -0.678500

H 13.896400 -0.978200 -0.596400

C 12.646300 1.425000 0.215900

H 12.473400 1.838500 -0.783600

H 13.720800 1.480100 0.420200

H 12.129400 2.051200 0.948200

C -2.390800 2.793500 -0.862500

C -3.521500 3.752200 -0.936900

C -4.463700 3.653000 -1.969000

H -4.358300 2.867500 -2.709800

C -5.514400 4.563700 -2.050200

H -6.229500 4.483400 -2.863800

C -5.670800 5.576400 -1.092900

C -4.730600 5.659000 -0.057000

H -4.830500 6.437200 0.694000

C -3.664100 4.766200 0.017700

H -2.934700 4.851300 0.815900

C -6.837300 6.529100 -1.159900

H -7.716700 6.106500 -0.659300

H -7.122600 6.737800 -2.194800

H -6.606200 7.478300 -0.669200

N -1.787900 0.420100 -0.587300

C -2.711100 1.451200 -0.530800

C -3.956100 0.890600 -0.074000

C -3.733900 -0.501300 0.091900

C -2.378500 -0.741400 -0.240800

C -5.319400 1.199500 0.388800

C -6.128300 2.316400 0.555200

H -5.786800 3.308000 0.304200

C -7.435900 2.164000 1.083200

H -8.069300 3.035700 1.208400

C -7.945700 0.928900 1.460000

C -7.530400 -1.518900 1.721600

C -6.636700 -2.573900 1.588700

H -6.960700 -3.558400 1.908600

C -5.332000 -2.395000 1.060900

H -4.670100 -3.248500 0.983500

C -4.933000 -1.130800 0.650600

C -5.856800 -0.058700 0.797100

C -7.130900 -0.220000 1.323900

O -0.354400 -1.855100 0.155500

C -1.544500 -1.962500 -0.159200

C -2.145200 -3.282200 -0.457900

C -1.508900 -4.439800 0.015300

H -0.605600 -4.329300 0.604500

C -2.024700 -5.692900 -0.280200

H -1.522700 -6.582900 0.087100

C -3.174900 -5.830700 -1.072400

C -3.785800 -4.673600 -1.568400

H -4.650300 -4.763400 -2.217900

C -3.280600 -3.412800 -1.269500

H -3.747900 -2.529000 -1.688000

C -3.738100 -7.193800 -1.375600

H -4.382900 -7.542600 -0.559800

H -2.938400 -7.929100 -1.500100

H -4.335900 -7.179500 -2.290000

H -1.037300 -8.266600 -3.532000

H -3.106200 -7.253200 -4.905100

H 1.453800 9.719800 -2.831200

H -1.183900 9.650400 -3.169100

**H2O**

3

title

O -0.000000 0.000000 0.119200

H -0.000000 0.759300 -0.476800

H -0.000000 -0.759300 -0.476800

**O2**

2

title

O 0.000000 0.000000 0.607300

O 0.000000 0.000000 -0.607300
